# Discovery and Evaluation of Novel Sulfonamide Derivatives Targeting Aromatase in ER+ Breast Cancer

**DOI:** 10.3390/ph18081206

**Published:** 2025-08-15

**Authors:** Barbara De Filippis, Mariangela Agamennone, Alessandra Ammazzalorso, Rosa Amoroso, Letizia Giampietro, Cristina Maccallini, Begüm Nurpelin Sağlık, Chiara De Simone, Mariachiara Zuccarini, Zafer Asım Kaplancıklı, Marialuigia Fantacuzzi

**Affiliations:** 1 Department of Pharmacy, University “G. D’Annunzio” of Chieti-Pescara, 66100 Chieti, Italy; barbara.defilippis@unich.it (B.D.F.); mariangela.agamennone@unich.it (M.A.); alessandra.ammazzalorso@unich.it (A.A.); rosa.amoroso@unich.it (R.A.); letizia.giampietro@unich.it (L.G.); cristina.maccallini@unich.it (C.M.); 2 Department of Pharmaceutical Chemistry, Faculty of Pharmacy, Anadolu University, 26470 Eskişehir, Turkey; bnsaglik@anadolu.edu.tr (B.N.S.); zakaplan@anadolu.edu.tr (Z.A.K.); 3 Department of Medical, Oral and Biotechnological Sciences, University “G. D’Annunzio” of Chieti-Pescara, 66100 Chieti, Italy; chiara.desimone@unich.it (C.D.S.); mariachiara.zuccarini@unich.it (M.Z.)

**Keywords:** aromatase inhibitor, breast cancer, CYP19A1, in silico study, cancer cell line, sulfonamide derivatives, heterocycles

## Abstract

**Background:** Third-generation aromatase inhibitors (CYP19A1) are the mainstay of treatment for estrogen-receptor-positive breast cancer. This is because estrogen is required for cancer growth in approximately 70% of patients with this condition. Although potent and effective, aromatase inhibitors induce resistance and secondary effects, requiring treatment to be discontinued. This clinical limitation highlights the need to search for new molecules. Previous studies have led to the identification of a set of indole sulfonamide molecules that exhibit interesting activity against aromatase. **Methods:** Phenyl and benzyl sulfonamide derivatives with alkylated heterocycles linked by short methylene bridges were designed and synthesized. The aromatase inhibition and cytotoxicity were tested through in vitro assays. Molecular docking and dynamic simulations evaluated the interactions with the aromatase enzyme, while a target fishing strategy linked to gene associations relevant to breast cancer helped to uncover other targets. **Results:** All of the non-steroidal inhibitors synthesized showed significant activity. Compounds **3** and **9** demonstrated IC_50_ values in the low micromolar range and selective action against MCF7 breast cancer cells over healthy lines. Computational studies confirmed stable and favorable aromatase binding. Target fishing identified EGFR and PTK2B as additional potential targets for a multi-target therapeutic strategy. **Conclusions:** Compounds **3** and **9** outperform indole-based inhibitors in their potency and selectivity, revealing strong therapeutic potential. Their binding affinity and specificity support further development. EGFR and PTK2B may enable a broader, multi-target approach.

## 1. Introduction

Breast cancer is the most prevalent form of cancer among the global female population, though its incidence varies geographically. In 2022, approximately 2.3 million new cases were recorded on a global scale, constituting 23.8% of all cancer-related cases and deaths in women. Should national incidence rates remain stable, it is projected that breast cancer cases and deaths will increase by 54.7% by 2050 [1,2,3].

The highest incidence rates have been observed in high-income countries, including those in North America, Australia, New Zealand, and several regions of Europe, with the figures ranging from 85.8 to 91.6 cases per 100,000 women. These elevated rates are attributed to the widespread implementation of diagnostic and screening programs.

Conversely, lower-income regions, encompassing South–Central Asia and Central Africa, exhibit markedly lower incidence rates, averaging approximately 27 cases per 100,000 women. This is likely attributable to inadequate diagnostic infrastructure and lower levels of health literacy, resulting in late-stage detection.

It is evident from global data that there are marked disparities based on the Human Development Index (HDI). In countries with a very high HDI, the lifetime prevalence of breast cancer among women is 1 in 12, with a mortality rate of 1 in 71. Conversely, in low-HDI countries, the proportion of women diagnosed is only 1 in 27, while the mortality rate is 1 in 48 [2].

These disparities underscore the necessity for enhanced equitable healthcare access and improved early detection capabilities on a global scale.

However, as populations grow and lifestyles evolve, developing countries, many of which are in Asia and Africa, are witnessing a rising trend in the prevalence of breast cancer [4]. By 2040, the incidence of breast cancer is predicted to rise significantly, further contributing to the overall burden of the disease [5]. This fact highlights the urgent need for improved screening, awareness, and access to treatment worldwide.

Breast cancer can be classified into four distinct types based on immunohistochemical markers: endocrine-receptor-positive (estrogen or progesterone receptor, ER+ or PR+), tumors that express estrogen or progesterone receptors; human epidermal growth factor receptor 2-positive (HER2), tumors with overexpression of the human epidermal growth factor receptor 2; triple-positive, tumors that are positive for estrogen, progesterone, and HER2 receptors; and triple-negative, tumors that lack estrogen, progesterone, and HER2 receptors [6].

**Figure 1:**
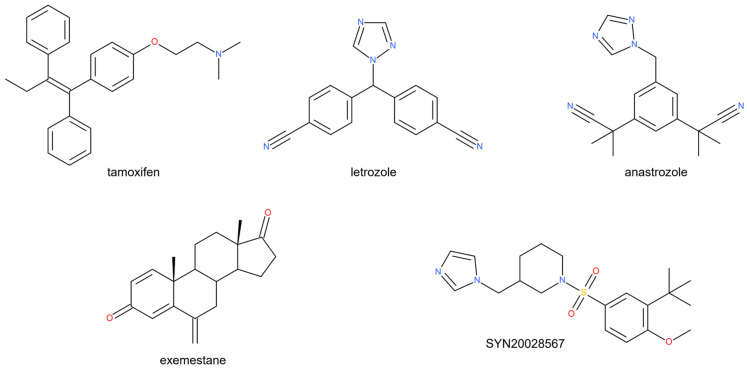
Structure of some aromatase inhibitors in clinical use.

Among this, ER+ breast cancer is characterized by the overexpression of estrogen receptors, which play a crucial role in the progression and treatment of the disease [7]. Endocrine therapy, including agents such as tamoxifen and aromatase inhibitors (Figure 1), is the primary treatment strategy. However, resistance to these therapies often develops over time, necessitating the development of alternative treatment approaches [8].

Aromatase inhibitors (AIs) are a class of drugs that inhibit the enzyme aromatase, which is responsible for converting androgens into estrogens [9]. By significantly reducing estrogen levels, AIs plays a crucial role in the treatment of ER+ breast cancer, particularly in postmenopausal women. Due to their effectiveness, AIs are becoming an increasingly popular choice of hormonal therapy over tamoxifen [10].

There are two main types of AIs from a structural point of view: steroidal and non-steroidal compounds. Steroidal AIs (e.g., exemestane, see Figure 1) bind covalently to a specific pocket of the enzyme, causing irreversible enzyme inhibition, while non-steroidal AIs (e.g., anastrozole and letrozole, Figure 1) form reversible bonds with the enzyme, allowing for competitive androgen inhibition [11].

Non-steroidal AIs contain an azole ring, in which the built-in nitrogen atom interacts with the iron atom in the heme found in aromatase, and an aryl part, which mimics the steroid structure of the natural substrate [12]. Third-generation AIs, including anastrozole, letrozole, and exemestane, are the most potent and selective, effectively lowering estrogenic levels in the serum and increasing follicle-stimulating hormone (FSH) levels in premenopausal women [13]. Beyond breast cancer, AIs are also being investigated for other disorders where estrogen reduction is beneficial, such as endometriosis and male prolactinoma [14]. However, their clinical use may lead to adverse effects, including alterations in lipid metabolism, cardiovascular problems, bone density loss, and musculoskeletal symptoms such as myalgia and arthralgia [15,16]. A recent study has shown that third-generation aromatase inhibitors can also cause a rare disorder known as interstitial lung disease. In particular, letrozole can lead to a high incidence of blood disorders (thrombocytopenia, neutropenia, leukopenia, and hypersensitivity vasculitis), pulmonary embolism, polyneuropathy, and osteonecrosis of the jaw [17]. For this reason, there is a need for alternative therapeutic approaches to enhancing aromatase selectivity, minimizing toxicity, and reducing non-specific adverse effects. By improving selectivity, treatments can target cancer cells more effectively while preserving healthy tissue and leading to better clinical outcomes.

**Figure 2:**
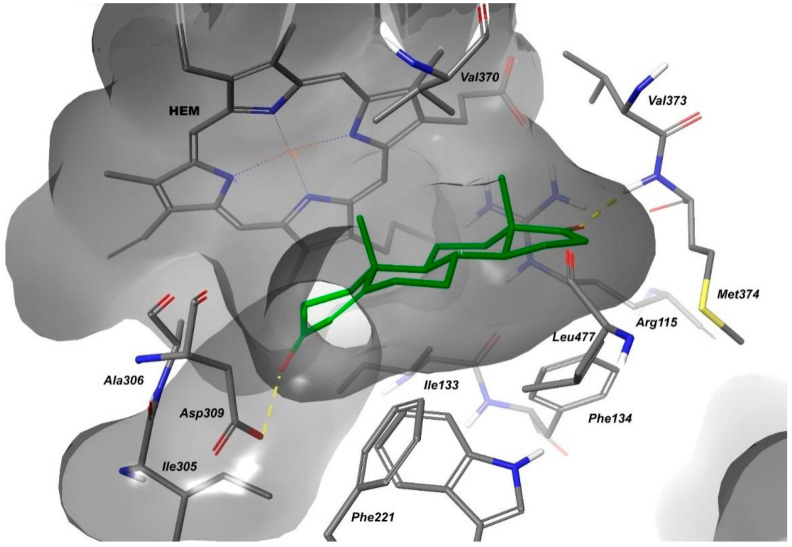
Androstenedione (green stick) inside the active site of the aromatase enzyme (gray), with the heme group and the interacting residues (gray sticks). The active site is shown as a gray surface and the H-bonds as a dotted yellow line. This image was generated using PyMOL 2.5 from the crystallographic structure obtained from the Protein Data Bank (PDB ID 3EQM).

The active site in aromatase consists of an access channel leading into a cavity defined by the heme and key residues, including Arg115, Ile133, Trp224, Ala306, Asp309, Thr310, Val370, Leu372, Val373, Met374, and Leu477. Figure 2 illustrates the positioning of androstenedione within the catalytic site of aromatase. In addition to the hydrophobic interactions of the steroid core within the active site, other specific residues play crucial roles in aromatase’s catalytic function. Met374 forms a hydrogen bond with the ketone group of the D-ring in the steroid core, stabilizing substrate binding. Meanwhile, Asp309 facilitates the aromatization process by donating a proton to the ketone in C3 during A-ring conversion. These residues also serve as critical interaction points for aromatase inhibitors, influencing their binding and effectiveness [18].

Sulfonamide-based compounds exhibit a remarkably broad range of biological activities, making them essential in a variety of therapeutic applications. In fact, the sulfonamide scaffold is present in numerous drug classes, including diuretics, antibacterials, antivirals, anti-inflammatories, and antiepileptics [19,20]. The multiple mechanisms of action exhibited by sulfonamide compounds extend their role as anticancer agents, leading to a broad spectrum of applicability in targeting various cancer types or different receptors for the same cancer, thus acting as multi-target compounds [21,22]. Beyond their structural versatility, sulfonamides hold significant potential in the field of medicinal chemistry, offering the opportunity to design structurally diverse molecules with favorable chemical and physical properties [23]. This adaptability enhances their applicability across various therapeutic fields, reinforcing their importance in drug development.

The sulfonamide pharmacophore is present in some AIs (Figure 1), playing a crucial role in their interaction with target proteins [21,24]. Molecular docking studies revealed that both oxygen and nitrogen atoms from the sulfonamide group can establish key interactions within the active site of the target protein [25]. Additionally, the sulfonamide group serves as a bioisostere for the carboxylic group, offering advantages in drug design. This substitution helps overcome the challenges associated with the carboxylic group, including metabolic instability, potential toxicity, and restricted passive diffusion across cellular membranes. Due to these favorable properties, many sulfonamide-containing compounds are widely utilized as the first-line therapy in anticancer treatment.

**Figure 3:**
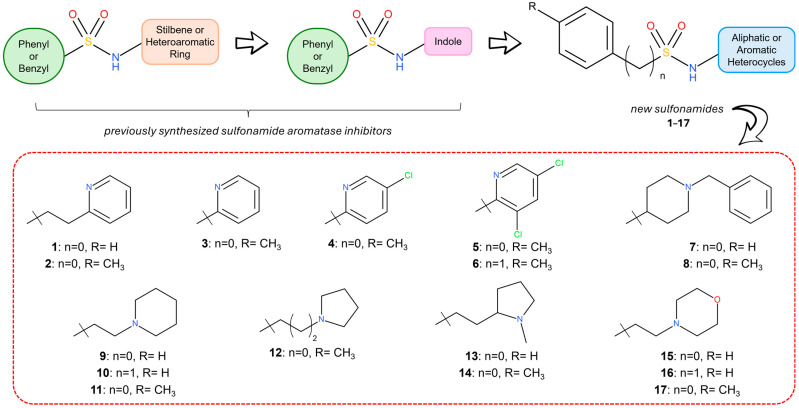
Chemical structures of novel sulfonamide compounds **1**–**17**.

Our research group has been actively exploring new anticancer compounds, focusing on the synthesis of compounds that integrate different heterocycles (as triazole) or planar aromatic systems (stilbene or diaryldiazene moieties) with sulfonamide groups (Figure 3) [26,27,28,29,30]. Among these new compounds, we focused on a set of compounds that combined the sulfonamide group with an indole ring [31,32], a well-established heterocycle in anticancer drugs, due to its ability to target multiple cancer-related pathways, including aromatase inhibition (Figure 3) [26]. Some of these compounds demonstrated strong aromatase inhibition in the sub-micromolar range, exhibiting high selectivity toward non-tumor cells. Molecular docking studies confirmed that the indole group and the sulfonamide linker of the most active compounds both interact with key residues of the aromatase active site. This confirms the effectiveness of combining these functions with a single chemical entity to enhance target engagement.

Starting from these findings, this research aims to expand our understanding of the structure–activity relationships by replacing the indole core with aromatic or aliphatic heterocyclic rings, spaced by a short alkyl chain. Within this scope, a novel series of seventeen sulfonamide derivatives was synthesized, and their ability to inhibit the aromatase enzyme was evaluated both in vitro and in vivo. Additionally, in silico studies were conducted to verify possible binding modes of the most promising molecules. Finally, the ability of the best compounds to interact with other breast cancer targets was explored by combining the results of the target fishing approach with breast-cancer-associated genes. Docking studies revealed a possible binding mode of the sulfonamides in the active site of EGFR and PTK2B.

## 2. Results and Discussion

### 2.1. Structural Design

Previous studies have explored the structural variations in sulfonamide derivatives, where the nitrogen atom of the sulfonamide group was linked to an indole, a stilbene, or a stilbene isostere, while the sulfur atom was attached to an unsubstituted or variously substituted benzyl or phenyl ring (Figure 3). Docking studies on the most active indole derivatives highlighted that the indole nucleus is fundamental to the interaction with the heme group of the aromatase enzyme, while the sulfonamide linker facilitates hydrogen bonding, and the benzene moiety engages with a hydrophobic pocket [26]. Building on these results, a library of seventeen sulfonamide derivatives (**1**–**17**, Figure 3) was synthesized and tested to further investigate the structural requirements for optimal inhibition of the aromatase enzyme. In particular, the aryl moiety linked to the sulfur atom was varied, incorporating phenyl, para-methyl-phenyl, or benzyl rings. Meanwhile, the nitrogen atom of the sulfonamide was connected to different heterocyclic rings, including pyridine, pyrrolidine, piperidine, or morpholine, spaced by distinct alkyl linkers.

### 2.2. Chemistry

**Scheme 1:**
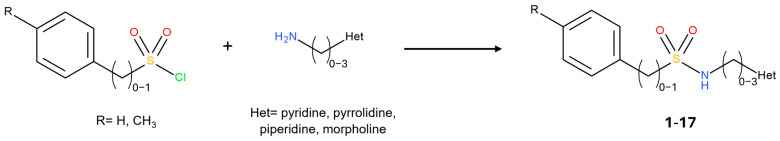
General synthesis of compounds **1**–**17**. Reagents and conditions: heteroaryl or heterocycloalkyl amine (1 eq.), proper sulfonylchloride (1.5 eq.), Et_3_N (3 eq.), or pyridine (5 eq.), 0 °C (2 h) → r.t.

Synthesis of compounds **1**–**17** was carried out through the reaction of the appropriate commercial heteroaryl or heterocycloalkyl amine and the commercial arylsulfonylchloride in the presence of triethylamine or pyridine in dry chloroform, keeping the reaction at 0 °C for 2 h and then at room temperature. Purification on silica gel or through crystallization from ethanol yielded the purified compounds **1**–**17**. The reaction route is presented in Scheme 1. Physical and analytical data for all new synthesized compounds **1**–**17** are reported in Table S1 in the Supporting Material.

### 2.3. Biological Evaluation

#### 2.3.1. In Vitro Aromatase Inhibition

**Figure 4:**
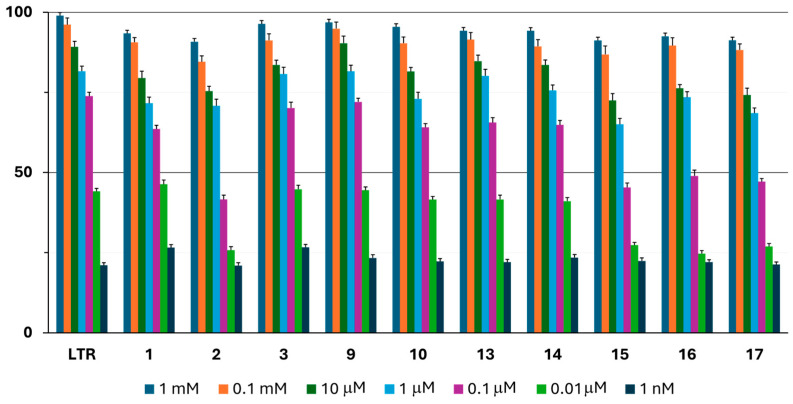
The percentage of inhibition by synthesized compounds **1**–**3**, **9**–**10**, and **13**–**17** and letrozole (LTR) of the aromatase enzyme. Data shown are the means ± standard deviations of four replicates.

The in vitro activity of the compounds against aromatase was evaluated using a commercial fluorometric assay kit (Aromatase CYP19A Inhibitor Screening kit, BioVision, Milpitas, CA, USA), with letrozole (IC_50_ = 1.9 nM) serving as a reference [33,34]. The compounds were tested at seven different concentrations in the scalar range of 1 mM to 1 nM for the IC_50_ calculations, and all experiments were repeated in quadruplicate. The signal of letrozole at 1 μM represented 100% inhibition, while the signal in the absence of any compounds represented 0% inhibition. In Figure 4, the percentage of inhibition of compounds **1**–**3**, **9**–**10**, and **13**–**17** at each tested concentration is shown. Given that compounds **4**–**8** and **11**, **12** exhibited an inhibition percentage of less than 50% at 0.1 mM, they were not subjected to testing at lower concentrations, and as such, their activity is not reported in Figure 4.

Most of the tested molecules have an activity of about 75% inhibition already at 10 μM. The general trend in the activity is similar for compounds **1**–**3**, **9**–**10**, and **13**–**17,** indicating dose–response correlations and success in inhibiting aromatase at sub-micromolar concentrations. Compounds **4**–**8** and **11**–**12**, which contain one or two chlorine atoms on pyridine (**4**–**6**), benzylpiperidine (**7**–**8**), or ethylpiperidine/pyrrolidine and a para-methyl attached to the benzene ring (**11**–**12**), are not able to inhibit the aromatase enzyme, even at a concentration of 10 μM.

To further investigate and better evaluate the different results obtained in the first test, the IC_50_ values were also calculated at both the enzymatic and cellular levels. The calculated IC_50_ values for compounds **1**–**17** are given in Table 1. The most active compounds **1**, **3**, **9**–**10**, and **13**–**14** show IC_50_ values between 30 and 60 nM, values similar to those obtained for the LTR used as a reference, while the others show IC_50_ values in the range of 160–337 nM.

The aromatic or aliphatic nature of the heterocycle present does not influence the ability to inhibit the aromatase enzyme. As will be highlighted below, this result is consistent with their similar target binding mode. Furthermore, the present findings are in line with the documented literature, which reports the occurrence of significant anti-aromatase activity in a variety of non-steroidal heterocyclic functionalities, including azoles, chromenes, coumarins, xanthenes, triphenylethylenes, indoles, pyrimidines, pyridines, quinolones, and thioureas [35].

A structural analysis of the compounds reveals that the optimal heterocycles are unsubstituted 2-pyridine (**1**, **3**), piperidine (**9**–**10**), and 2-N-methyl-pyrrolidine (**13**–**14**) linked at the nitrogen atom of the sulfonamide by a two-carbon chain. No correlation between activity and structure can be attributed to the aromatic moiety since the presence of phenyl, toluyl, and benzyl does not markedly alter the potency. Interestingly, all active molecules contain a two-methylene linker, except compound **3**, which has a pyridine ring linked to the sulfonamide function. The presence of a second heteroatom in the cycle, as in morpholine (compounds **15**–**17**), does not determine a variation in activity [36].

Consistent with the enzymatic assay (Figure 4), molecules **4**–**8** and **11**–**12** show no activity, although some of them have cellular activity, as reported in Table 1.

The replacement of the indole portion led to four compounds (**2**, **15**–**17**) with comparable inhibitory activity against aromatase and six compounds (**1**, **3**, **9**–**10**, **13**–**14**) with a ten times higher activity than the best indole sulfonamides from which the new compounds were derived (IC_50_ values between 0.16 and 0.75 μM) [26].

#### 2.3.2. Cell Viability and Cellular IC_50_

Considering the interesting results obtained with the enzymatic assay, the cell viability was assessed, and the cellular IC_50_ values were calculated by means of an MTT (3-(4,5-Dimethylthiazol-2-yl)-2,5-Diphenyltetrazolium Bromide) assay on a human breast cancer (MCF7) cell line, expressing the aromatase enzyme, and a mouse fibroblast cell line (NIH3T3), used as a healthy model, in the concentration range of 10 mM–1 nM for 24 h [37,38]. The cellular IC_50_ values calculated for each cell line are reported in Table 1. Most compounds exhibited favorable cellular IC_50_ values, particularly the most active compounds in the enzymatic assay. In this cellular assay, the IC_50_ values ranged between 2.67 and 43.61 μM for the MCF7 cell line, better than those for the NIH3T3 cell line (IC_50_ range: 29.62–64.30 μM).

Taking into account the cellular IC_50_ values of the most promising compounds in the enzymatic assay (**1**, **3**, **9**–**10**, **13**–**14**), the MTT data show that although these compounds display some activity against the healthy line (fibroblasts), they are much more active against MCF7 expressing the aromatase enzyme, confirming that this enzyme is involved in the anti-tumor action of the compounds.

**Figure 5:**
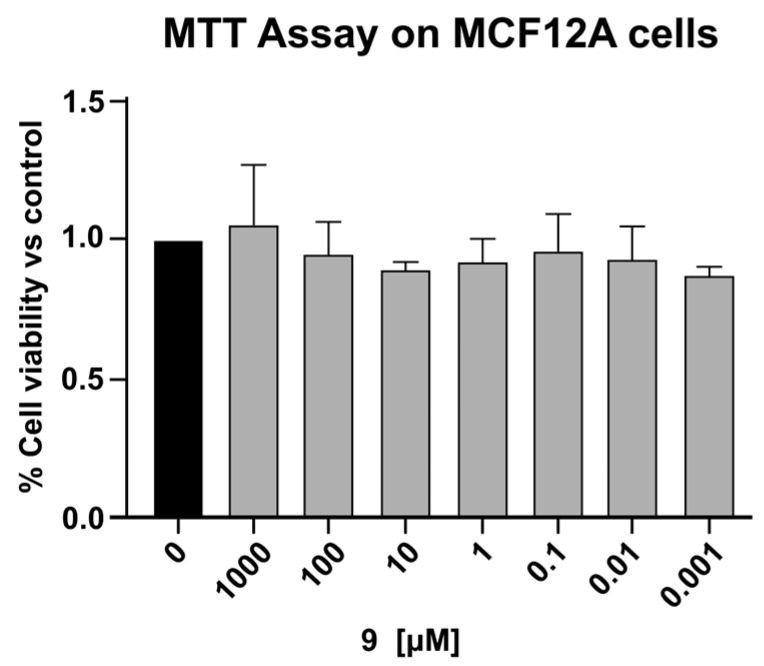
The percentage of non-tumorigenic MCF12A mammary cell viability with the most active compound **9**.

To investigate this aspect further, it was decided to also evaluate the cell viability using compound **9**, which was the most active in the previous assays, in a non-tumorigenic MCF12A mammary cell line using an MTT assay in the concentration range of 1 mM–1 nM for 24 h. As shown in Figure 5, this compound had no effects on MCF12A cell viability, revealing a certain degree of selectivity between tumorigenic and non-tumorigenic breast cells. This fact is of crucial importance to the effective and safe treatment of cancer since selectivity refers to the ability of a drug to target cancer cells while minimizing the harm to healthy cells. This targeted approach has the potential to yield more efficacious therapies with a reduced incidence of adverse effects, thereby enhancing patient outcomes.

### 2.4. The Computational Study

#### 2.4.1. The Molecular Docking Analysis of the Compounds

A molecular docking study was conducted on the aromatase enzyme (PDB: 3EQM) to identify the potential ligand binding mode using Glide [39,40]. To improve the accuracy of the docking, a QM-Polarized Ligand Docking (QPLD) protocol was employed [41,42]. The robustness of the docking protocol is demonstrated by the geometry of the crystallographic ligand (androstenedione) reproduced, with an RMSD of 0.3760 Å.

Among all studied sulfonamide compounds, those exhibiting the most promising activity (**1**, **3**, **9**–**10**, **13**–**14**) demonstrated robust molecular interactions within the active site, including those amino acids (Phe134, Trp224, Val370, Val373, and Met374) that interact with the natural ligand androstenedione. Furthermore, the cofactor heme group (HEM) plays a pivotal role in the binding of the phenyl or heterocyclic moieties of the new compounds. Table 2 provides a summary of the interactions between the most active compounds and aromatase.

All compounds are similarly located into the active site, probably because of the length of the chain between the sulfonamide and the heterocycle. In particular, the heterocycle is in front of the HEM, interacting via a π-π bond or a salt bridge; the oxygen from the sulfonamide forms an H-bond with Met374; and the phenyl or benzyl ring sits towards the access channel of the enzyme.

**Figure 6:**
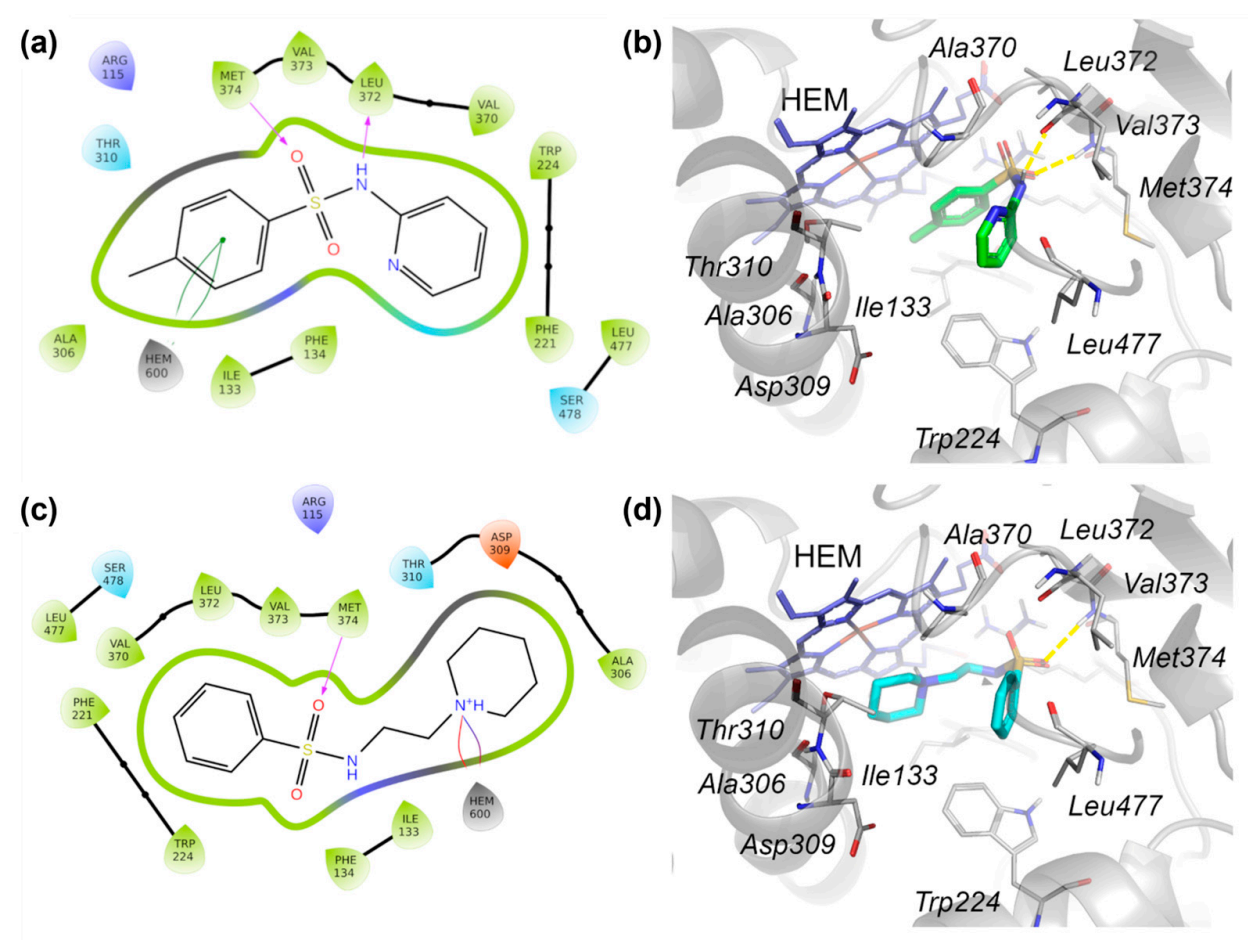
Two-dimensional and three-dimensional depictions of the predicted binding mode of compounds **3** (panels (**a**,**b**)) and **9** (panels (**c**,**d**)) with the active site of the aromatase enzyme (PDB ID 3EQM). The enzyme is represented by a cartoon, while the ligands are shown as sticks. The yellow dotted lines denote H-bonds.

As an example of the docking pose adopted by compounds **1**, **9**–**10**, and **13**–**14**, 2D and 3D depictions of the best docked pose of compound **9** are shown in Figure 6c,d. Derivative **3** demonstrated a comparable IC_50_ value in the enzymatic assay yet exhibited antithetical interactions compared to the others for the phenyl ring in front of the heme group, the oxygen from the sulfonamide group interacting with the nitrogen atom of the backbone of Met374, and the nitrogen atom from the sulfonamide with the backbone of Leu372 (Figure 6a,b). This result could be due to the absence of a chain linker in compound **3**, which may be the reason for it having the lowest docking score value.

To validate the stability of the interactions between **3** and **9** in the aromatase active site, a 100 ns molecular dynamic simulation was performed.

**Figure 7:**
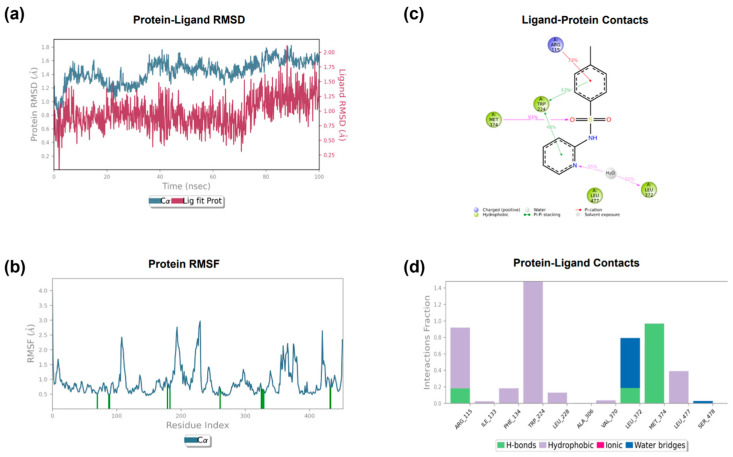
(**a**) Root mean square deviation (RMSD); (**b**) root mean squared fluctuation (RMSF); (**c**,**d**) contact between protein and ligand **3** during MD simulation.

**Figure 8:**
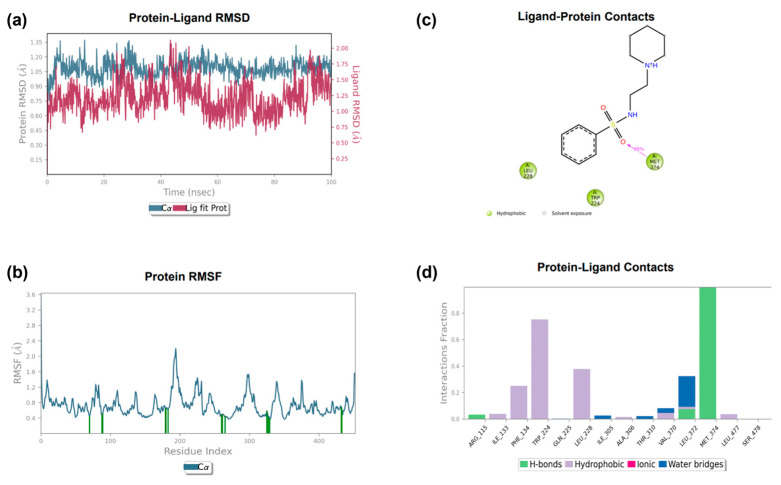
(**a**) Root mean square deviation (RMSD); (**b**) root mean squared fluctuation (RMSF); (**c**,**d**) contact between protein and ligand **9** during MD simulation.

The root mean square deviation (RMSD) of the ligands indicates that they are subjected to minimal movement, with a maximum displacement that does not reach 2.0 Å. The RMSD of the protein reaches a maximum of 1.8 Å for compound **3** and 1.35 Å for compound **9** (Figure 7a and Figure 8a, respectively), indicating the stability of the protein and generally stable protein–ligand complexes. The analysis of the protein RMSF graph confirms that the ligands stabilize the interacting residues (Figure 7b and Figure 8b, respectively), as highlighted by the minimal fluctuation in the binding residues.

The most relevant ligand–protein contacts are the π-π stacking between the benzene ring of **3** and Trp224 and the stable H-bond between Met374 and the sulfonamide group in **3** and **9** (Figure 7c,d and Figure 8c,d, respectively). The molecular dynamics analysis over 100 ns demonstrated that compounds **3** and **9** remained confined within the protein pocket without significant conformational changes.

#### 2.4.2. The Pharmacokinetic Profile

Suitable absorption characteristics and distribution patterns in a compound are indicative of its favorable pharmacokinetic profile, enabling it to reach the intended target site. The goal is to attain a clinical candidate that achieves a concentration–time profile in the body that is adequate for the desired efficacy and safety profile.

In order to evaluate the optimization of the absorption, distribution, metabolism, and excretion (ADME) parameters and the drug-like properties of the compounds, the QikProp tool was employed, using the default parameters [39]. This tool facilitates an evaluation of a wide array of pharmaceutical properties, with the most representative compounds presented in Table 3.

The most active compounds exhibited QPlogPo/w values ranging from 1.33 to 2.26, indicating low lipophilicity. Polar surface area (PSA) is a significant descriptor, correlating well with the molecular transport through membranes, and all compounds displayed PSA values within the acceptable range of 52.98–61.48 Å. Furthermore, the molecular weights and hydrogen bond donors/acceptors were also found to be within acceptable limits; this means that these properties fell within ranges that are considered favorable for the desired outcome, such as good oral bioavailability or target binding affinity. Noteworthy, all compounds were found to be non-violators of Lipinski’s rule of five [43,44] and exhibited high oral absorption. However, it was observed that only compound **3** displayed concerns regarding the blockage of the HERG K+ channel. This channel is of pivotal importance in the process of drug discovery, given its role in cardiac repolarization and its susceptibility to drug-induced QT prolongation, a potential cause of fatal heart arrhythmias. While hERG is often considered to be an “antitarget” (a channel that, when inhibited by a drug, leads to adverse effects), it is also being explored as a potential target in some therapeutic areas, particularly oncology. To mitigate the risk of investing resources into a drug candidate that fails preclinical safety studies due to QT prolongation, it is imperative to screen compounds for their activity against hERG channels during the early lead optimization process.

### 2.5. Searching for a Second Target Related to Breast Cancer

#### 2.5.1. Database Construction

In order to identify a further possible biological target implicated in breast cancer, we matched targets predicted by the target fishing technique to those expressed in breast cancer cells.

The online database SwissTargetPrediction (http://swisstargetprediction.ch/, accessed on 15 May 2025) was used to identify potential target genes of compounds **1**, **3**, **9**–**10**, and **13**–**14**, which were found to be the most active in aromatase inhibition. In the supporting information, Table S1 shows all possible targets identified for each individual compound.

The keyword “breast cancer” was used to search the GeneCards database (https://www.genecards.org/, accessed on 15 May 2025) for disease-related genes, and a total of 740 genes were identified in the “protein coding” category.

The breast-cancer-related genes were linked with the potential target genes of the sulfonamide compounds to identify intersections between the two sets, defining them as secondary potential targets for treating breast cancer, in addition to the aromatase enzyme. The intersection of the target genes with the breast-cancer-related genes for each compound is shown in Table 4.

As shown in Table 4, disregarding the CYP19A1 gene, the only target present for more than one compound is Protein Tyrosine Kinase 2 Beta (PTK2B), which has been identified as a target for compounds **1** and **3**.

PTK2B is a cytoplasmatic, non-receptor tyrosine kinase that plays a multifaceted and critical role in the progression, invasion, and metastasis of BC, as well as its resistance to therapy. PYK2 integrates signals from various cell surface receptors, including growth factor receptors such as epidermal growth factor receptor (EGFR) and HER2, as well as cytokine receptors. This integration activates downstream signaling pathways, such as STAT3 and MAPK, which drive aggressive tumor behaviors. Activation of PYK2 leads to increased transcription of MMP9, a matrix-degrading enzyme involved in tissue remodeling. This, in turn, promotes the spreading, migration, and invasion of cancer cells, contributing to a more malignant phenotype. In fact, elevated PYK2 activity is strongly associated with a higher tumor grade, lymph node metastasis, poor clinical outcomes, and resistance to targeted therapies [45,46,47].

Another interesting and extensively studied target in tumors is undoubtedly epidermal growth factor receptor (EGFR), which resulted in the prediction of sulfonamide **9**. EGFR plays a significant role in breast cancer, particularly in aggressive subtypes and in mediating resistance to therapy. EGFR is overexpressed in a considerable proportion of breast cancers, especially in triple-negative breast cancer (TNBC) and ER/PR-negative tumors. Elevated levels of EGFR expression are associated with a poor prognosis, a higher tumor grade, increased metastasis, and reduced survival [48,49,50,51].

EGFR overexpression is also associated with resistance to endocrine therapy (e.g., tamoxifen) and anti-HER2 therapy, particularly in ER-positive and HER2-positive subtypes. Targeting EGFR may help overcome resistance in these settings [52,53].

#### 2.5.2. Molecular Docking

A molecular docking study was used to identify the molecular interactions between the novel synthesized sulfonamides and the target proteins. The 3D structures of PTK2B and EGFR were retrieved from Protein Data Bank (PDB IDs 3FZS and 2RGP, respectively). The docking protocol was validated by the re-docking of the cognate ligand, obtaining RMSD values of 0.957 Å and 1.202 Å for PTK2B and EGFR, respectively.

**Figure 9:**
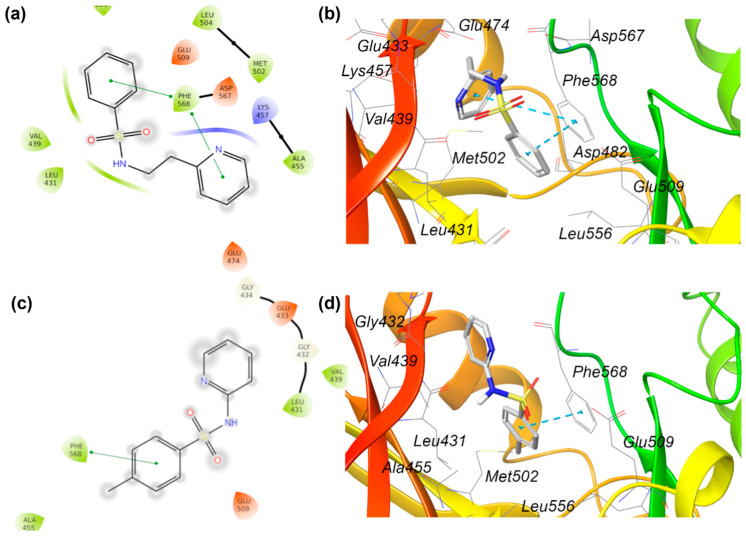
Two-dimensional and three-dimensional depictions of the predicted binding modes of compounds **1** (panels (**a**,**b**)) and **3** (panels (**c**,**d**)) in the active site of PTK2B (PDB ID: 3FZS). The enzyme is represented by a cartoon, while the ligands are shown as sticks. A blue line in dots represents a π-π interaction.

In the case of PTK2B, compounds **1** and **3** obtain the best docking score (−6.842 and −6.736 kcal mol^−1^, respectively). In Figure 9, the 3D and 2D interactions of the best docked poses are represented.

The fundamental interaction between compounds **1**–**3** and the target is a π-π interaction with Phe568, which is a key residue in the active site. The presence of the two-carbon chain linker in compound **1** enables Phe568 to interact with both aromatic rings, which is not possible for compound **3**.

Given the minute structure of the compounds under investigation and the large interaction area, which extends into the characteristic bilobed structure of this kinase, it can be hypothesized that they can be regarded as fragments to be enlarged or combined with other fragments to interact with the distal portion of the site.

**Figure 10:**
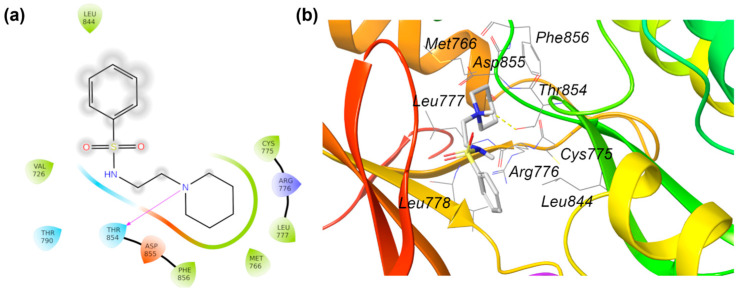
Two-dimensional and three-dimensional depictions of the predicted binding modes of compound **9** (panels (**a**,**b**)) with the active site of EGFR (PDB ID: 2RGP). The enzyme is represented by a cartoon, while the ligands are shown as sticks. A yellow dotted line represents a H-bond.

In the case of EGFR, compound **9** emerged as the best docked compound among the others, with a docking score of −7.936 kcal mol^−1^. As represented in Figure 10, there is a fundamental H-bond between the piperidinium hydrogen and the oxygen from the Thr854 residue, while the benzyl sulfonamide fits well with the entrance of EGFR’s active site.

In light of the in vitro activity of the most active compounds and the docking results obtained for aromatase, EGFR, and PTK2B, further rational design studies will be conducted in the near future.

## 3. Materials and Methods

### 3.1. Chemistry

All of the chemicals and solvents used in the synthetic protocols and the biological assay were sourced from commercial suppliers and used as received.

The progress of the reactions was monitored via thin-layer chromatography (TLC) on silica-gel-coated 60 TLC F254 Merck plates under UV light visualization. Purification of the synthetic mixture was carried out using flash chromatography on Merck silica gel 60. The uncorrected melting points (°C) were determined using a Buchi apparatus. The proton (^1^H) and carbon (^13^C) NMR spectra were recorded on a Varian instrument 300 MHz spectrometer using deuterated methanol or dichloromethane as the solvent and tetramethylsilane (TMS) as an internal reference. Elemental analyses for C, H, and N were recorded on a Perkin-Elmer 240 B microanalyzer, and the analytical results were within ± 0.4% of the theoretical values for all compounds. The purity of all compounds was over 98%.

#### 3.1.1. The General Procedure for the Synthesis of Compounds 1–2 and 7–17 (Method A)

A solution of proper sulfonyl chloride (1.5 eq.) in dry chloroform (3 mL/mmol) was added dropwise in a nitrogen atmosphere to a stirred solution of the appropriate amine (1 eq.) and Et_3_N (3 eq.) at 0 °C. The reaction mixture was initially stirred at 0 °C for 2 h, followed by an additional 21–24 h at room temperature. The resulting residue was added to water (15 mL) and extracted three times using dichloromethane (3 × 15 mL). The combined organic phases were dried over anhydrous sodium sulfate (Na_2_SO_4_), filtered, and evaporated under a reduced pressure to yield the crude products. These were purified either through column chromatography on silica gel, employing various mixtures of the eluents, or through recrystallization from ethanol.

N-(2-(pyridin-2-yl)ethyl)benzenesulfonamide (1): Thin needle crystals (silica gel, dichloromethane:methanol, 9.5:0.5), 58.0% yield (249.52 mg); m.p. 99.0–100.8 °C [54]; ^1^H NMR (CDCl_3_) δ 2.91 (t, 2 H, C*H*_2_, J = 6.6 Hz), 3.35 (q, 2 H, C*H*_2_, J_1-2_ = 6.0, J_2-3_ = 6.3 Hz), 6.20 (s, broad, 1 H, N*H*), 7.04 (d, 1 H, C*H*_Ar_, J = 8.4 Hz), 7.10 (q, 1 H, C*H*_Ar_, J_1-2_ = 4.8, J_2-3_ = 2.7 Hz), 7.42–7.57 (m, 4 H, C*H*_Ar_), 7.82 (d, 2 H, C*H*_Ar_, J = 8.4 Hz), 8.43 (d, 1 H, C*H*_Ar_, J = 4.5 Hz); ^13^C- NMR (CDCl_3_) δ 36.1, 42.2, 121.7, 123.4, 126.9, 128.9, 132.3, 136.6, 140.2, 149.0, 158.8.

4-methyl-N-(2-(pyridin-2-yl)ethyl)benzenesulfonamide (2): Thin needle crystals (silica gel, dichloromethane:methanol, 9.5:0.5), 56.8% yield (257.43 mg); m.p. 119.7–120.8 °C; ^1^H-NMR (C*D*_3_O*D*) δ 2.40 (s, 3 H, C*H*_3_), 2.89 (t, 2 H, C*H*_2_, J = 14.7 Hz), 3.20 (t, 2 H, C*H*_2_, J = 14.1 Hz), 7.23 (d, 2 H, C*H*_Ar_, J = 7.5 Hz), 7.33 (d, 2 H, C*H*_Ar_, J = 8.4 Hz), 7.67 (d, 3 H, C*H*_Ar_, J = 8.1 Hz), 8.38 (dt, 1 H, C*H*_Ar_, J_1-2_ = 1.8, J_2-3_ = 3.9 Hz); ^13^C-NMR (C*D*_3_O*D*) δ 19.9, 37.2, 42.3, 121.7, 123.8, 126.6, 129.2, 137.2, 137.4, 143.1, 148.3, 158.3.

N-(1-benzylpiperidin-4-yl)benzenesulfonamide (7): Light brown dense oil (silica gel, chloroform, 100%); 69.6% yield (241.49 mg); ^1^H NMR (C*D*_3_O*D*) δ 1.39–1.51 (m, 2 H, C*H*_2_), 1.72 (dd, 2 H, C*H*_2_, J_1-2_ = 1.5, J_2-3_ = 9.9 Hz), 2.01 (t, 3 H, C*H*_2_, J = 11.7 Hz), 2.69 (d, 2 H, C*H*_2_, J = 11.7 Hz), 3.16–3.20 (m, 1 H, C*H*), 4.71 (d, 1 H, C*H*,J = 6.6 Hz), 7.19–7.30 (m, 5 H, C*H*_Ar_), 7.45–7.57 (m, 3 H, C*H*_Ar_), 7.87 (dd, 2 H, C*H*_Ar_, J_1-2_ = 1.2, J_2-3_ = 6.6 Hz); ^13^C NMR (CD_3_OD) δ 32.9, 50.8, 51.7, 62.8, 126.8, 127.0, 128.2, 129.0, 132.4, 137.9, 141.3.

N-(1-benzylpiperidin-4-yl)-4-methylbenzenesulfonamide (8): Yellow dense oil (silica gel, dichloromethane:methanol, 9.5:0.5); 40.5% yield (146.48 mg); ^1^H NMR (C*D*_3_O*D*) δ 1.41–1.54 (m, 2 H, C*H*_2_), 1.62–168 (m, 2 H, C*H*_2_), 2.06 (t, 2 H, C*H*_2_, J = 11.7 Hz), 2.39 (s, 3 H, C*H*_3_), 2.74–2-79 (m, 2 H, C*H*_2_), 2.98–3.07 (m, 1 H, C*H*), 3.50 (s, 2 H, C*H*_2_), 7.23–7.30 (m, 5 H, C*H*_Ar_), 7.34 (d, 2 H, C*H*_Ar_, J = 8.1 Hz), 7.72 (d, 2 H, C*H*_Ar_, J = 8.7 Hz), ^13^C NMR (C*D*_3_O*D*) δ 20.0, 31.7, 50.1, 51.4, 62.1, 110.0, 126.5, 127.2, 127.9, 129.3, 129.3, 136.3, 138.8, 143,1.

N-(2-(piperidin-1-yl)ethyl)benzenesulfonamide (9): Yellow dense oil (silica gel, chloroform, 100%); 65.2% yield (272.97 mg); ^1^H NMR (C*D*Cl_3_) δ 1.30–1.43 (m, 6 H, C*H*_2_), 2.10 (t, 4 H, C*H*_2_, J = 4.8 Hz), 2.26 (t, 2 H, C*H*_2_, J = 5.7 Hz), 2.91 (t, 2 H, C*H*_2_, J = 6.0 Hz), 7.42–7.52 (m, 3 H, C*H*_Ar_), 7.81 (dd, 2 H, C*H*_Ar_, J_1-2_ = 1.2, J_2-3_ = 6.9 Hz); ^13^C NMR (C*D*Cl_3_) δ 24.0, 25.6, 39.1, 53.8, 56.1, 127.0, 129.0, 132.5, 139.5.

1-phenyl-N-(2-(piperidin-1-yl)ethyl)methanesulfonamide (10**):** A white granular solid (silica gel, chloroform:methanol, 99:1); 46.8% yield (206.17 mg); m.p. 64.9–65.3 °C; ^1^H NMR (C*D*Cl_3_) δ 1.34–1.37 (m, 2 H, C*H*_2_), 1.40–1.48 (m, 4 H, C*H*_2_), 2.24–2.25 (m, 4 H, C*H*_2_), 2.31 (t, 2 H, C*H*_2_, J = 6 Hz), 2.95 (t, 2 H, C*H*_2_, J = 5.7 Hz), 4.23 (s, 2 H, C*H*_2_,), 4.95 (s broad, NH), 7.30–7.33 (m, 3 H, C*H*_Ar_), 7.35–7.38 (m, 2 H, C*H*_Ar_); ^13^C-NMR (C*D*Cl_3_) δ29.2, 30.8, 45.0, 59.1, 62.5, 63.4, 133.5, 133.7, 134.7, 135.5.

N-(2-(piperidin-1-yl)ethyl)-1-(p-tolyl)methanesulfonamide (11): A dense oil (silica gel, dichloromethane:methanol, 9.5:0.5); 80.0% yield (352.43 mg); ^1^H NMR (C*D*Cl_3_) δ 1.38 (d, 2 H, C*H*_2_, J = 5.4 Hz), 1.49–1.57 (m, 4 H, C*H*_2_), 2.35 (s, 3 H, C*H*_3_), 2.38 (d, 4 H, C*H*_2_, J = 5.4 Hz), 2.51 (t, 2 H, C*H*_2_, J = 5.7 Hz), 2.99 (t, 2 H, C*H*_2_, J = 5.4 Hz), 7.23 (d, 2 H, C*H*_Ar_, J = 8.1 Hz), 7.70 (d, 2 H, C*H*_Ar_, J = 8.4 Hz); ^13^C NMR (C*D*Cl_3_) δ 21.4, 23.46, 24.9, 38.9, 53.8, 56.4, 127.0, 129.6, 136.6, 143.3.

4-methyl-N-(3-(pyrrolidin-1-yl)propyl)benzenesulfonamide (12): Brown dense oil (silica gel, dichloromethane:methanol, 9.5:0.5); 46.8% yield (206.17 mg); ^1^H NMR (C*D*_3_O*D*) δ 1.85–1.95 (m, 2 H, C*H*_2_); 2.47–2.09 (m, 4 H, C*H*_2_); 2.42 (s, 3 H, C*H*_3_); 2.93 (t, 2 H, C*H*_2_J = 6.3 Hz); 3.23 (t, 2 H, C*H*_2_, J = 6.0 Hz); 3.30–3.33 (m, 4 H, C*H*_2_); 4.49 (s, broad, NH); 7.39 (d, 2 H, C*H*_Ar_, J = 8.1 Hz); 7.73 (d, 2 H, C*H*_Ar_,J = 8.1 Hz); ^13^C NMR (C*D*_3_O*D*) δ 20.1, 22.6, 25.9, 39.7, 52.3, 53.8, 126.7, 129.5, 137.1, 143.5.

N-(2-(1-methylpyrrolidin-2-yl)ethyl)benzenesulfonamide (13): Brown dense oil (silica gel, dichloromethane:methanol, 9:1); 78.6% yield (326.96 mg); ^1^H NMR (C*D*_3_O*D*) δ 1.65–1.84 (m, 2 H, C*H*_2_), 1.96–2.21 (m, 3 H, C*H*_2_), 2.27–2.38 (m, 1 H, C*H*), 2.85 (s, 3 H, C*H*_3_), 2.88–3.06 (m, 2 H, C*H*_2_), 3.12–3.20 (m, 1 H, C*H*), 3.39 (s, broad, 1 H, C*H*), 3.63 (s, broad, 1 H, C*H*), 7.56–7.67 (m, 3 H, C*H*_Ar_), 7.86–7.91 (m, 2 H, C*H*_Ar_); ^13^C NMR (C*D*_3_O*D*) δ 21.1, 29.0, 30.2, 38.5, 39.6, 55.8, 66.68, 126.6, 129.1, 132.6, 139.9.

4-methyl-N-(2-(1-methylpyrrolidin-2-yl)ethyl)benzenesulfonamide (14): Dark white amorphous powder (silica gel, dichloromethane:methanol, 9.5:0.5); 84.33% yield (369.12 mg); 97.4–101.7 °C; ^1^H NMR (C*D*_3_O*D*) δ 1.66–1.84 (m, 2 H, C*H*_2_), 1.96–2.20 (m, 3 H, C*H*_2_ + C*H*), 2.27–2.37 (m, 1 H, C*H*), 2.40 (s, 3 H, C*H*_3_), 2.85 (s, 3 H, C*H*_3_), 2.89–3.04 (m, 2 H, C*H*_2_), 3.09–3.20 (m, 1 H, C*H*), 3.36–3.47 (m, 1 H, C*H*), 3.58–3.66 (m, 1 H, C*H*), 4.82 (s, broad, NH), 7.39 (d, 2 H, C*H*_Ar_, J = 8.1 Hz); 7.75 (d, 2 H, C*H*_Ar_, J = 8.7 Hz); ^13^C NMR (C*D*_3_O*D*) δ 20.2, 21.1, 28.9, 30.1, 38.3, 39.6, 55.7, 62.7, 66.5, 110.0, 126.7, 129.6, 137.0, 143.6.

N-(2-morpholinethyl)benzenesulfonamide (15): Brown dense oil (silica gel, dichloromethane:methanol, 9:1); 59.55% yield (247.92 mg); ^1^H-NMR (C*D*Cl_3_) δ 2.12 (t, 4 H, C*H*_2_, J = 4.8 Hz), 2.26 (t, 2 H, C*H*_2_, J = 5.3 Hz), 2.89 (t, 2 H, C*H*_2_, J = 6.6 Hz), 3.46 (t, 4 H, C*H*_2_, J = 4.8 Hz), 7.37–7.49 (m, 3 H, C*H*_Ar_), 7.78 (dd, 2 H, C*H*_Ar_, J = 1.2, 3.6 Hz); ^13^C-NMR (C*D*Cl_3_) δ 39.1, 52.8, 56.2, 66.5, 126.9, 129.0, 132.6, 139.59.

4-methyl-N-(2-morpholinethyl)benzenesulfonamide (16): Solid white needles (silica gel, dichloromethane:methanol, 9.5:0.5); 46.80% yield (204.95 mg); 110.9–111.2 °C; ^1^H-NMR (C*D*_3_O*D*) δ 2.35 (t, 2 H, C*H*_2_, J = 4.8 Hz), 2.38 (d, 2 H, C*H*_2,_ J = 7.2 Hz) 2.41 (s, 3 H, C*H*_3_), 2.97 (t, 2 H, C*H*_2_, J = 6 Hz), 3,29–3.30 (m, 2 H, C*H*_2_), 3.60 (t, 2 H, C*H*_2_, J = 3 Hz), 4.79 (broad, N*H*), 7.36 (d, 2 H, C*H*_Ar_, J = 9 Hz), 7.73 (dd, 2 H, C*H*_Ar_, J_1-2_ = 1.8 Hz, J_2-3_ = 4.5 Hz); ^13^C NMR (C*D*Cl_3_) δ 19.9, 39.4, 53.1, 57.1, 66.2, 126.6, 129.3, 137.4, 143.3.

N-(2-morpholinoethyl)-1-phenylmethanesulfonamide (17): Brown dense oil (silica gel, chloroform, 100%); 49.25% yield (215.68 mg); ^1^H-NMR (C*D*Cl_3_) δ 1.92–1.99 (m, 6 H, C*H*_2_), 2.59 (t, 2 H, C*H*_2_, J = 11.4 Hz), 3.18 (t, 4 H, C*H*_2_, J = 4.5 Hz), 3.71 (s, 2 H, C*H*_2_), 4.75 (s, broad, 1 H, N*H*), 6.93–7.01 (m, 5 H, C*H*_Ar_); ^13^C NMR (C*D*Cl_3_) δ 39.6, 53.1, 57.4, 58.4, 66.6, 128.6, 128.7, 129.6, 130.5.

#### 3.1.2. The General Procedure for the Synthesis of Compounds 3–6 (Method B)

A solution of proper sulfonyl chloride (1.5 eq.) in dry chloroform (3 mL/mmol) was added dropwise in a nitrogen atmosphere to a stirred solution of the appropriate amine (1 eq.) and pyridine (3 eq.) at 0 °C. The mixture was reacted at 0 °C for 2 h and for 24–48 h at room temperature. The residue was poured into water (15 mL) and extracted using dichloromethane (3 × 15 mL). The combined organic layers were dried (Na_2_SO_4_), filtered, and concentrated under a vacuum to provide the crude products, which were purified through column chromatography on silica gel using different mixtures of eluents or through crystallization from ethanol, as described below.

4-metyl-N-(pyridin-2-yl)benzenesulfonamide (3): An amorphous white crystal (crystal from ethanol), 62.0% yield (327.90 mg); m.p. 215.5–216.1; ^1^H NMR (C*D*Cl_3_) δ 2.38 (s, 3 H, C*H*_3_); 6.81 (t, 1 H, C*H*_Ar_, J = 7.2 Hz); 7.24 (d, 2 H, C*H*_Ar_, J = 8.1 Hz); 7.43 (d, 1 H, J = 8.7 Hz); 7.68 (t, 1 H, C*H*_Ar_, J = 1.8 Hz); 7.79 (d, 2 H, C*H*_Ar_, J =8.1 Hz); 8.32 (d, 1 H, C*H*_Ar_, J = 6.0 Hz); ^13^C NMR (C*D*Cl_3_) δ 21.4, 114.3, 114.9, 126.8, 129.5, 138.7, 141.0, 141.8, 142.8, 154.9.

N-(5-chloropyridin-2-yl)-4-methylbenzenesulfonamide (4): An amorphous white crystal (crystal from ethanol), 53.2% yield (234.65 mg); m.p. 173.2–175.4 °C; ^1^H NMR (C*D*Cl_3_) δ 2.38 (s, 3 H, C*H*_3_), 7.24 (d, 2 H, C*H*_Ar_, J = 4.2 Hz), 7.41 (d, 1 H, C*H*_Ar_, J = 8.4 Hz), 7.61 (dd, 1 H, C*H*_Ar_, J_1-2_ = 2.4 Hz, J_2-3_= 6.3 Hz), 7.70 (d, 2 H, C*H*_Ar_, J = 8.7 Hz), 8.42 (d, 1 H, C*H*_Ar_, J = 3.6 Hz); ^13^C NMR (C*D*Cl_3_) δ 21.5, 112.9, 126.9, 127.0, 129.8, 136.3, 138.8, 144.2, 147.3, 149.6.

N-(3,5-dichloropyridin-2-yl)-4-methylbenzenesulfonamide (5): An amorphous white crystal (silica gel, chloroform, 100%), 48.0% yield (187.26 mg); m.p. 146.1–147.1 °C; ^1^H NMR (C*D*_3_O*D*) δ 2.40 (s, 3 H, C*H*_3_), 7.34 (d, 2 H, C*H*_Ar_, J = 7.5 Hz), 7.85 (d, 1 H, C*H*_Ar_, J = 2.4 Hz), 7.92 (d, 2 H, C*H*_Ar_, J = 8.41 Hz), 8.06 (d, 1 H, C*H*_Ar_, J = 1.8 Hz); ^13^C NMR (C*D*_3_O*D*) δ 21.5, 117.9, 128.0, 128.1, 131.6, 137.9, 140.0, 137.9, 140.0, 145.9, 148.9, 151.4.

N-(3,5-dichloropyridin-2-yl)-1-phenylmethanesulfonamide (6): White powder (silica gel, chloroform, 100%), 35.8% yield (139.6 mg); m.p. 150.2–152.7 °C; ^1^H NMR (C*D*Cl_3_) δ 4.88 (s, 2 H, C*H*_2_), 7.04 (s, broad, N*H*), 7.31–7.36 (m, 5 H, C*H*_Ar_), 7.72 (d, 1 H, C*H*_Ar_, J = 1.2 Hz), 8.32 (d, 1 H, C*H*_Ar_, J = 1.2 Hz); ^13^C NMR (C*D*Cl_3_) δ 58.1, 117.9, 126.6, 128.0, 128.1, 128.1, 129.3, 130.3, 130.3, 148.9, 151.4.

### 3.2. The Biological Assay

#### 3.2.1. The Cell Culture

Adenocarcinomic human breast epithelial cells (MCF7, ATCC HTB-22), the mouse fibroblast cell line (NIH3T3, ATCC CRL-1658), and the MCF12A human non-tumorigenic mammary epithelial cell line were purchased from ATCC (Manassas, VA, USA).

MCF7 and NIH3T3 were grown in Dulbecco’s Modified Eagle Medium (DMEM, Sigma-Aldrich, USA); supplemented with 10% fetal bovine serum (Capricorn Scientific, Ebsdorfergrund, Germany) and 1% antibiotics (100 mg/mL of streptomycin and 100 units of penicillin, Sigma-Aldrich, Taufkirchen, Germany); and incubated until confluent at 37 °C in a humidified incubator with a 5% CO_2_ atmosphere.

MCF12A was maintained in DMEM/Ham’s F12 (1:1); supplemented with 5% horse serum, 20 ng/mL of human epidermal growth factor, 0.01 mg/mL of bovine insulin, 500 ng/mL of hydrocortisone, and 100 U/mL (50 µg/mL) of penicillin–streptomycin; and maintained at 37 °C in a humidified atmosphere of 5% CO_2_.

#### 3.2.2. The Aromatase Inhibition Assay

The in vitro aromatase inhibition assay was performed using a kit procedure (BioVision, Aromatase (CYP19A) Inhibitor Screening Kit (Fluorometric)) in accordance with the method previously reported by our group [55]. The compounds were dissolved in 2% dimethyl sulfoxide (DMSO) and added to the assay at 7 concentrations at least within the range of 10^−3^–10^−9^ M. Letrozole was used as the positive inhibition control at a concentration of 1 μM. The blank, the control, and all concentrations of the inhibitors were analyzed in quadruplicate. The results on the percentage of inhibition are displayed as the mean ± standard deviation (SD). Moreover, the IC_50_ values were calculated with the help of GraphPad ‘PRISM’ software (version 5.0) by using a dose–response curve created by plotting the percentage inhibition versus the log concentration.

#### 3.2.3. The Cytotoxicity Assay

The MTT test is a cytotoxicity assay that is used to identify living cells by the color change in the formazan salt formed [56]. The cytotoxicity assays were performed using the human MCF7, mouse fibroblast NIH3T3, and human non-tumorigenic mammary epithelial MCF12A cell lines in accordance with the MTT procedure previously reported [37,38].

The compounds were dissolved in dimethyl sulfoxide (DMSO, AppliChem, Darmstadt, Germany) and diluted to the required concentrations with fresh medium, while the solvent control was prepared with a medium containing 0.1% DMSO. The half-maximal inhibitory concentrations (IC_50_) of the compounds were determined by calculating the cell viability via the MTT assay. The cells were harvested and counted using an automated cell counter (LUNA II, Logos Biosystems, Gyeonggi, Republic of Korea). The cells were then seeded into 96-well plates at a density of 1 × 104 cells per well, after which point they were subjected to different concentrations of the compounds (100, 20, 4, 0.8, and 0.16 µM). Following a 24 h incubation period, the MTT solution was added to the wells to a final concentration of 5 mg/mL. The cells were incubated for a 3 h period, after which point the medium was removed and 100 µL of DMSO was added. The absorbances were measured at 540 nm using a MultiMode Plate Reader (BioTek, HTX Synergy, Winooski, Vermont, USA). The cell survival rates were expressed as the percentage of the DMSO (0.1%) solvent control, and the IC50 concentrations were calculated according to the result of the analysis.

### 3.3. The Computational Study

#### 3.3.1. Molecular Docking

The molecular modeling studies were performed using Schrödinger Life Science Suite 2023–1 [38]. The ligands were drawn as 2D structures from Maestro and prepared using LigPrep to generate the 3D geometries and find all possible tautomers and protonation states at a pH = 7.0 ± 0.4 with Epik [57,58]. The 3D X-ray structure of aromatase was retrieved from Protein Data Bank (PDB ID: 3EQM) [18] and was corrected, optimized, and minimized using the Protein Preparation workflow. The molecular docking analyses were performed using Glide software. An enclosing box grid was generated using the centroid of the crystallographic ligand’s center of mass. Qsite refinement was carried out using Glide SP docking to generate multiple poses, and the partial atomic charges in accurate mode were calculated for each pose of compounds **1**, **3**, **9**–**10**, and **13**–**14** bound with aromatase. The most energetically favorable ligand poses were re-docked with Glide SP using the charge sets for each ligand. The final selection was made according to the Glide scores of the poses. Re-docking of the cognate ligand was used to assess the validity of the protocol (RMSD: 0.5392Å).

Autodock Vina was used for molecular docking of the best active aromatase inhibitors to the predicted target proteins (PTK2B and EGFR) [59,60]. The 3D coordinates of the proteins were obtained from the PDB database (PDB IDs: 3FZS and 2RGP, respectively).

The grid was centered on each natural ligand, and the size was also determined with reference to the natural ligands. After docking, the interactions between the receptor and ligand molecules were examined using the free version of Maestro.

#### 3.3.2. Molecular Dynamics

Molecular dynamics simulations were carried out using Desmond, available in Schrödinger Suite 2023–4 [39]. Complexes of aromatase with the docked poses of compounds 3 and 9 were embedded into an orthorhombic box of TIP4P water molecules, resulting in systems of 54,171 and 54,158 atoms, respectively. In order to balance the system charge, three and four Cl ions were added to the complexes with **3** and **9**, respectively. Six relaxation stages were applied to the systems as the default protocol before the simulation. The systems were treated with the OPLS4 force field and a normal pressure–temperature (NPT) ensemble, with a Nose–Hoover thermostat set to 300 K and a Martyna–Tobias–Klein barostat set to a 1.01325 bar pressure. The simulation production phase lasted 100 ns, recording frames every 100 ps.

#### 3.3.3. ADME Parameters and Drug-like Properties

Physico-chemical and pharmacokinetic parameters were calculated using QikProp and applying the default parameters [39].

## 4. Conclusions

Given the role of estrogen in promoting the growth of ER-positive breast cancer, aromatase has become an important molecular target for the development of anticancer agents, and AIs have been approved by the FDA as first-line therapy. However, some unexpected obstacles have increasingly shown up, such as resistance to AI treatment and side effects. This evidence drives the need for a newer generation of inhibitors to overcome this resistance, alongside reducing toxicity.

In view of exploring the structural determinants of aromatase inhibition, we aimed to expand our previous knowledge obtained using homemade derivatives containing an indole nucleus. Here, we report the synthesis and biological evaluation of seventeen sulfonamide derivatives obtained by conjugating a phenyl or benzyl moiety with an aliphatic or aromantic heterocyclic nucleus. Most of the tested molecules inhibited aromatase at sub-micromolar concentrations, and 2-pyridine, piperidine, and 2-N-methyl-pyrrolidine stood out as the best heterocycles, exhibiting activity like that of letrozole. The newly synthesized compounds were tested for their anticancer properties against the human breast cancer cell line MCF7, the mouse fibroblast cell line NIH3T3, and the healthy breast cell line MCF12A. In this case, the IC_50_ values were also in the low micromolar range, revealing a certain degree of selectivity between tumorigenic and non-tumorigenic breast cancer cell lines.

Docking studies were conducted to verify the possible binding modes of the most active molecules, and these dynamics revealed stable ligand–protein complexes. A combination between 2-pyridine or piperidine and a two-methylene chain was found to be essential for aromatase and cellular inhibition. The combination of the target fishing approach with breast-cancer-associated genes enabled the identification of two possible targets (EGFR and PTK2B) that could serve as multi-target ligands.

The favorable replacement of the indole portion led to compounds **3** and **9,** which outperformed the parent compound in terms of their potency and selectivity, demonstrating significant therapeutic potential. This information will also be crucial for designing new selective and potent AIs.

## Figures and Tables

**Table 1:** Aromatase and cellular (MCF7 and NIH3T3) IC_50_ values of the new compounds and LTR and doxorubicin as reference drugs *.

	Aromatase IC_50_ * (µM)	MCF7 IC_50_ * (µM)	NIH3T3 IC_50_ * (µM)
**1**	0.060 ± 0.002	7.511 ± 0.296	46.910 ± 1.994
**2**	0.248 ± 0.010	15.410 ± 0.356	51.368 ± 1.743
**3**	0.035 ± 0.001	2.679 ± 0.104	64.306 ± 1.832
**4**	>100	>100	>100
**5**	>100	>100	76.582 ± 1.126
**6**	>100	25.311 ± 0.945	>100
**7**	>1000	28.469 ± 0.514	44.275 ± 1.055
**8**	>1000	>100	>100
**9**	0.032 ± 0.001	3.288 ± 0.119	58.241 ± 0.952
**10**	0.052 ± 0.002	6.201 ± 0.258	30.856 ± 1.028
**11**	>100	43.617 ± 1.863	29.629 ± 0.812
**12**	>1000	32.062 ± 1.166	>100
**13**	0.046 ± 0.002	8.062 ± 0.319	49.630 ± 1.726
**14**	0.051 ± 0.001	10.505 ± 0.477	56.057 ± 1.930
**15**	0.337 ± 0.012	12.386 ± 0.417	39.276 ± 0.995
**16**	0.160 ± 0.006	19.579 ± 0.678	50.943 ± 1.510
**17**	0.206 ± 0.009	14.613 ± 0.522	43.057 ± 1.029
**LTR**	0.026 ± 0.001	/	/
**Doxorubicin**	/	1.940 ± 0.084	>100

* The test results were expressed as the means of quartet assays.

**Table 2:** Binding interactions and docking scores of the most active sulfonamide derivatives (**1**, **3**, **9**–**10**, **13**–**14**) with aromatase’s active site residues. All compounds interact with Phe134, Trp224, Val370, Val373, and Met374.

Cmp	H-Bond	π-π Bond	Salt Bridge	Docking Score (kcal/mol)
**1**	SO-Met374	Py-HEM	/	−7.423
**3**	SO-Met374 NH-Leu372	Ph-HEM	/	−7.280
**9**	SO-Met374	Ph-Trp224	NH-HEM	−7.864
**10**	SO-Met374	Ph-Trp224	NH-HEM	−8.147
**13**	SO-Met374	/	NH-HEM	−7.608
**14**	SO-Met374	/	NH-HEM	−7.570

**Table 3:** The physicochemical and pharmacokinetic properties of the studied ligands (**1**, **3**, **9**–**10**, **13**–**14**).

cmp	MW	donorHB	accptHB	QPlogPo/w	PSA	CNS	QPlogHERG	QPPCaco	QPlogBB	QPPMDCK	QPlogKhsa	RuleOfFive	hOralAbs	% hOralAbs
**1**	262.33	1	5.5	1.56	61.48	−2	−4.51	1019.17	−0.59	505.91	−0.42	0.00	3.00	91.71
**3**	248.30	1	5.5	2.08	57.98	0	−5.19	1284.06	−0.45	661.61	−0.22	0.00	3.00	92.91
**9**	268.37	1	6.5	2.97	53.65	1	−4.22	362.05	−0.03	182.52	0.17	0.00	3.00	78.71
**10**	282.40	1	6.5	2.83	52.98	0	−4.18	500.69	0.03	259.12	−0.15	0.00	3.00	83.43
**13**	268.37	1	6.5	0.22	56.13	1	−4.46	311.97	−0.10	157.74	−0.79	0.00	3.00	77.74
**14**	282.40	1	6.5	1.39	56.14	1	−4.35	312.40	−0.12	158.05	−0.38	0.00	3.00	79.28

MW: molecular weight; donorHB: H-bond donor; acceptHB: H-bond acceptor; QPlogPo/w: predicted octanol/water partition coefficient (−2.0–6.5); PSA: Van der Waals surface area of polar nitrogen and oxygen atoms and carbonyl carbon atoms (7–200); CNS: predictive central nervous activity on a −2 (inactive) to +2 (active) scale; QPlogHERG: predicted IC_50_ value for the blockage of HERG K^+^ channels (a concern below −5); QPPCaco: predicted apparent Caco-2 cell permeability in nm/sec (Caco-2 cells are a model for the gut–blood barrier (500 = great)); QPlogBB: predicted brain/blood partition coefficient (−3.0–1.2); QPPMDCK: predicted apparent MDCK cell permeability in nm/sec (MDCK cells are considered to be a good mimic of the blood–brain barrier (500 = great)); QPlogKhsa: prediction of binding to human serum albumin (−1.5–1.5); RuleOfFive: number of violations of Lipinski’s rule of five; hOralAbs: human oral absorption (1, 2, or 3 for low, medium, or high); % hOralAbs: predicted human oral absorption on a 0 to 100% scale.

**Table 4:** Targets identified by the intersection between the BC-related protein coding genes and potential targets identified by target fishing.

	Gene Symbol	Description	Uniprot ID
**1**	MEN1	Menin 1	O00255
	CDK2	Cyclin-Dependent Kinase 2	P24941
	CYP17A1	Cytochrome P450 Family 17 Subfamily A Member 1	P05093
	IRAK4	Interleukin 1 Receptor-Associated Kinase 4	Q9NWZ3
	CYP19A1	Cytochrome P450 Family 19 Subfamily A Member 1	P11511
	PTK2B	Protein Tyrosine Kinase 2 Beta	Q14289
**3**	AKT1	AKT Serine/Threonine Kinase 1	P31749
	ERBB2	Erb-B2 Receptor Tyrosine Kinase 2	P04626
	PTK2B	Protein Tyrosine Kinase 2 Beta	Q14289
**9**	EGFR	Epidermal Growth Factor Receptor	P00533
**10**	TERT	Telomerase Reverse Transcriptase	O14746
	CASR	Calcium-Sensing Receptor	P41180
**13**	CYP19A1	Cytochrome P450 Family 19 Subfamily A Member 1	P11511
**14**	ABCC1	ATP-Binding Cassette Subfamily C Member 1	P33527
	CYP19A1	Cytochrome P450 Family 19 Subfamily A Member 1	P11511

## Data Availability

The original contributions presented in this study are included in the article/Supplementary Materials. Further inquiries can be directed to the corresponding authors.

## Supplementary Materials

The following supporting information can be downloaded at https://www.mdpi.com/article/10.3390/ph18081206/s1.

## Abbreviations

The following abbreviations are used in this manuscript:

AI	Aromatase Inhibitor
EGFR	Epidermal Growth Factor Receptor
ER	Estrogen Receptor
FSH	Follicle-Stimulating Hormone
HER2	Human Epidermal Growth Factor Receptor 2
LTR	Letrozole
PDB	Protein Data Bank
PR	Progesterone Receptor
PTK2B	Protein Tyrosine Kinase 2 Beta
