# Supplementary material for: Discovery and Evaluation of Novel Sulfonamide Derivatives Targeting Aromatase in ER+ Breast Cancer
Source: Pharmaceuticals (Basel). 2025 Aug 15;18(8):1206. doi: 10.3390/ph18081206 (PMC12389619; doi:10.3390/ph18081206)

Supplementary materials

# Discovery and Evaluation of Novel Sulfonamide Derivatives Targeting Aromatase in ER+ Breast Cancer

Barbara De Filippis[1], Mariangela Agamennone[1], Alessandra Ammazzalorso[1], Rosa Amoroso[1], Letizia Giampietro[1], Cristina Maccallini[1], Begüm Nurpelin Sağlık[2], Chiara De Simone[3], Maria Chiara Zuccarini[3], Zafer Asım Kaplancıklı[2], Marialuigia Fantacuzzi[1],*

[1] *Department of Pharmacy, "G. d'Annunzio" University, Chieti, Italy*

[2] *Department of Pharmaceutical Chemistry, Faculty of Pharmacy, Anadolu University, Eskişehir, Turkey*

[3] *Department of Medical, Oral and Biotechnological Sciences, University "G. D'Annunzio" of Chieti-Pescara, 66100 Chieti, Italy*

*Corresponding author: Marialuigia Fantacuzzi, Department of Pharmacy, "G. d'Annunzio" University, Via dei Vestini 31, 66100 Chieti, Italy; Ph. +39 0871 3554685; marialuigia.fantacuzzi@unich.it

## Index

| | | |
|---|---|---|
| **Table S1** | Physical and analytical data of all new synthesized compounds **1-17** | ***4*** |
| **Figure S1** | Identified target of compounds **1**, **3**, **9-10**, **13-14** by SwissTargetPrediction. | *4* |
| **Figure S2** | $^{1}$H-NMR spectra (C*D*Cl3) for *N-(2-(pyridin-2-yl)ethyl)benzenesulfonamide*, **1** | ***15*** |
| **Figure S3** | $^{13}$C-NMR spectra (C*D*Cl3) for *N-(2-(pyridin-2-yl)ethyl)benzenesulfonamide*, **1** | ***16*** |
| **Figure S4** | $^{1}$H-NMR spectra (C*D*3O*D*) for *4-methyl-N-(2-(pyridin-2-yl)ethyl)benzenesulfonamide*, **2** | ***17*** |
| **Figure S5** | $^{13}$C-NMR spectra (C*D*3O*D*) for *4-methyl-N-(2-(pyridin-2-yl)ethyl)benzenesulfonamide*, **2** | ***18*** |
| **Figure S6** | $^{1}$H-NMR spectra (C*D*Cl3) for *4-metyl-N-(pyridin-2-yl)benzenesulfonamide*, **3** | ***19*** |
| **Figure S7** | $^{13}$C-NMR spectra (C*D*Cl3) for *4-metyl-N-(pyridin-2-yl)benzenesulfonamide*, **3** | ***20*** |
| **Figure S8** | $^{1}$H-NMR spectra (C*D*Cl3) for *N-(5-chloropyridin-2-yl)-4-methylbenzenesulfonamide*, **4** | ***21*** |

| | | |
|---|---|---|
| **Figure S9** | $^{13}$C-NMR spectra (C*D*Cl$_3$) for *N-(5-chloropyridin-2-yl)-4-22methylbenzenesulfonamide*, **4** | *22* |
| **Figure S10** | $^{1}$H-NMR spectra (C*D*Cl$_3$) for *N-(3,5-dichloropyridin-2-yl)-4-methylbenzenesulfonamide*, **5** | *23* |
| **Figure S11** | $^{13}$C-NMR spectra (C*D*Cl$_3$) for *N-(3,5-dichloropyridin-2-yl)-4-methylbenzenesulfonamide*, **5** | *24* |
| **Figure S12** | $^{1}$H-NMR spectra (C*D*Cl$_3$) for *N-(3,5-dichloropyridin-2-yl)-1-phenylmethanesulfonamide*, **6** | *25* |
| **Figure S13** | $^{13}$C-NMR spectra (C*D*Cl$_3$) for *N-(3,5-dichloropyridin-2-yl)-1-phenylmethanesulfonamide*, **6** | *26* |
| **Figure S14** | $^{1}$H-NMR spectra (C*D*$_3$O*D*) for *N-(1-benzylpiperidin-4-yl)benzenesulfonamide*, **7** | *27* |
| **Figure S15** | $^{13}$C-NMR spectra (C*D*$_3$O*D*) for *N-(1-benzylpiperidin-4-yl)benzenesulfonamide*, **7** | *28* |
| **Figure S16** | $^{1}$H-NMR spectra (C*D*$_3$O*D*) for *N-(1-benzylpiperidin-4-yl)-4-methylbenzenesulfonamide***,** **8** | *29* |
| **Figure S17** | $^{13}$C-NMR spectra (C*D*$_3$O*D*) for *N-(1-benzylpiperidin-4-yl)-4-methylbenzenesulfonamide***,** **8** | *30* |
| **Figure S18** | $^{1}$H-NMR spectra (CDCl$_3$) for *N-(2-(piperidin-1-yl)ethyl)benzenesulfonamide,* **9** | *31* |
| **Figure S19** | $^{13}$C-NMR spectra (CDCl$_3$) for *N-(2-(piperidin-1-yl)ethyl)benzenesulfonamide,* **9** | *32* |
| **Figure S20** | $^{1}$H-NMR spectra (C*D*Cl$_3$) for *N-(2-(piperidin-1-yl)ethyl)-1-(p-tolyl)methanesulfonamide*, **10** | *33* |
| **Figure S21** | $^{13}$C-NMR spectra (C*D*Cl$_3$) for *N-(2-(piperidin-1-yl)ethyl)-1-(p-tolyl)methanesulfonamide*, **10** | *34* |
| **Figure S22** | $^{1}$H-NMR spectra (C*D*Cl$_3$) for *1-phenyl-N-(2-(piperidin-1-yl)ethyl)methanesulfonamide***,** **11** | *35* |
| **Figure S23** | : $^{13}$C-NMR spectra (C*D*Cl$_3$) for *1-phenyl-N-(2-(piperidin-1-yl)ethyl)methanesulfonamide***,** **11** | *36* |
| **Figure S24** | $^{1}$H-NMR spectra (C*D*$_3$O*D*) for *4-methyl-N-(3-(pyrrolidin-1-yl)propyl)benzenesulfonamide*, **12** | *37* |
| **Figure S25** | $^{13}$C-NMR spectra (C*D*$_3$O*D*) for *4-methyl-N-(3-(pyrrolidin-1-yl)propyl)benzenesulfonamide*, **12** | *38* |

| | | |
|---|---|---|
| **Figure S26** | $^{1}$H-NMR spectra (C*D*$_3$O*D*) for *N-(2-(1-methylpyrrolidin-2-yl)ethyl)benzenesulfonamide*, **13** | ***39*** |
| **Figure S27** | $^{13}$C-NMR spectra (C*D*$_3$O*D*) for *N-(2-(1-methylpyrrolidin-2-yl)ethyl)benzenesulfonamide*, **13** | ***40*** |
| **Figure S28** | $^{1}$H-NMR spectra (C*D*$_3$O*D*) for *4-methyl-N-(2-(1-methylpyrrolidin-2-yl)ethyl)benzenesulfonamide*, **14** | ***41*** |
| **Figure S29** | $^{13}$C-NMR spectra (C*D*$_3$O*D*) for *4-methyl-N-(2-(1-methylpyrrolidin-2-yl)ethyl)benzenesulfonamide*, **14** | ***42*** |
| **Figure S30** | $^{1}$H-NMR spectra (C*D*Cl$_3$) for *N-(2-morpholinoethyl)benzenesulfonamide*, **15** | **43** |
| **Figure S31** | $^{13}$C-NMR spectra (C*D*Cl$_3$) for *N-(2-morpholinoethyl)benzenesulfonamide*, **15** | ***44*** |
| **Figure S32** | $^{1}$H-NMR spectra (C*D*$_3$O*D*) for *4-methyl-N-(2-morpholinoethyl)benzenesulfonamide,* **16** | ***45*** |
| **Figure S33** | $^{13}$C-NMR spectra (C*D*$_3$O*D*) for *4-methyl-N-(2-morpholinoethyl)benzenesulfonamide,* **16** | ***46*** |
| **Figure S34** | $^{1}$H-NMR spectra (C*D*Cl$_3$) for *N-(2-morpholinoethyl)-1-phenylmethanesulfonamide,* **17** | ***47*** |
| **Figure S35** | $^{13}$C-NMR spectra (CDCl$_3$) for *N-(2-morpholinoethyl)-1-phenylmethanesulfonamide,* **17** | ***48*** |

**Table S1.** Physical and analytical data of all new synthesized compounds **1-17**.

| cmp | MF | MW | Physical aspect | m.p. (°C) | Calculated (found)% | | |
|---|---|---|---|---|---|---|---|
| | | | | | C | H | N |
| **1** | $C_{13}H_{14}N_2O_2S$ | 262.33 | Thin needle white crystals | 99.0-100.8 | 59.52 (59.32) | 5.38 (5.37) | 10.68 (10.65) |
| **2** | $C_{14}H_{16}N_2O_2S$ | 276.36 | Thin needle crystals | 119.7-120.8 | 60.85 (60.72) | 5.84 (5.83) | 10.14 (10.11) |
| **3** | $C_{12}H_{12}N_2O_2S$ | 248.30 | Amorphous white crystal | 215.5-216.1 | 58.05 (57.89) | 4.87 (4.86) | 11.28 (11.24) |
| **4** | $C_{12}H_{11}ClN_2O_2S$ | 282.75 | Amorphous white crystal | 173.2-175.4 | 50.97 (50.81) | 3.92 (3.92) | 9.91 (9.93) |
| **5** | $C_{12}H_{10}Cl_2N_2O_2S$ | 317.19 | Amorphous white crystal | 146.1-147.1 | 45.44 (45.34) | 3.18 (3.18) | 8.83 (8.80) |
| **6** | $C_{12}H_{10}Cl_2N_2O_2S$ | 317.19 | White powder | 150.2-152.7 | 45.44 (45.48) | 3.18 (3.19) | 8.83 (8.86) |
| **7** | $C_{18}H_{22}N_2O_2S$ | 330.45 | Light Brown oil dense | - | 65.42 (65.27) | 6.71 (6.69) | 8.48 (8.47) |
| **8** | $C_{19}H_{24}N_2O_2S$ | 344.47 | Yellow dense oil | - | 66.25 (66.04) | 7.02 (7.01) | 8.13 (8.11) |
| **9** | $C_{13}H_{20}N_2O_2S$ | 268.38 | Yellow dense oil | - | 58.18 (58.03) | 7.51 (7.50) | 10.44 (10.41) |
| **10** | $C_{14}H_{22}N_2O_2S$ | 282.40 | White granular solid | 64.9-65.3 | 59.54 (59.36) | 7.85 (7.84) | 9.92 (9.90) |
| **11** | $C_{14}H_{22}N_2O_2S$ | 282.40 | Orange oil dense | - | 59.54 (59.34) | 7.85 (7.84) | 9.92 (9.90) |
| **12** | $C_{14}H_{22}N_2O_2S$ | 282.40 | Brown dense oil | - | 59.54 (59.41) | 7.85 (7.85) | 9.92 (9.91) |
| **13** | $C_{13}H_{20}N_2O_2S$ | 268.38 | Brown dense oil | - | 58.18 (57.99) | 7.51 (7.49) | 10.44 (10.42) |
| **14** | $C_{14}H_{22}N_2O_2S$ | 282.40 | Dark white powder | 97.4-101.7 | 59.54 (59.32) | 7.85 (7.84) | 9.92 (9.90) |
| **15** | $C_{12}H_{18}N_2O_3S$ | 270.35 | Brown dense oil | - | 53.31 (53.15) | 6.71 (6.70) | 10.36 (10.33) |
| **16** | $C_{13}H_{20}N_2O_3S$ | 284.38 | Solid white needles | 110.9-111.2 | 54.91 (54.72) | 7.09 (7.07) | 9.85 (9.83) |
| **17** | $C_{13}H_{20}N_2O_3S$ | 284.38 | Brown dense oil | - | 54.91 (54.74) | 7.09 (7.08) | 9.85 (9.82) |

**Figure S1**: Identified target of compounds **1**, **3**, **9-10**, **13-14** by SwissTargetPrediction. Target and common names, target class, and the Swiss Target Prediction probability were reported.

| Target | Common name | Target Class | Prob* |
|---|---|---|---|
| ***Compound 1*** | | | |
| Complement C1r | C1R | Protease | 0.054 |
| Cytochrome P450 19A1 | CYP19A1 | Cytochrome P450 | 0.054 |
| Metabotropic glutamate receptor 5 | GRM5 | Family C G protein-coupled receptor | 0.054 |
| Phosphodiesterase 10A | PDE10A | Phosphodiesterase | 0.054 |
| Isocitrate dehydrogenase [NADP] cytoplasmic | IDH1 | Enzyme | 0.054 |
| GABA-A receptor; alpha-5/beta-3/gamma-2 | GABRB3 GABRG2 GABRA5 | Ligand-gated ion channel | 0.054 |
| PI3-kinase p110-delta/p85-alpha | PIK3CD PIK3R1 | Enzyme | 0.054 |

| | | | |
|---|---|---|---|
| Ribosomal protein S6 kinase 1 | RPS6KB1 | Kinase | 0.054 |
| Serine/threonine-protein kinase Aurora-A | AURKA | Kinase | 0.054 |
| Corticotropin releasing factor receptor 1 | CRHR1 | Family B G protein-coupled receptor | 0.054 |
| Monoamine oxidase A | MAOA | Oxidoreductase | 0.054 |
| Monoamine oxidase B | MAOB | Oxidoreductase | 0.054 |
| Tankyrase-2 | TNKS2 | Enzyme | 0.054 |
| Vascular endothelial growth factor receptor 1 (by homology) | FLT1 | Kinase | 0.054 |
| Dihydrofolate reductase | DHFR | Oxidoreductase | 0.054 |
| Trace amine-associated receptor 1 (by homology) | TAAR1 | Family A G protein-coupled receptor | 0.054 |
| Carbonic anhydrase II | CA2 | Lyase | 0.054 |
| Carbonic anhydrase VA | CA5A | Lyase | 0.054 |
| Kir3.1/Kir3.4 | KCNJ5 KCNJ3 | Voltage-gated ion channel | 0.054 |
| P2X purinoceptor 7 | P2RX7 | Ligand-gated ion channel | 0.054 |
| Alpha-2a adrenergic receptor | ADRA2A | Family A G protein-coupled receptor | 0.054 |
| Adrenergic receptor alpha-2 | ADRA2C | Family A G protein-coupled receptor | 0.054 |
| Anandamide amidohydrolase | FAAH | Enzyme | 0.054 |
| Leukocyte elastase | ELANE | Protease | 0.054 |
| Histamine H4 receptor | HRH4 | Family A G protein-coupled receptor | 0.054 |
| 26S proteosome | PSMB1 | Protease | 0.054 |
| Beta-chymotrypsin | CTRB1 | Protease | 0.054 |
| Muscarinic acetylcholine receptor M2 (by homology) | CHRM2 | Family A G protein-coupled receptor | 0.054 |
| Muscarinic acetylcholine receptor M1 (by homology) | CHRM1 | Family A G protein-coupled receptor | 0.054 |
| Muscarinic acetylcholine receptor M3 (by homology) | CHRM3 | Family A G protein-coupled receptor | 0.054 |
| Protein farnesyltransferase | FNTA FNTB | Enzyme | 0.054 |
| Cyclin-dependent kinase 1/cyclin B | CCNB3 CDK1 CCNB1 CCNB2 | Other cytosolic protein | 0.054 |
| Tyrosine-protein kinase JAK3 | JAK3 | Kinase | 0.054 |
| Serine/threonine protein phosphatase PP1-alpha catalytic subunit | PPP1CA | Phosphatase | 0.054 |
| Glycogen synthase kinase-3 beta | GSK3B | Kinase | 0.054 |
| Tyrosine-protein kinase JAK2 | JAK2 | Kinase | 0.054 |
| Tankyrase-1 | TNKS | Enzyme | 0.054 |
| Epoxide hydratase | EPHX2 | Protease | 0.054 |
| Adenosine A2a receptor | ADORA2A | Family A G protein-coupled receptor | 0.054 |
| Hormone sensitive lipase | LIPE | Enzyme | 0.054 |
| ATP-binding cassette sub-family G member 2 | ABCG2 | Primary active transporter | 0.054 |
| Tyrosine-protein kinase SYK | SYK | Kinase | 0.054 |
| Focal adhesion kinase 1 | PTK2 | Kinase | 0.054 |
| TGF-beta receptor type I | TGFBR1 | Kinase | 0.054 |
| Elongation of very long chain fatty acids protein 6 | ELOVL6 | Enzyme | 0.054 |
| Translocator protein (by homology) | TSPO | Membrane receptor | 0.054 |
| 3-phosphoinositide dependent protein kinase-1 | PDPK1 | Kinase | 0.054 |

| | | | |
|---|---|---|---|
| Alpha-ketoglutarate-dependent dioxygenase alkB homolog 3 | ALKBH3 | Enzyme | 0.054 |
| Sodium/potassium/calcium exchanger 6 mitochondrial | SLC8B1 | Electrochemical transporter | 0.054 |
| Proteinase-activated receptor 1 | F2R | Family A G protein-coupled receptor | 0.054 |
| Nitric-oxide synthase endothelial | NOS3 | Enzyme | 0.054 |
| Serine/threonine-protein kinase PIM1 | PIM1 | Kinase | 0.054 |
| Dopamine transporter (by homology) | SLC6A3 | Electrochemical transporter | 0.054 |
| Interleukin-1 receptor-associated kinase 4 | IRAK4 | Kinase | 0.054 |
| Cyclin-dependent kinase 2 | CDK2 | Kinase | 0.054 |
| Fructose-1 6-bisphosphatase | FBP1 | Enzyme | 0.054 |
| Huntingtin | HTT | Unclassified protein | 0.054 |
| Interferon-induced double-stranded RNA-activated protein kinase | EIF2AK2 | Kinase | 0.054 |
| Tyrosine-protein kinase receptor FLT3 | FLT3 | Kinase | 0.054 |
| Alpha-1d adrenergic receptor | ADRA1D | Family A G protein-coupled receptor | 0.054 |
| Vascular endothelial growth factor receptor 2 | KDR | Kinase | 0.054 |
| Serine/threonine-protein kinase 17B | STK17B | Kinase | 0.054 |
| Serine/threonine-protein kinase SRPK1 | SRPK1 | Kinase | 0.054 |
| Metabotropic glutamate receptor 2 (by homology) | GRM2 | Family C G protein-coupled receptor | 0.054 |
| Geranylgeranyl transferase type I | PGGT1B FNTA | Enzyme | 0.054 |
| MAP kinase p38 alpha | MAPK14 | Kinase | 0.054 |
| Glutaminyl-peptide cyclotransferase | QPCT | Enzyme | 0.054 |
| Epoxide hydrolase 1 | EPHX1 | Protease | 0.054 |
| Cytochrome P450 17A1 (by homology) | CYP17A1 | Cytochrome P450 | 0.054 |
| Protein tyrosine kinase 2 beta | PTK2B | Kinase | 0.054 |
| Phosphodiesterase 5A | PDE5A | Phosphodiesterase | 0.054 |
| GABA-A receptor; alpha-3/beta-3/gamma-2 | GABRB3 GABRA3 GABRG2 | Ligand-gated ion channel | 0.054 |
| GABA-A receptor; alpha-1/beta-3/gamma-2 | GABRB3 GABRG2 GABRA1 | Ligand-gated ion channel | 0.054 |
| GABA-A receptor; alpha-2/beta-3/gamma-2 | GABRA2 GABRB3 GABRG2 | Ligand-gated ion channel | 0.054 |
| Cyclooxygenase-1 | PTGS1 | Oxidoreductase | 0.054 |
| Death-associated protein kinase 3 | DAPK3 | Kinase | 0.054 |
| Melanin-concentrating hormone receptor 1 | MCHR1 | Family A G protein-coupled receptor | 0.054 |
| Vanilloid receptor | TRPV1 | Voltage-gated ion channel | 0.054 |
| Acyl-CoA desaturase | SCD | Enzyme | 0.054 |
| Menin | MEN1 | Unclassified protein | 0.054 |
| Voltage-gated L-type calcium channel alpha-1C subunit | CACNA1C | Voltage-gated ion channel | 0.054 |
| GABA-A receptor; alpha-6/beta-3/gamma-2 | GABRG2 GABRB3 GABRA6 | Ligand-gated ion channel | 0.054 |
| GABA A receptor alpha-4/beta-3/gamma-2 | GABRB3 GABRA4 GABRG2 | Ligand-gated ion channel | 0.054 |
| c-Jun N-terminal kinase 3 | MAPK10 | Kinase | 0.054 |
| Cyclin-dependent kinase 1 | CDK1 | Kinase | 0.054 |
| Metabotropic glutamate receptor 1 | GRM1 | Family C G protein-coupled receptor | 0.054 |

| | | | |
|---|---|---|---|
| Bile acid receptor FXR | NR1H4 | Nuclear receptor | 0.054 |
| Puromycin-sensitive aminopeptidase | NPEPPS | Protease | 0.054 |
| Cytochrome P450 2C9 | CYP2C9 | Cytochrome P450 | 0.054 |
| LXR-beta | NR1H2 | Nuclear receptor | 0.054 |
| N-acylsphingosine-amidohydrolase (by homology) | NAAA | Enzyme | 0.054 |
| Butyrylcholinesterase | BCHE | Hydrolase | 0.054 |
| Macrophage migration inhibitory factor | MIF | Enzyme | 0.054 |
| Metabotropic glutamate receptor 4 | GRM4 | Family C G protein-coupled receptor | 0.054 |
| MAP kinase p38 beta | MAPK11 | Kinase | 0.054 |
| Galanin receptor 3 | GALR3 | Family A G protein-coupled receptor | 0.054 |
| Fatty acid desaturase 1 | FADS1 | Enzyme | 0.054 |
| Macrophage colony stimulating factor receptor | CSF1R | Kinase | 0.054 |
| Phosphodiesterase 3 | PDE3A | Phosphodiesterase | 0.054 |
| ***Compound 3*** | | | |
| 11-beta-hydroxysteroid dehydrogenase 1 | HSD11B1 | Enzyme | 0.103 |
| Endothelin receptor ET-A | EDNRA | Family A G protein-coupled receptor | 0.058 |
| Protein tyrosine kinase 2 beta | PTK2B | Kinase | 0.049 |
| Cyclooxygenase-2 | PTGS2 | Oxidoreductase | 0.049 |
| C-C chemokine receptor type 1 | CCR1 | Family A G protein-coupled receptor | 0.049 |
| C-C chemokine receptor type 4 | CCR4 | Family A G protein-coupled receptor | 0.049 |
| C-C chemokine receptor type 2 | CCR2 | Family A G protein-coupled receptor | 0.049 |
| Peptide N-myristoyltransferase 1 | NMT1 | Enzyme | 0.049 |
| Bromodomain-containing protein 4 | BRD4 | Reader | 0.049 |
| Carbonic anhydrase II | CA2 | Lyase | 0.049 |
| Receptor protein-tyrosine kinase erbB-2 | ERBB2 | Kinase | 0.049 |
| Bromodomain-containing protein 3 | BRD3 | Reader | 0.049 |
| Toll-like receptor 4 | TLR4 | Toll-like and Il-1 receptors | 0.049 |
| Cholecystokinin B receptor | CCKBR | Family A G protein-coupled receptor | 0.049 |
| Bromodomain-containing protein 7 | BRD7 | Reader | 0.049 |
| Adenosine A2a receptor | ADORA2A | Family A G protein-coupled receptor | 0.049 |
| Carbonic anhydrase I | CA1 | Lyase | 0.049 |
| Carbonic anhydrase XII | CA12 | Lyase | 0.049 |
| Carbonic anhydrase IX | CA9 | Lyase | 0.049 |
| Peroxisome proliferator-activated receptor alpha | PPARA | Nuclear receptor | 0.049 |
| Coagulation factor XIII | F13A1 | Aminoacyltransferase | 0.049 |
| c-Jun N-terminal kinase 3 | MAPK10 | Kinase | 0.049 |
| Bromodomain-containing protein 2 | BRD2 | Reader | 0.049 |
| fructose-2 6-bisphosphatase 3/4_UNCURATED | PFKFB4 PFKFB3 | Enzyme | 0.049 |
| Serine/threonine-protein kinase AKT | AKT1 | Kinase | 0.049 |
| Monoamine oxidase B | MAOB | Oxidoreductase | 0.049 |
| Poly [ADP-ribose] polymerase-1 | PARP1 | Enzyme | 0.049 |
| ***Compound 9*** | | | |
| Sigma opioid receptor | SIGMAR1 | Membrane receptor | 0.199 |
| Serotonin 7 (5-HT7) receptor | HTR7 | Family A G protein-coupled receptor | 0.063 |

| | | | |
|---|---|---|---|
| Norepinephrine transporter | SLC6A2 | Electrochemical transporter | 0.054 |
| Serotonin transporter | SLC6A4 | Electrochemical transporter | 0.054 |
| HERG | KCNH2 | Voltage-gated ion channel | 0.054 |
| Serotonin 2a (5-HT2a) receptor | HTR2A | Family A G protein-coupled receptor | 0.054 |
| Serotonin 2c (5-HT2c) receptor | HTR2C | Family A G protein-coupled receptor | 0.054 |
| 11-beta-hydroxysteroid dehydrogenase 1 | HSD11B1 | Enzyme | 0.054 |
| Cathepsin (B and K) | CTSB | Protease | 0.054 |
| Transient receptor potential cation channel subfamily V member 4 | TRPV4 | Voltage-gated ion channel | 0.054 |
| Calpain 1 | CAPN1 | Protease | 0.054 |
| Dopamine D2 receptor | DRD2 | Family A G protein-coupled receptor | 0.054 |
| Dopamine D3 receptor | DRD3 | Family A G protein-coupled receptor | 0.054 |
| Acetylcholinesterase | ACHE | Hydrolase | 0.054 |
| Polyadenylate-binding protein 1 | PABPC1 | Unclassified protein | 0.054 |
| FK506-binding protein 1A | FKBP1A | Isomerase | 0.054 |
| Serotonin 1a (5-HT1a) receptor | HTR1A | Family A G protein-coupled receptor | 0.054 |
| Serotonin 1b (5-HT1b) receptor | HTR1B | Family A G protein-coupled receptor | 0.054 |
| Serotonin 1d (5-HT1d) receptor | HTR1D | Family A G protein-coupled receptor | 0.054 |
| Dopamine transporter | SLC6A3 | Electrochemical transporter | 0.054 |
| Phosphodiesterase 2A | PDE2A | Phosphodiesterase | 0.054 |
| Phosphodiesterase 10A (by homology) | PDE10A | Phosphodiesterase | 0.054 |
| Prolyl endopeptidase | PREP | Protease | 0.054 |
| Monoamine oxidase B | MAOB | Oxidoreductase | 0.054 |
| Lysine-specific histone demethylase 1 | KDM1A | Eraser | 0.054 |
| Cannabinoid receptor 1 | CNR1 | Family A G protein-coupled receptor | 0.054 |
| Toll-like receptor (TLR7/TLR9) | TLR9 | Toll-like and Il-1 receptors | 0.054 |
| Multidrug and toxin extrusion protein 1 | SLC47A1 | Electrochemical transporter | 0.054 |
| Serotonin 1f (5-HT1f) receptor | HTR1F | Family A G protein-coupled receptor | 0.054 |
| Serotonin 3a (5-HT3a) receptor | HTR3A | Ligand-gated ion channel | 0.054 |
| Cathepsin L | CTSL | Protease | 0.054 |
| Alpha-1 6-mannosyl-glycoprotein 2-beta-N-acetylglucosaminyltransferase | MGAT2 | Enzyme | 0.054 |
| Serotonin 6 (5-HT6) receptor | HTR6 | Family A G protein-coupled receptor | 0.054 |
| Serotonin 5a (5-HT5a) receptor | HTR5A | Family A G protein-coupled receptor | 0.054 |
| Histamine H4 receptor | HRH4 | Family A G protein-coupled receptor | 0.054 |
| Dopamine D1 receptor | DRD1 | Family A G protein-coupled receptor | 0.054 |
| Thrombin and coagulation factor X | F10 | Protease | 0.054 |
| Metabotropic glutamate receptor 2 (by homology) | GRM2 | Family C G protein-coupled receptor | 0.054 |
| Butyrylcholinesterase | BCHE | Hydrolase | 0.054 |
| Delta opioid receptor | OPRD1 | Family A G protein-coupled receptor | 0.054 |
| Nociceptin receptor | OPRL1 | Family A G protein-coupled receptor | 0.054 |
| Serine/threonine-protein kinase PIM1 | PIM1 | Kinase | 0.054 |
| Serotonin 2b (5-HT2b) receptor | HTR2B | Family A G protein-coupled receptor | 0.054 |
| Adenosine A3 receptor | ADORA3 | Family A G protein-coupled receptor | 0.054 |
| Mu opioid receptor | OPRM1 | Family A G protein-coupled receptor | 0.054 |
| Poly [ADP-ribose] polymerase-1 | PARP1 | Enzyme | 0.054 |

| | | | |
|---|---|---|---|
| Neuronal acetylcholine receptor protein alpha-4 subunit | CHRNA4 | Ligand-gated ion channel | 0.054 |
| Neuronal acetylcholine receptor subunit alpha-3 | CHRNA3 | Ligand-gated ion channel | 0.054 |
| Dipeptidyl peptidase I | CTSC | Protease | 0.054 |
| Matrix metalloproteinase 3 | MMP3 | Protease | 0.054 |
| Cathepsin K | CTSK | Protease | 0.054 |
| Neuronal acetylcholine receptor; alpha4/beta4 | CHRNB4 CHRNA4 | Ligand-gated ion channel | 0.054 |
| Neuronal acetylcholine receptor; alpha3/beta2 | CHRNA3 CHRNB2 | Ligand-gated ion channel | 0.054 |
| Nicotinic acetylcholine receptor alpha2/beta4 | CHRNB4 CHRNA1 | Ligand-gated ion channel | 0.054 |
| Muscarinic acetylcholine receptor M4 | CHRM4 | Family A G protein-coupled receptor | 0.054 |
| Adrenergic receptor alpha-2 | ADRA2C | Family A G protein-coupled receptor | 0.054 |
| Alpha-2b adrenergic receptor | ADRA2B | Family A G protein-coupled receptor | 0.054 |
| Muscarinic acetylcholine receptor M5 | CHRM5 | Family A G protein-coupled receptor | 0.054 |
| Epidermal growth factor receptor erbB1 | EGFR | Kinase | 0.054 |
| Muscarinic acetylcholine receptor M2 | CHRM2 | Family A G protein-coupled receptor | 0.054 |
| Muscarinic acetylcholine receptor M3 | CHRM3 | Family A G protein-coupled receptor | 0.054 |
| ***Compound 10*** | | | |
| Acetylcholinesterase | ACHE | Hydrolase | 0.120 |
| Serotonin 6 (5-HT6) receptor | HTR6 | Family A G protein-coupled receptor | 0.112 |
| Muscarinic acetylcholine receptor M1 (by homology) | CHRM1 | Family A G protein-coupled receptor | 0.112 |
| Dopamine D4 receptor | DRD4 | Family A G protein-coupled receptor | 0.112 |
| Norepinephrine transporter | SLC6A2 | Electrochemical transporter | 0.112 |
| Serotonin transporter | SLC6A4 | Electrochemical transporter | 0.112 |
| 11-beta-hydroxysteroid dehydrogenase 1 | HSD11B1 | Enzyme | 0.112 |
| Serotonin 1a (5-HT1a) receptor | HTR1A | Family A G protein-coupled receptor | 0.112 |
| Multidrug and toxin extrusion protein 1 | SLC47A1 | Electrochemical transporter | 0.112 |
| Serotonin 1f (5-HT1f) receptor | HTR1F | Family A G protein-coupled receptor | 0.112 |
| Serotonin 1b (5-HT1b) receptor | HTR1B | Family A G protein-coupled receptor | 0.112 |
| Serotonin 3a (5-HT3a) receptor | HTR3A | Ligand-gated ion channel | 0.112 |
| Serotonin 1d (5-HT1d) receptor | HTR1D | Family A G protein-coupled receptor | 0.112 |
| Serotonin 1e (5-HT1e) receptor | HTR1E | Family A G protein-coupled receptor | 0.112 |
| Serotonin 2c (5-HT2c) receptor | HTR2C | Family A G protein-coupled receptor | 0.112 |
| Serotonin 5a (5-HT5a) receptor | HTR5A | Family A G protein-coupled receptor | 0.112 |
| Lethal(3)malignant brain tumor-like protein 3 | L3MBTL3 | Reader | 0.112 |
| Histamine H1 receptor | HRH1 | Family A G protein-coupled receptor | 0.112 |
| Glutamate [NMDA] receptor subunit epsilon 2 | GRIN2B | Ligand-gated ion channel | 0.112 |
| Dopamine D1 receptor | DRD1 | Family A G protein-coupled receptor | 0.112 |
| Dopamine transporter | SLC6A3 | Electrochemical transporter | 0.112 |
| Transient receptor potential cation channel subfamily V member 4 | TRPV4 | Voltage-gated ion channel | 0.112 |
| C-C chemokine receptor type 4 | CCR4 | Family A G protein-coupled receptor | 0.112 |
| Nociceptin receptor | OPRL1 | Family A G protein-coupled receptor | 0.112 |

| | | | |
|---|---|---|---|
| Neuronal acetylcholine receptor; alpha4/beta2 | CHRNA4 CHRNB2 | Ligand-gated ion channel | 0.112 |
| Metabotropic glutamate receptor 2 | GRM2 | Family C G protein-coupled receptor | 0.112 |
| Histamine H4 receptor | HRH4 | Family A G protein-coupled receptor | 0.112 |
| Rho-associated protein kinase | ROCK2 ROCK1 | Kinase | 0.112 |
| Somatostatin receptor 3 | SSTR3 | Family A G protein-coupled receptor | 0.112 |
| Neuronal acetylcholine receptor; alpha4/beta4 | CHRNB4 CHRNA4 | Ligand-gated ion channel | 0.112 |
| Neuronal acetylcholine receptor; alpha3/beta4 | CHRNA3 CHRNB4 | Ligand-gated ion channel | 0.112 |
| Neuronal acetylcholine receptor; alpha3/beta2 | CHRNA3 CHRNB2 | Ligand-gated ion channel | 0.112 |
| Nicotinic acetylcholine receptor alpha2/beta4 | CHRNB4 CHRNA1 | Ligand-gated ion channel | 0.112 |
| Progesterone receptor | PGR | Nuclear receptor | 0.112 |
| Phosphodiesterase 2A | PDE2A | Phosphodiesterase | 0.112 |
| Phosphodiesterase 10A (by homology) | PDE10A | Phosphodiesterase | 0.112 |
| Serine/threonine-protein kinase PIM1 | PIM1 | Kinase | 0.112 |
| Serine/threonine-protein kinase PIM2 | PIM2 | Kinase | 0.112 |
| Leukotriene A4 hydrolase | LTA4H | Protease | 0.112 |
| Monoamine oxidase A | MAOA | Oxidoreductase | 0.112 |
| Monoamine oxidase B | MAOB | Oxidoreductase | 0.112 |
| Lysine-specific histone demethylase 1 | KDM1A | Eraser | 0.112 |
| Synaptic vesicular amine transporter (by homology) | SLC18A2 | Electrochemical transporter | 0.112 |
| Calcium sensing receptor | CASR | Family C G protein-coupled receptor | 0.112 |
| Alpha-2a adrenergic receptor | ADRA2A | Family A G protein-coupled receptor | 0.112 |
| Serotonin 2b (5-HT2b) receptor | HTR2B | Family A G protein-coupled receptor | 0.112 |
| Alpha-16-mannosyl-glycoprotein 2-beta-N-acetylglucosaminyltransferase | MGAT2 | Enzyme | 0.112 |
| Telomerase reverse transcriptase | TERT | Enzyme | 0.112 |
| Dopamine D5 receptor | DRD5 | Family A G protein-coupled receptor | 0.112 |
| Muscarinic acetylcholine receptor M3 | CHRM3 | Family A G protein-coupled receptor | 0.112 |
| Translocator protein (by homology) | TSPO | Membrane receptor | 0.112 |
| Glutamate NMDA receptor; GRIN1/GRIN2B | GRIN1 GRIN2B | Ligand-gated ion channel | 0.112 |
| Cathepsin S | CTSS | Protease | 0.112 |
| Rho-associated protein kinase 2 | ROCK2 | Kinase | 0.112 |
| ***Compound 13*** | | | |
| Serotonin 7 (5-HT7) receptor | HTR7 | Family A G protein-coupled receptor | 0.535 |
| Serotonin 2a (5-HT2a) receptor | HTR2A | Family A G protein-coupled receptor | 0.525 |
| Serotonin 2c (5-HT2c) receptor | HTR2C | Family A G protein-coupled receptor | 0.525 |
| Serotonin 1b (5-HT1b) receptor | HTR1B | Family A G protein-coupled receptor | 0.444 |
| Serotonin 1d (5-HT1d) receptor | HTR1D | Family A G protein-coupled receptor | 0.444 |
| Dopamine D2 receptor | DRD2 | Family A G protein-coupled receptor | 0.444 |
| Dopamine D3 receptor | DRD3 | Family A G protein-coupled receptor | 0.444 |
| Serotonin 1a (5-HT1a) receptor | HTR1A | Family A G protein-coupled receptor | 0.426 |
| Serotonin 2b (5-HT2b) receptor | HTR2B | Family A G protein-coupled receptor | 0.280 |
| Serotonin transporter | SLC6A4 | Electrochemical transporter | 0.217 |
| Alpha-1b adrenergic receptor | ADRA1B | Family A G protein-coupled receptor | 0.181 |

| | | | |
|---|---|---|---|
| Serotonin 6 (5-HT6) receptor | HTR6 | Family A G protein-coupled receptor | 0.181 |
| Serotonin 5a (5-HT5a) receptor | HTR5A | Family A G protein-coupled receptor | 0.181 |
| HERG | KCNH2 | Voltage-gated ion channel | 0.153 |
| Serotonin 1e (5-HT1e) receptor | HTR1E | Family A G protein-coupled receptor | 0.090 |
| Lethal(3)malignant brain tumor-like protein 3 | L3MBTL3 | Reader | 0.090 |
| Acetylcholinesterase | ACHE | Hydrolase | 0.081 |
| Norepinephrine transporter | SLC6A2 | Electrochemical transporter | 0.063 |
| Serotonin 4 (5-HT4) receptor | HTR4 | Family A G protein-coupled receptor | 0.063 |
| Carbonic anhydrase II | CA2 | Lyase | 0.063 |
| Carbonic anhydrase XII | CA12 | Lyase | 0.063 |
| Carbonic anhydrase IX | CA9 | Lyase | 0.063 |
| Sigma opioid receptor | SIGMAR1 | Membrane receptor | 0.054 |
| Glycine transporter 1 | SLC6A9 | Electrochemical transporter | 0.054 |
| 11-beta-hydroxysteroid dehydrogenase 1 | HSD11B1 | Enzyme | 0.054 |
| Cathepsin K | CTSK | Protease | 0.054 |
| Cathepsin L | CTSL | Protease | 0.054 |
| Cathepsin (B and K) | CTSB | Protease | 0.054 |
| Serotonin 1f (5-HT1f) receptor | HTR1F | Family A G protein-coupled receptor | 0.054 |
| Multidrug and toxin extrusion protein 1 | SLC47A1 | Electrochemical transporter | 0.054 |
| Serotonin 3a (5-HT3a) receptor | HTR3A | Ligand-gated ion channel | 0.054 |
| Phosphodiesterase 2A | PDE2A | Phosphodiesterase | 0.054 |
| Phosphodiesterase 10A (by homology) | PDE10A | Phosphodiesterase | 0.054 |
| Beta amyloid A4 protein | APP | Membrane receptor | 0.054 |
| Lanosterol synthase | LSS | Enzyme | 0.054 |
| Neuronal acetylcholine receptor; alpha4/beta4 | CHRNB4 CHRNA4 | Ligand-gated ion channel | 0.054 |
| Neuronal acetylcholine receptor; alpha3/beta4 | CHRNA3 CHRNB4 | Ligand-gated ion channel | 0.054 |
| Neuronal acetylcholine receptor; alpha3/beta2 | CHRNA3 CHRNB2 | Ligand-gated ion channel | 0.054 |
| Nicotinic acetylcholine receptor alpha2/beta4 | CHRNB4 CHRNA1 | Ligand-gated ion channel | 0.054 |
| Dopamine D1 receptor | DRD1 | Family A G protein-coupled receptor | 0.054 |
| Dipeptidyl peptidase IV | DPP4 | Protease | 0.054 |
| Gamma-secretase | PSEN2 PSENEN NCSTN APH1A PSEN1 APH1B | Protease | 0.054 |
| Muscarinic acetylcholine receptor M4 | CHRM4 | Family A G protein-coupled receptor | 0.054 |
| Butyrylcholinesterase | BCHE | Hydrolase | 0.054 |
| Alpha-2b adrenergic receptor | ADRA2B | Family A G protein-coupled receptor | 0.054 |
| Muscarinic acetylcholine receptor M5 | CHRM5 | Family A G protein-coupled receptor | 0.054 |
| Muscarinic acetylcholine receptor M3 | CHRM3 | Family A G protein-coupled receptor | 0.054 |
| Toll-like receptor (TLR7/TLR9) | TLR9 | Toll-like and Il-1 receptors | 0.054 |
| Monoamine oxidase B | MAOB | Oxidoreductase | 0.054 |
| Lysine-specific histone demethylase 1 | KDM1A | Eraser | 0.054 |
| Glucose-6-phosphate 1-dehydrogenase | G6PD | Enzyme | 0.054 |
| Cytochrome P450 19A1 | CYP19A1 | Cytochrome P450 | 0.054 |
| Histamine H3 receptor | HRH3 | Family A G protein-coupled receptor | 0.054 |

| | | | |
|---|---|---|---|
| Neuronal acetylcholine receptor protein alpha-4 subunit | CHRNA4 | Ligand-gated ion channel | 0.054 |
| Neuronal acetylcholine receptor subunit alpha-3 | CHRNA3 | Ligand-gated ion channel | 0.054 |
| Neuronal acetylcholine receptor protein alpha-7 subunit | CHRNA7 | Ligand-gated ion channel | 0.054 |
| Beta-1 adrenergic receptor | ADRB1 | Family A G protein-coupled receptor | 0.054 |
| Monoamine oxidase A | MAOA | Oxidoreductase | 0.054 |
| Histamine H4 receptor | HRH4 | Family A G protein-coupled receptor | 0.054 |
| Lethal(3)malignant brain tumor-like protein 1 | L3MBTL1 | Reader | 0.054 |
| MBT domain-containing protein 1 | MBTD1 | Reader | 0.054 |
| Voltage-gated N-type calcium channel alpha-1B subunit | CACNA1B | Voltage-gated ion channel | 0.054 |
| Nociceptin receptor | OPRL1 | Family A G protein-coupled receptor | 0.054 |
| MAP kinase p38 alpha | MAPK14 | Kinase | 0.054 |
| Dopamine D5 receptor | DRD5 | Family A G protein-coupled receptor | 0.054 |
| Neuropeptide Y receptor type 5 | NPY5R | Family A G protein-coupled receptor | 0.054 |
| Myeloperoxidase | MPO | Enzyme | 0.054 |
| Nitric oxide synthase inducible | NOS2 | Enzyme | 0.054 |
| Thromboxane A2 receptor | TBXA2R | Family A G protein-coupled receptor | 0.054 |
| ***Compound 14*** | | | |
| Serotonin 2a (5-HT2a) receptor | HTR2A | Family A G protein-coupled receptor | 0.308 |
| Serotonin 2c (5-HT2c) receptor | HTR2C | Family A G protein-coupled receptor | 0.308 |
| Serotonin 7 (5-HT7) receptor | HTR7 | Family A G protein-coupled receptor | 0.230 |
| Serotonin 1b (5-HT1b) receptor | HTR1B | Family A G protein-coupled receptor | 0.214 |
| Serotonin 1d (5-HT1d) receptor | HTR1D | Family A G protein-coupled receptor | 0.214 |
| Serotonin 1a (5-HT1a) receptor | HTR1A | Family A G protein-coupled receptor | 0.214 |
| Dopamine D2 receptor | DRD2 | Family A G protein-coupled receptor | 0.214 |
| Dopamine D3 receptor | DRD3 | Family A G protein-coupled receptor | 0.214 |
| Serotonin 2b (5-HT2b) receptor | HTR2B | Family A G protein-coupled receptor | 0.159 |
| Carbonic anhydrase II | CA2 | Lyase | 0.143 |
| Carbonic anhydrase XII | CA12 | Lyase | 0.143 |
| Carbonic anhydrase IX | CA9 | Lyase | 0.143 |
| Serotonin transporter | SLC6A4 | Electrochemical transporter | 0.143 |
| Alpha-1b adrenergic receptor | ADRA1B | Family A G protein-coupled receptor | 0.143 |
| HERG | KCNH2 | Voltage-gated ion channel | 0.136 |
| Acetylcholinesterase | ACHE | Hydrolase | 0.136 |
| Serotonin 6 (5-HT6) receptor | HTR6 | Family A G protein-coupled receptor | 0.136 |
| Serotonin 5a (5-HT5a) receptor | HTR5A | Family A G protein-coupled receptor | 0.128 |
| Lethal(3)malignant brain tumor-like protein 3 | L3MBTL3 | Reader | 0.128 |
| 11-beta-hydroxysteroid dehydrogenase 1 | HSD11B1 | Enzyme | 0.120 |
| Serotonin 1e (5-HT1e) receptor | HTR1E | Family A G protein-coupled receptor | 0.112 |
| Norepinephrine transporter | SLC6A2 | Electrochemical transporter | 0.112 |
| Serotonin 4 (5-HT4) receptor | HTR4 | Family A G protein-coupled receptor | 0.112 |
| Sigma opioid receptor | SIGMAR1 | Membrane receptor | 0.112 |
| Voltage-gated N-type calcium channel alpha-1B subunit | CACNA1B | Voltage-gated ion channel | 0.112 |

| | | | |
|---|---|---|---|
| Phosphodiesterase 2A | PDE2A | Phosphodiesterase | 0.112 |
| Phosphodiesterase 10A (by homology) | PDE10A | Phosphodiesterase | 0.112 |
| Histamine H3 receptor | HRH3 | Family A G protein-coupled receptor | 0.112 |
| Histamine H4 receptor | HRH4 | Family A G protein-coupled receptor | 0.112 |
| Cathepsin K | CTSK | Protease | 0.112 |
| Epoxide hydratase | EPHX2 | Protease | 0.112 |
| Serotonin 1f (5-HT1f) receptor | HTR1F | Family A G protein-coupled receptor | 0.112 |
| Lanosterol synthase | LSS | Enzyme | 0.112 |
| Glycine transporter 1 | SLC6A9 | Electrochemical transporter | 0.112 |
| Neuropeptide Y receptor type 5 | NPY5R | Family A G protein-coupled receptor | 0.112 |
| Dopamine D1 receptor | DRD1 | Family A G protein-coupled receptor | 0.112 |
| Nociceptin receptor | OPRL1 | Family A G protein-coupled receptor | 0.112 |
| Beta-1 adrenergic receptor | ADRB1 | Family A G protein-coupled receptor | 0.112 |
| Multidrug and toxin extrusion protein 1 | SLC47A1 | Electrochemical transporter | 0.112 |
| Serotonin 3a (5-HT3a) receptor | HTR3A | Ligand-gated ion channel | 0.112 |
| Glutamate [NMDA] receptor subunit epsilon 2 | GRIN2B | Ligand-gated ion channel | 0.112 |
| Neuropeptide Y receptor type 1 | NPY1R | Family A G protein-coupled receptor | 0.112 |
| Cytochrome P450 19A1 | CYP19A1 | Cytochrome P450 | 0.112 |
| Muscarinic acetylcholine receptor M4 | CHRM4 | Family A G protein-coupled receptor | 0.112 |
| Butyrylcholinesterase | BCHE | Hydrolase | 0.112 |
| Alpha-2b adrenergic receptor | ADRA2B | Family A G protein-coupled receptor | 0.112 |
| Muscarinic acetylcholine receptor M5 | CHRM5 | Family A G protein-coupled receptor | 0.112 |
| Neuronal acetylcholine receptor; alpha4/beta4 | CHRNB4 CHRNA4 | Ligand-gated ion channel | 0.112 |
| Neuronal acetylcholine receptor; alpha3/beta4 | CHRNA3 CHRNB4 | Ligand-gated ion channel | 0.112 |
| Neuronal acetylcholine receptor; alpha3/beta2 | CHRNA3 CHRNB2 | Ligand-gated ion channel | 0.112 |
| Nicotinic acetylcholine receptor alpha2/beta4 | CHRNB4 CHRNA1 | Ligand-gated ion channel | 0.112 |
| Protein-glutamine gamma-glutamyltransferase | TGM2 | Enzyme | 0.112 |
| Neuronal acetylcholine receptor protein alpha-7 subunit | CHRNA7 | Ligand-gated ion channel | 0.112 |
| Somatostatin receptor 3 | SSTR3 | Family A G protein-coupled receptor | 0.112 |
| Acetylcholine receptor; alpha1/beta1/delta/gamma | CHRNB1 CHRNA1 CHRNG CHRND | Ligand-gated ion channel | 0.112 |
| Multidrug resistance-associated protein 1 | ABCC1 | Primary active transporter | 0.112 |
| P-glycoprotein 1 (by homology) | ABCB1 | Primary active transporter | 0.112 |
| Neuronal acetylcholine receptor subunit alpha-3 | CHRNA3 | Ligand-gated ion channel | 0.112 |
| Urotensin II receptor | UTS2R | Family A G protein-coupled receptor | 0.112 |
| Monoamine oxidase B | MAOB | Oxidoreductase | 0.112 |
| Lysine-specific histone demethylase 1 | KDM1A | Eraser | 0.112 |
| Cytochrome b-245 heavy chain | CYBB | Transmembrane 1-electron transfer carriers | 0.112 |
| Cathepsin L | CTSL | Protease | 0.112 |
| Cathepsin (B and K) | CTSB | Protease | 0.112 |

| | | | |
|---|---|---|---|
| Neuronal acetylcholine receptor protein alpha-4 subunit | CHRNA4 | Ligand-gated ion channel | 0.112 |
| Monoamine oxidase A | MAOA | Oxidoreductase | 0.112 |
| Serine/threonine-protein kinase PIM1 | PIM1 | Kinase | 0.112 |
| Myeloperoxidase | MPO | Enzyme | 0.112 |
| Serine/threonine-protein kinase PIM2 | PIM2 | Kinase | 0.112 |
| Protein farnesyltransferase | FNTA FNTB | Enzyme | 0.112 |
| Neuronal acetylcholine receptor; alpha3/alpha6/beta2/beta3 | CHRNB3 CHRNA6 CHRNB2 CHRNA3 | Ligand-gated ion channel | 0.112 |

**Figure S2**: $^{1}$H-NMR spectra (C*D*Cl$_3$) for *N-(2-(pyridin-2-yl)ethyl)benzenesulfonamide*, **1**

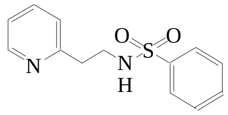


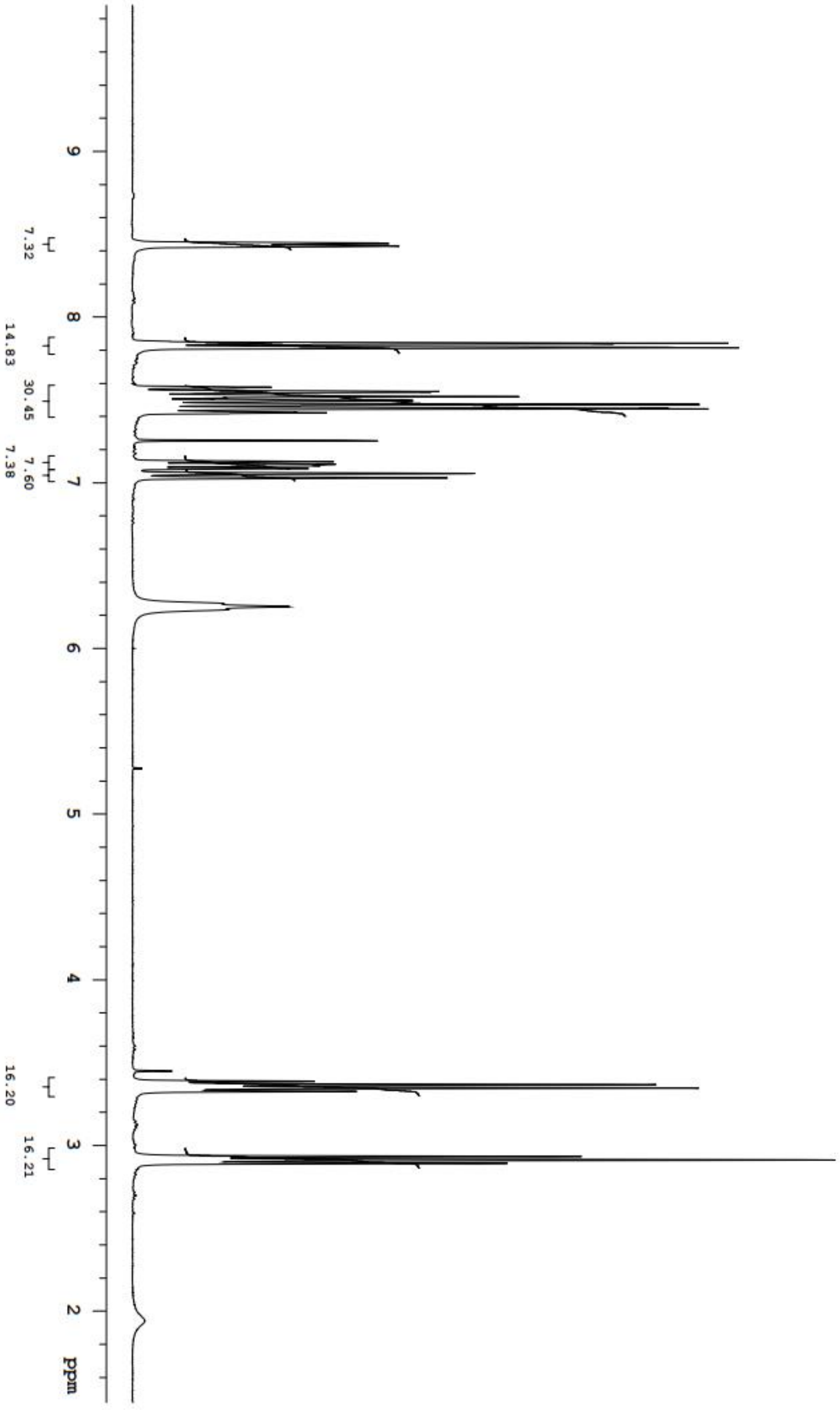

**Figure S3**: $^{13}$C-NMR spectra ($CDCl_3$) for *N-(2-(pyridin-2-yl)ethyl)benzenesulfonamide*, **1**

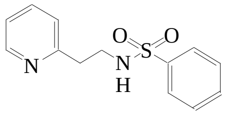


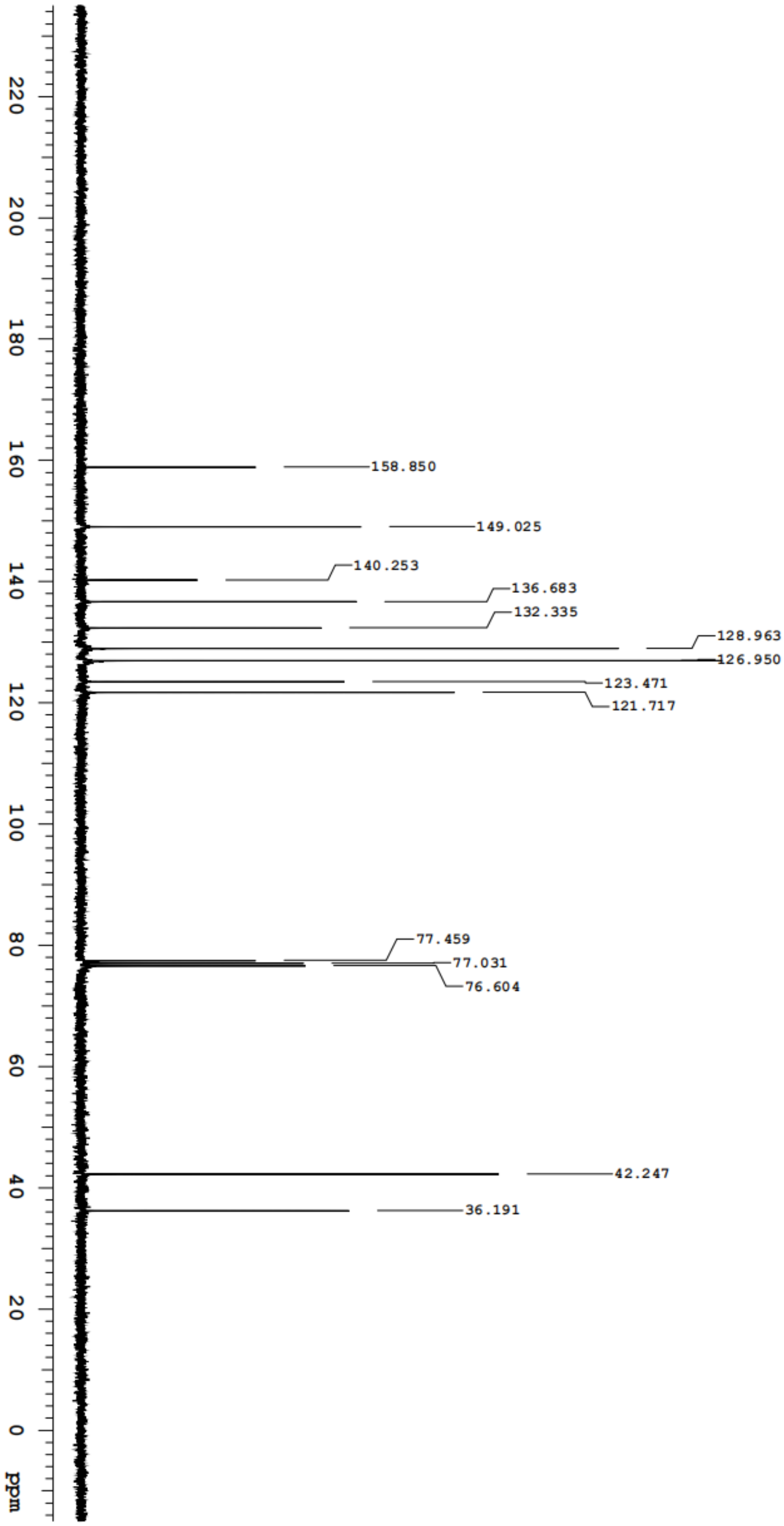

**Figure S4**: $^{1}$H-NMR spectra ($CD_3OD$) for *4-methyl-N-(2-(pyridin-2-yl)ethyl)benzenesulfonamide*, **2**

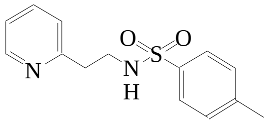


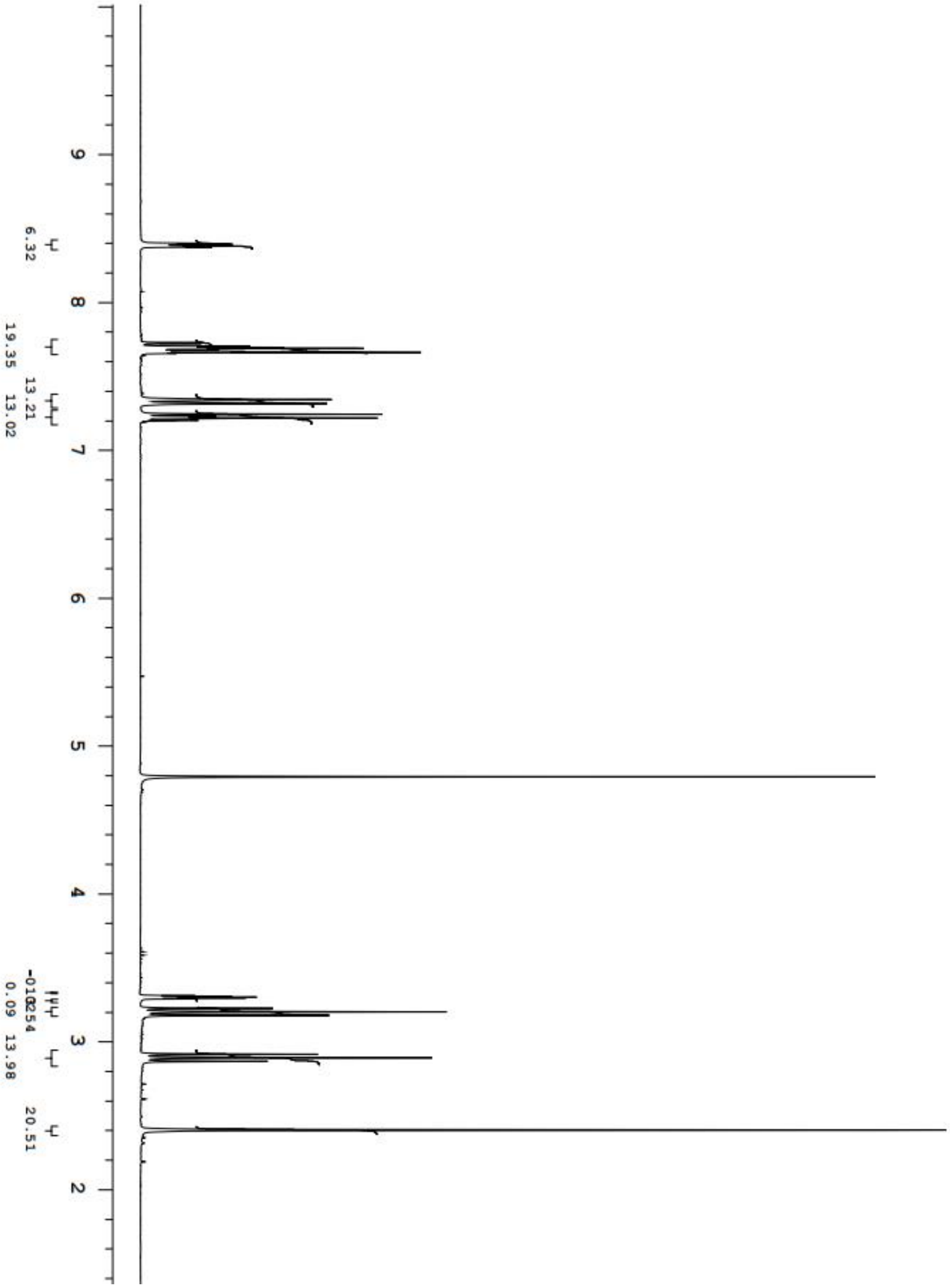

**Figure S5**: $^{13}$C-NMR spectra ($CD_3OD$) for *4-methyl-N-(2-(pyridin-2-yl)ethyl)benzenesulfonamide*, **2**

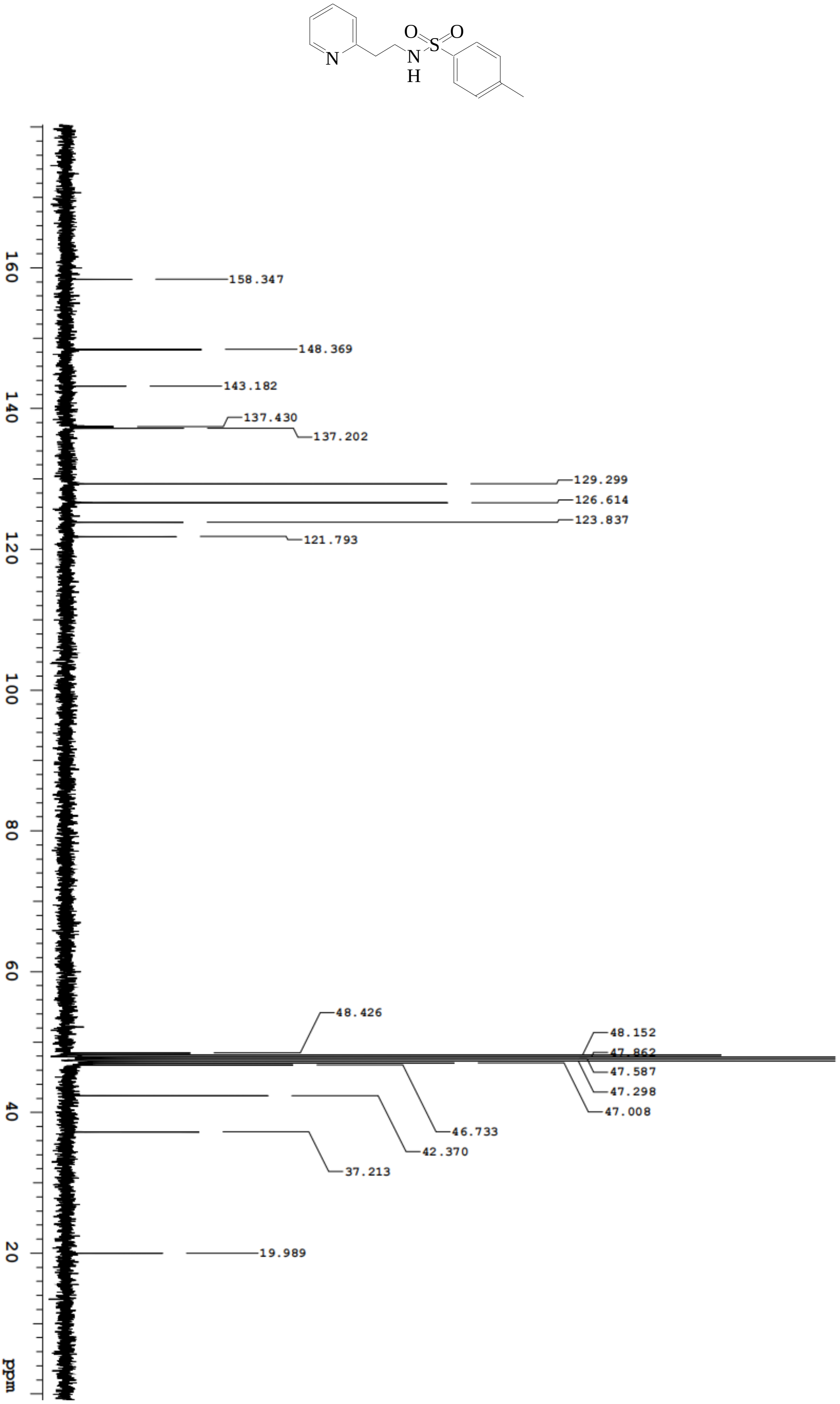

**Figure S6**: $^{1}$H-NMR spectra ($CDCl_3$) for *4-metyl-N-(pyridin-2-yl)benzenesulfonamide,* **3**

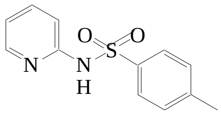


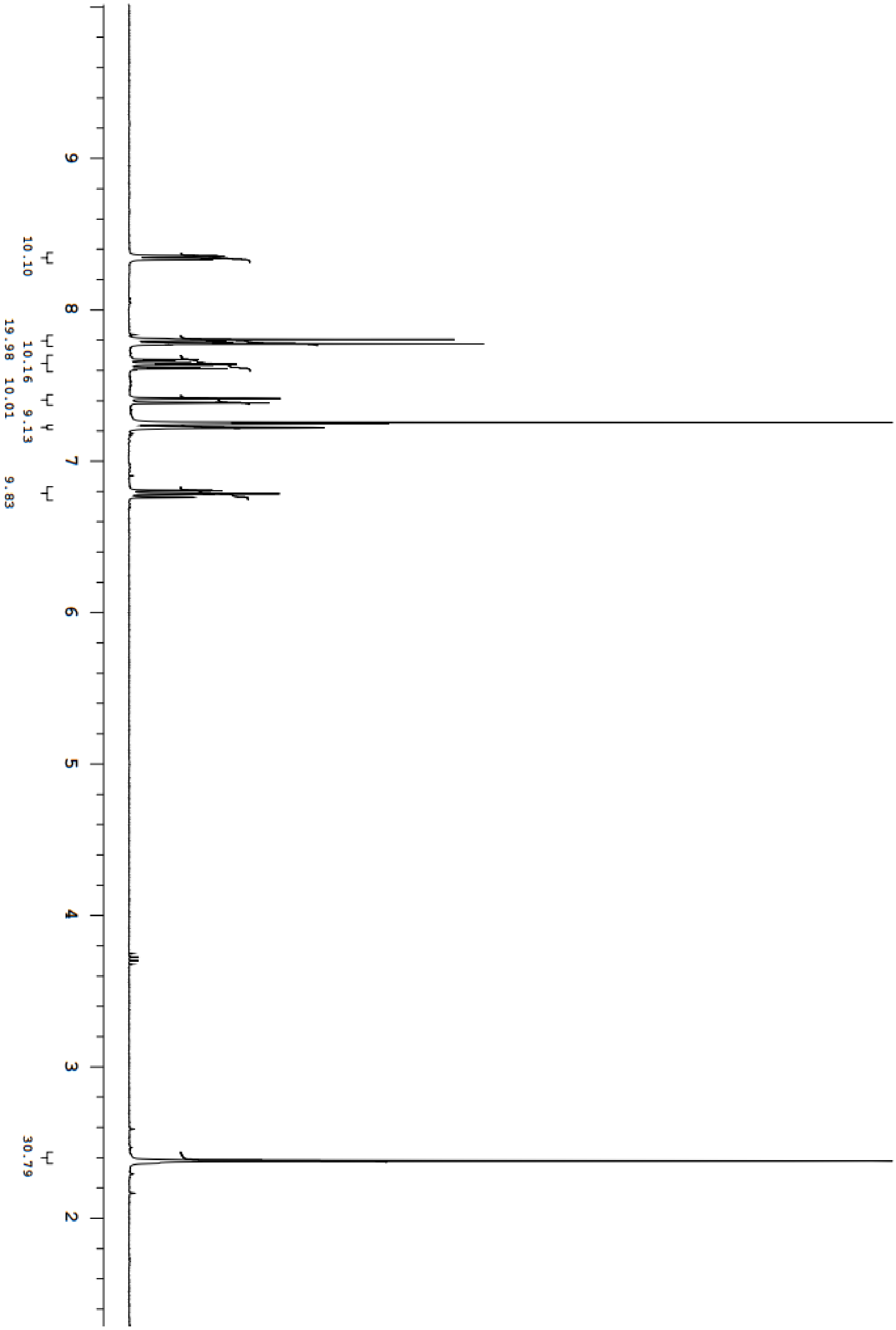

**Figure S7**: $^{13}$C-NMR spectra ($CDCl_3$) for *4-metyl-N-(pyridin-2-yl)benzenesulfonamide*, **3**

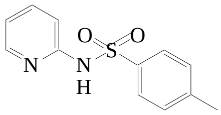


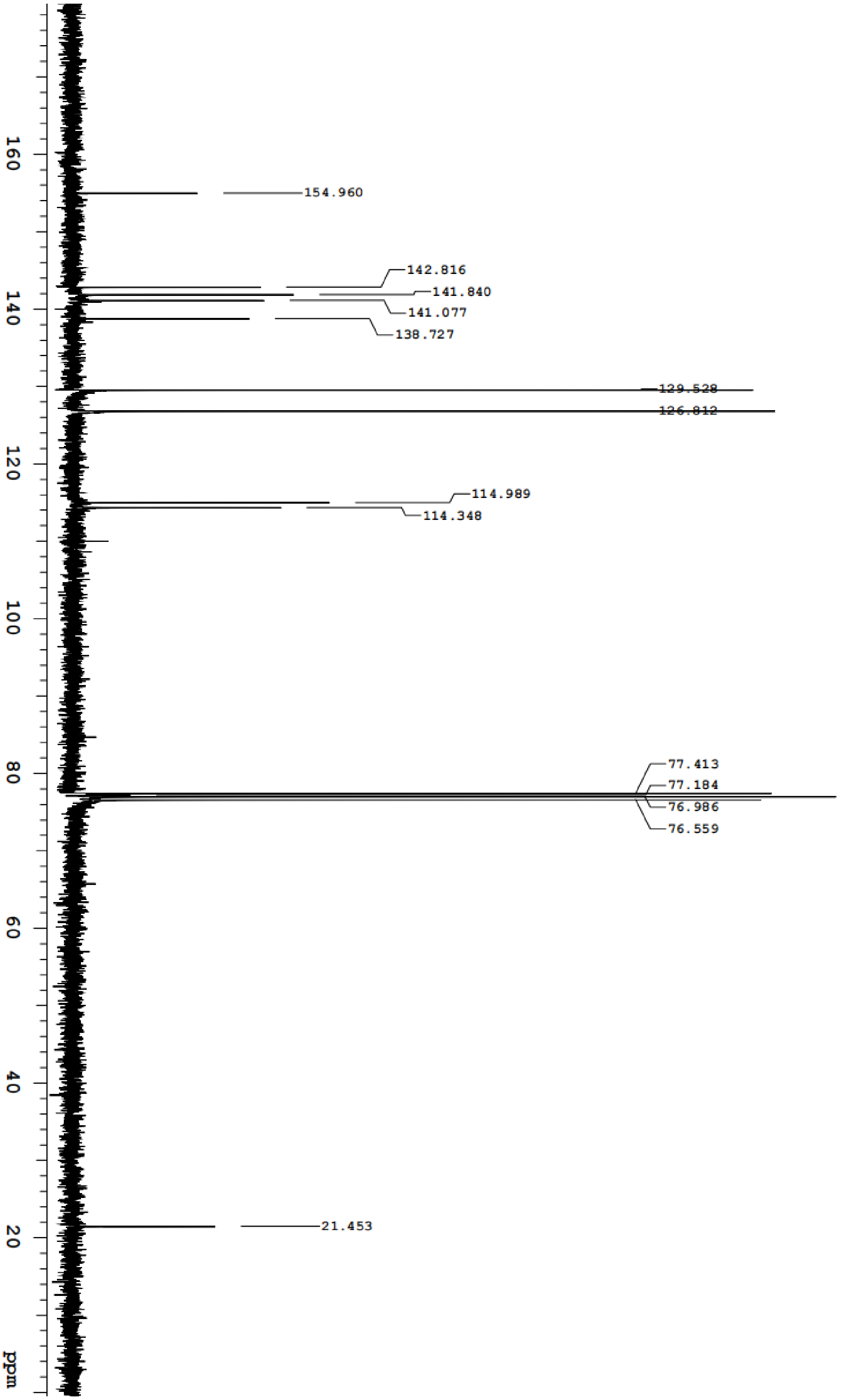

**Figure S8**: $^{1}$H-NMR spectra (C*D*Cl$_3$) for *N-(5-chloropyridin-2-yl)-4-methylbenzenesulfonamide*, **4**

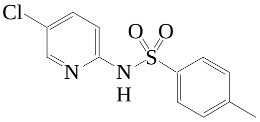


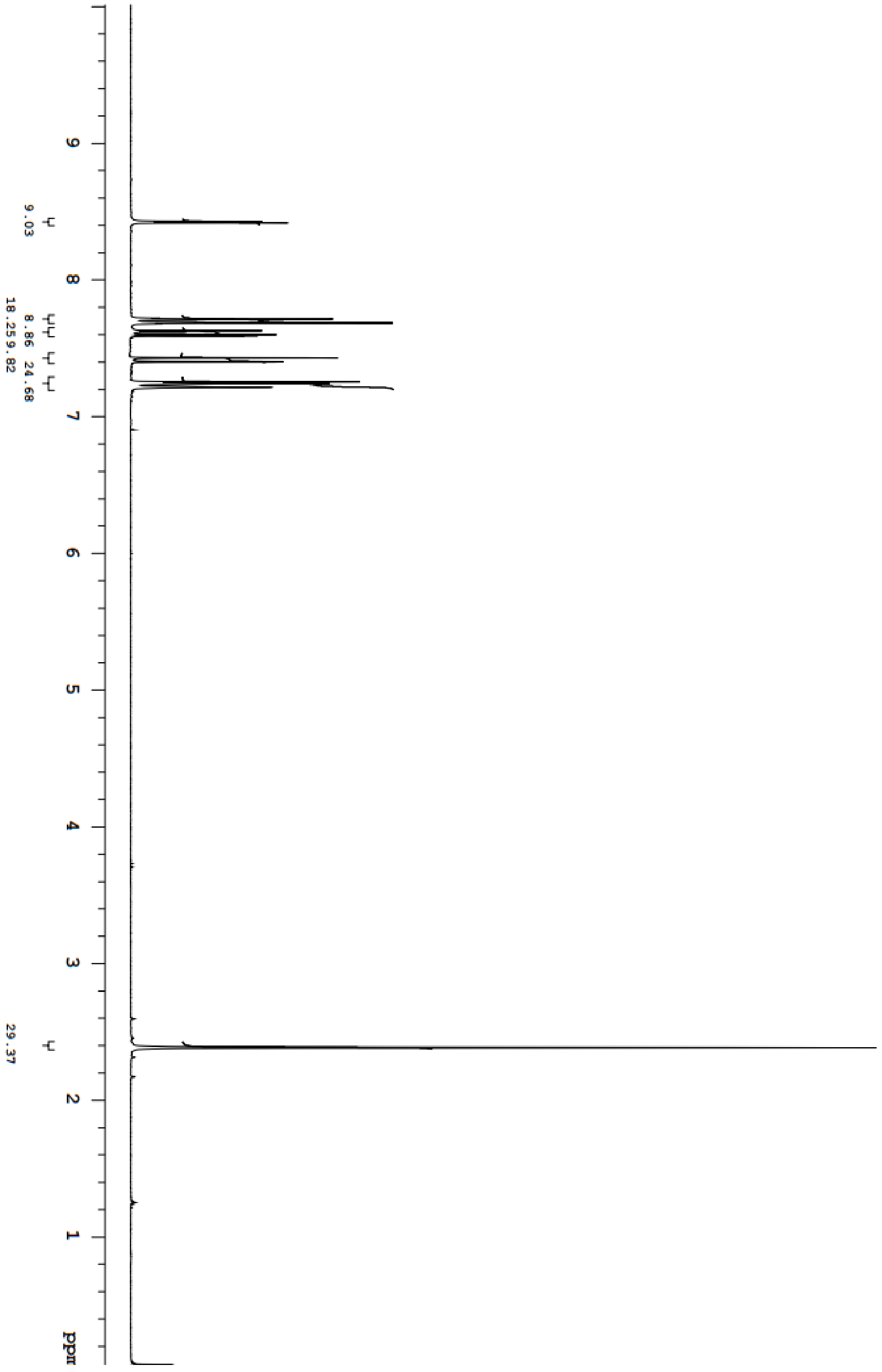

**Figure S9**: $^{13}$C-NMR spectra (C*D*Cl$_3$) for *N-(5-chloropyridin-2-yl)-4-methylbenzenesulfonamide*, **4**

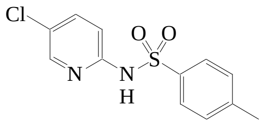


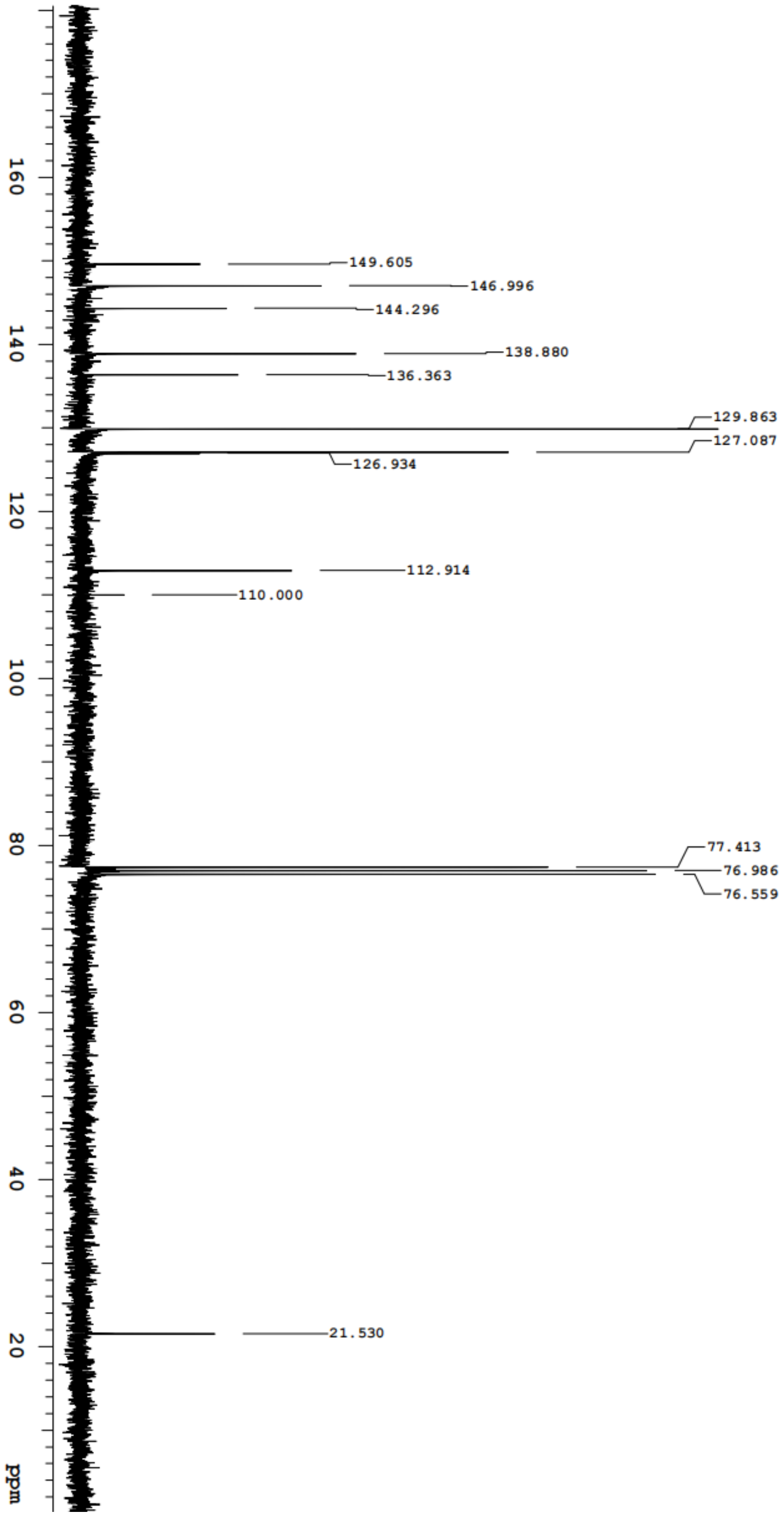

**Figure S10**: $^{1}$H-NMR spectra ($CD_3OD$) for *N-(3,5-dichloropyridin-2-yl)-4-methylbenzenesulfonamide*, **5**

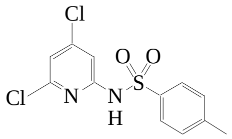


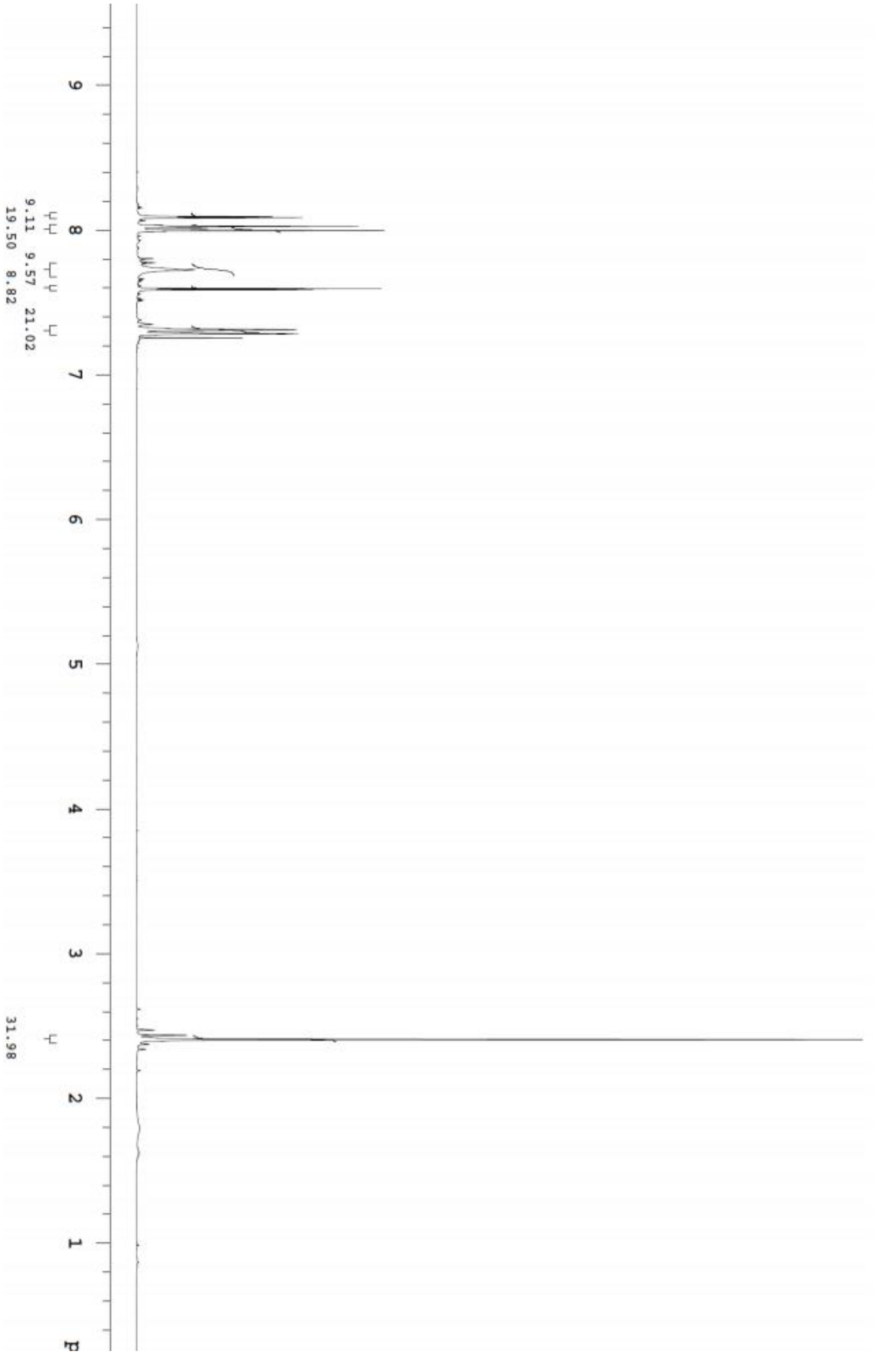

**Figure S11**: $^{13}$C-NMR spectra ($CDCl_3$) for *N-(3,5-dichloropyridin-2-yl)-4-methylbenzenesulfonamide*, **5**

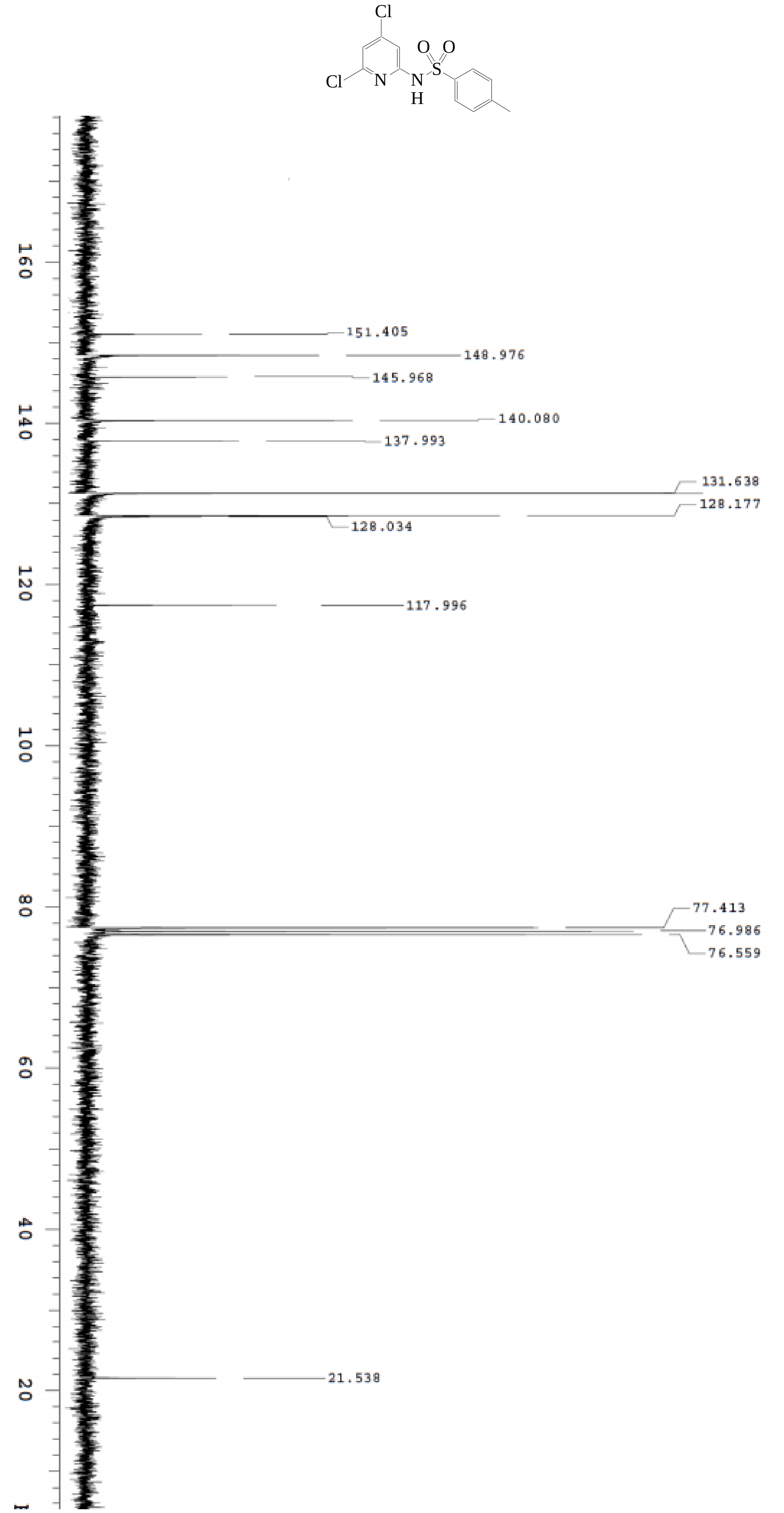

**Figure S12**: $^{1}$H-NMR spectra (C*D*Cl3) for *N-(3,5-dichloropyridin-2-yl)-1-phenylmethanesulfonamide*, **6**

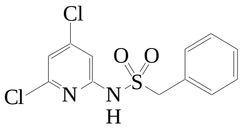


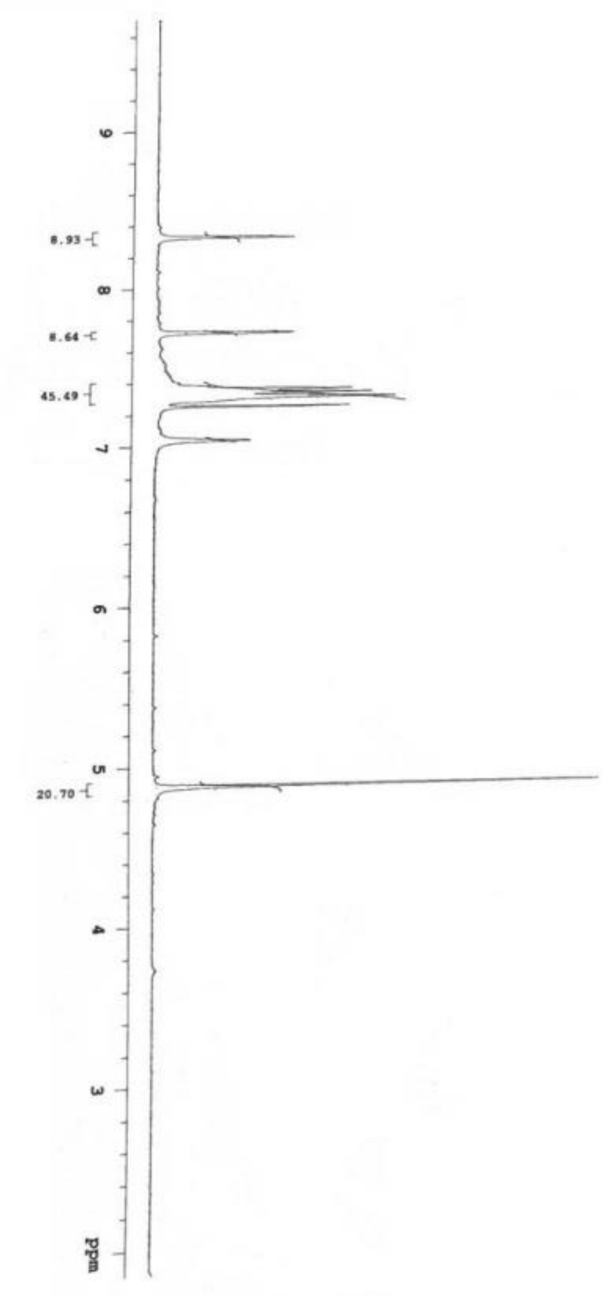

**Figure S13**: $^{13}$C-NMR spectra (C*D*Cl$_3$) for *N-(3,5-dichloropyridin-2-yl)-1-phenylmethanesulfonamide*, **6**

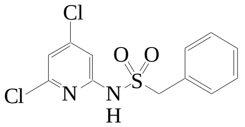


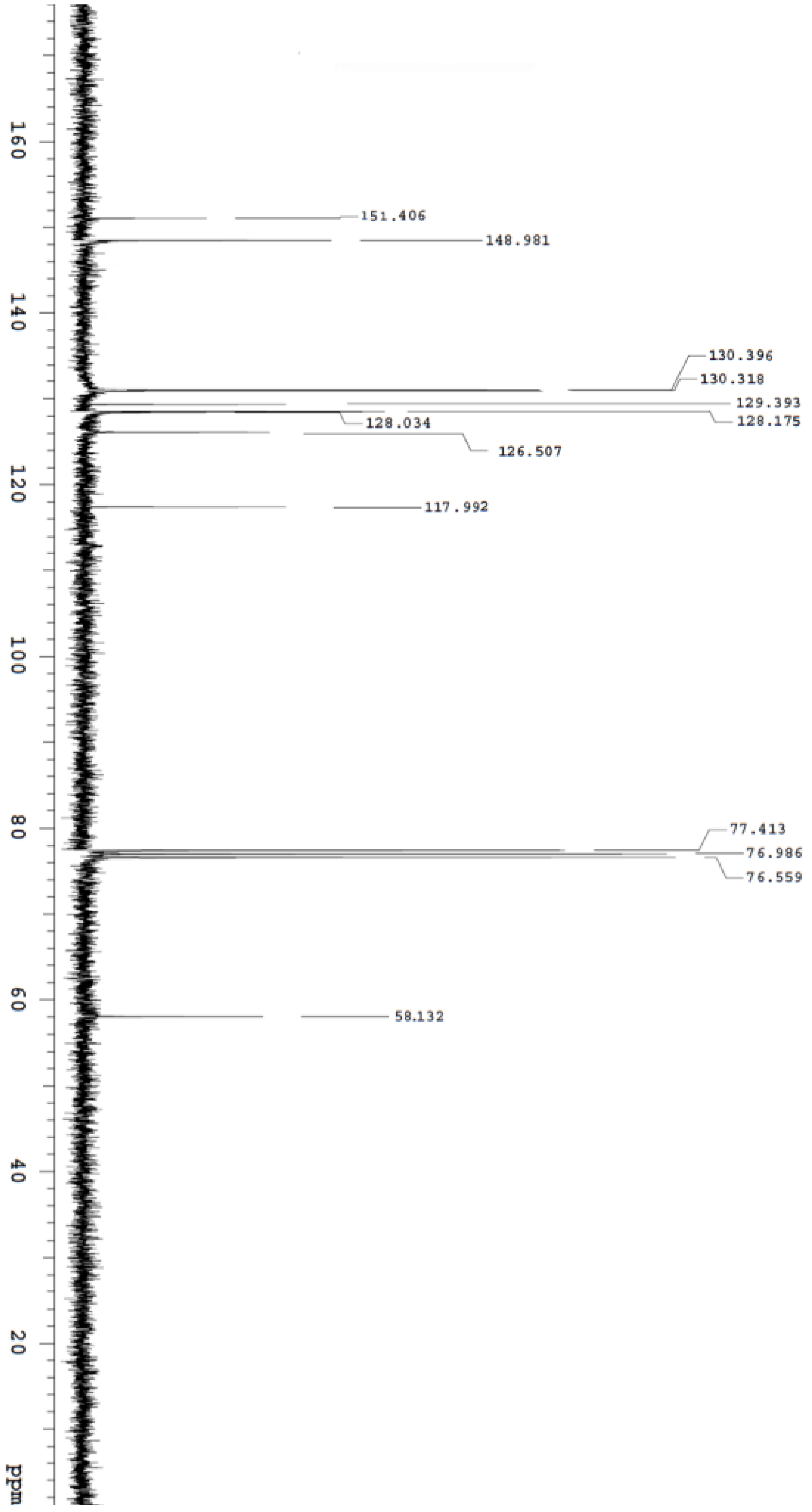

**Figure S14**: $^{1}$H-NMR spectra ($CD_3OD$) for *N-(1-benzylpiperidin-4-yl)benzenesulfonamide*, **7**

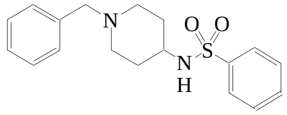


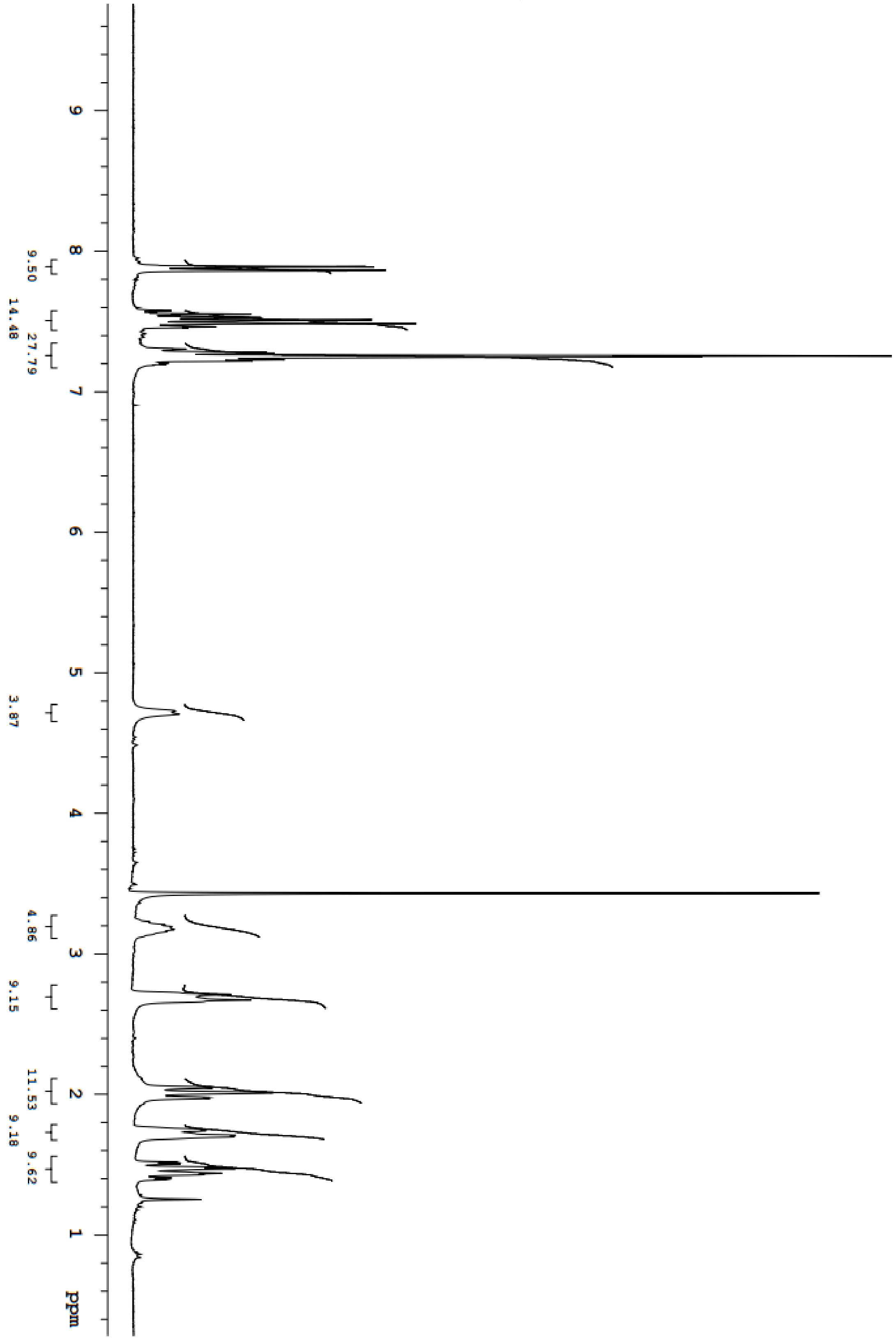

**Figure S15**: $^{13}$C-NMR spectra ($CD_3OD$) for *N-(1-benzylpiperidin-4-yl)benzenesulfonamide*, **7**

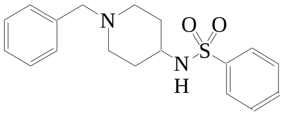


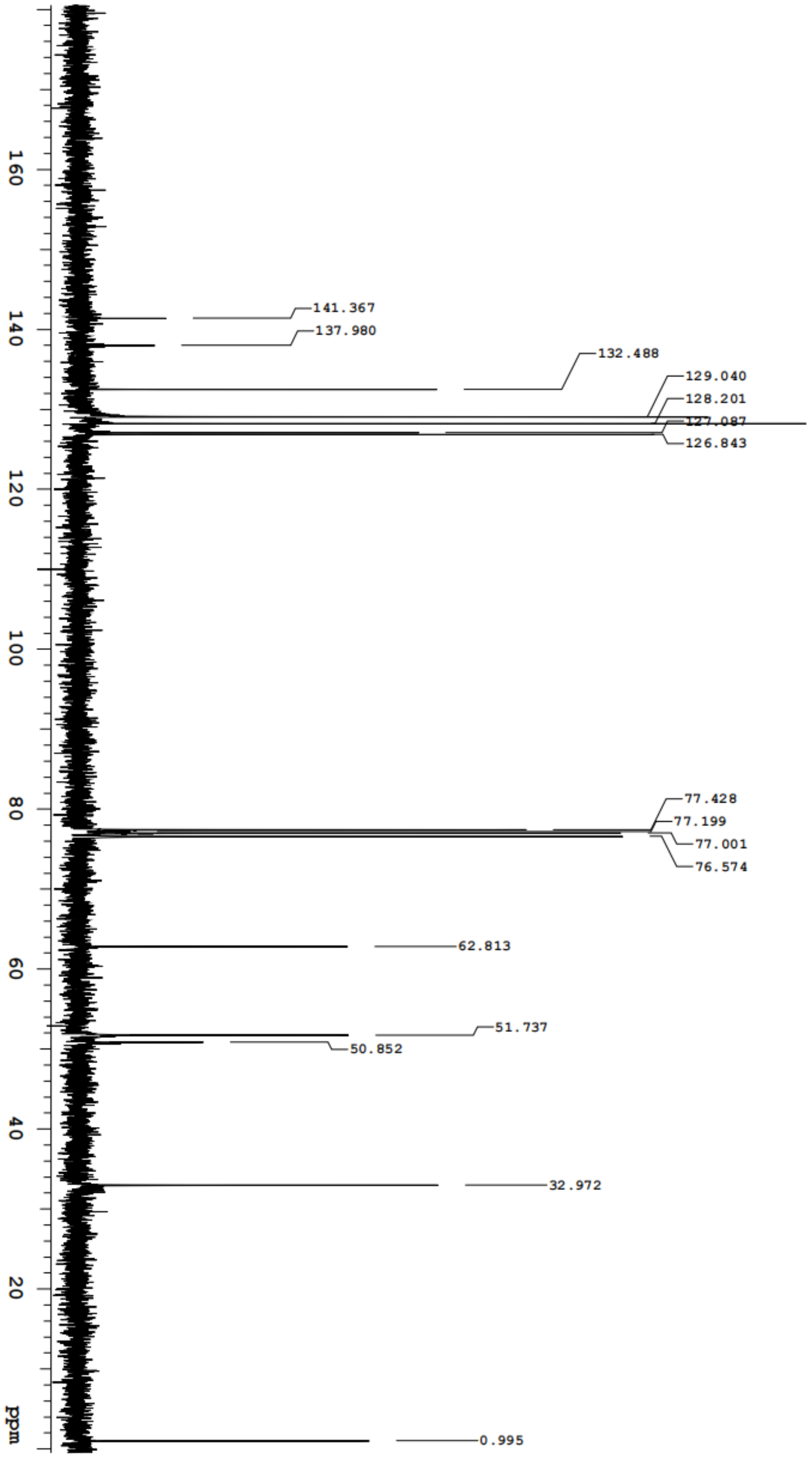

**Figure S16**: $^{1}$H-NMR spectra ($CD_3OD$) for *N-(1-benzylpiperidin-4-yl)-4-methylbenzenesulfonamide*, **8**

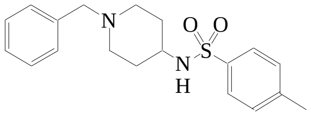

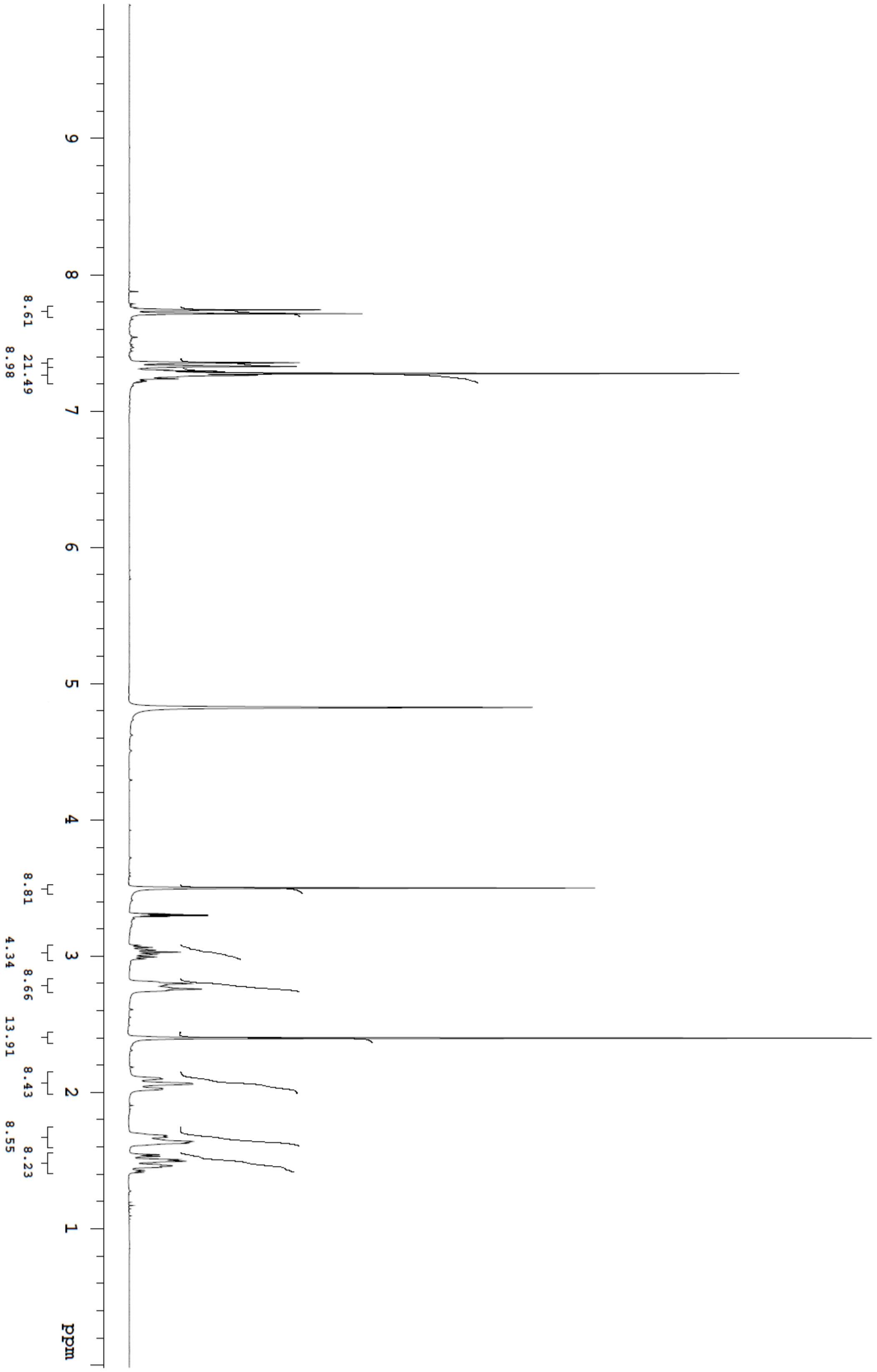

**Figure S17**: $^{13}$C-NMR spectra ($CD_3OD$) for *N-(1-benzylpiperidin-4-yl)-4-methylbenzenesulfonamide*, **8**

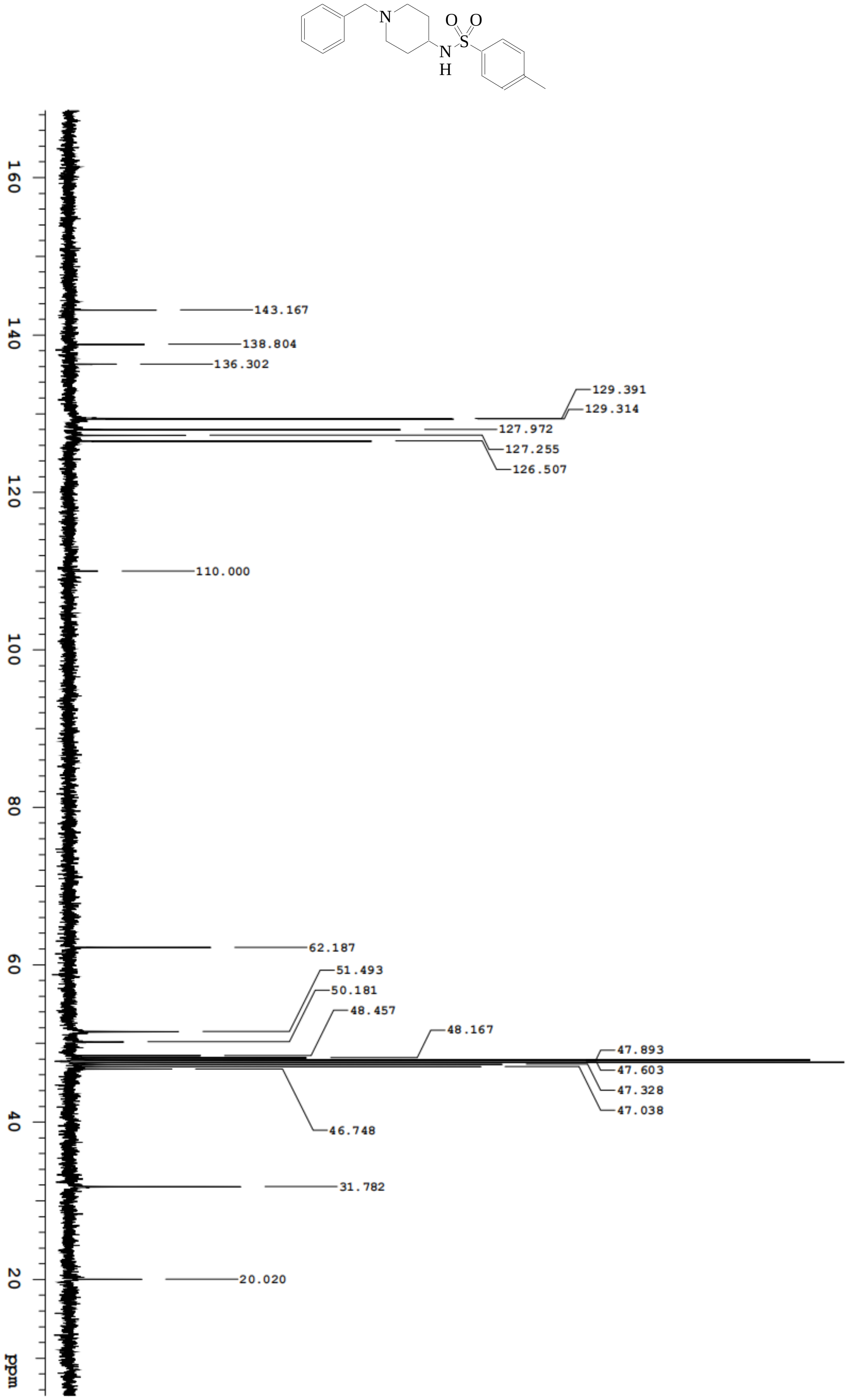

**Figure S18**: $^{1}$H-NMR spectra (C*D*Cl3) for *N-(2-(piperidin-1-yl)ethyl)benzenesulfonamide,* **9**

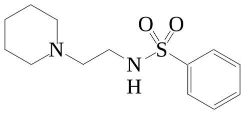

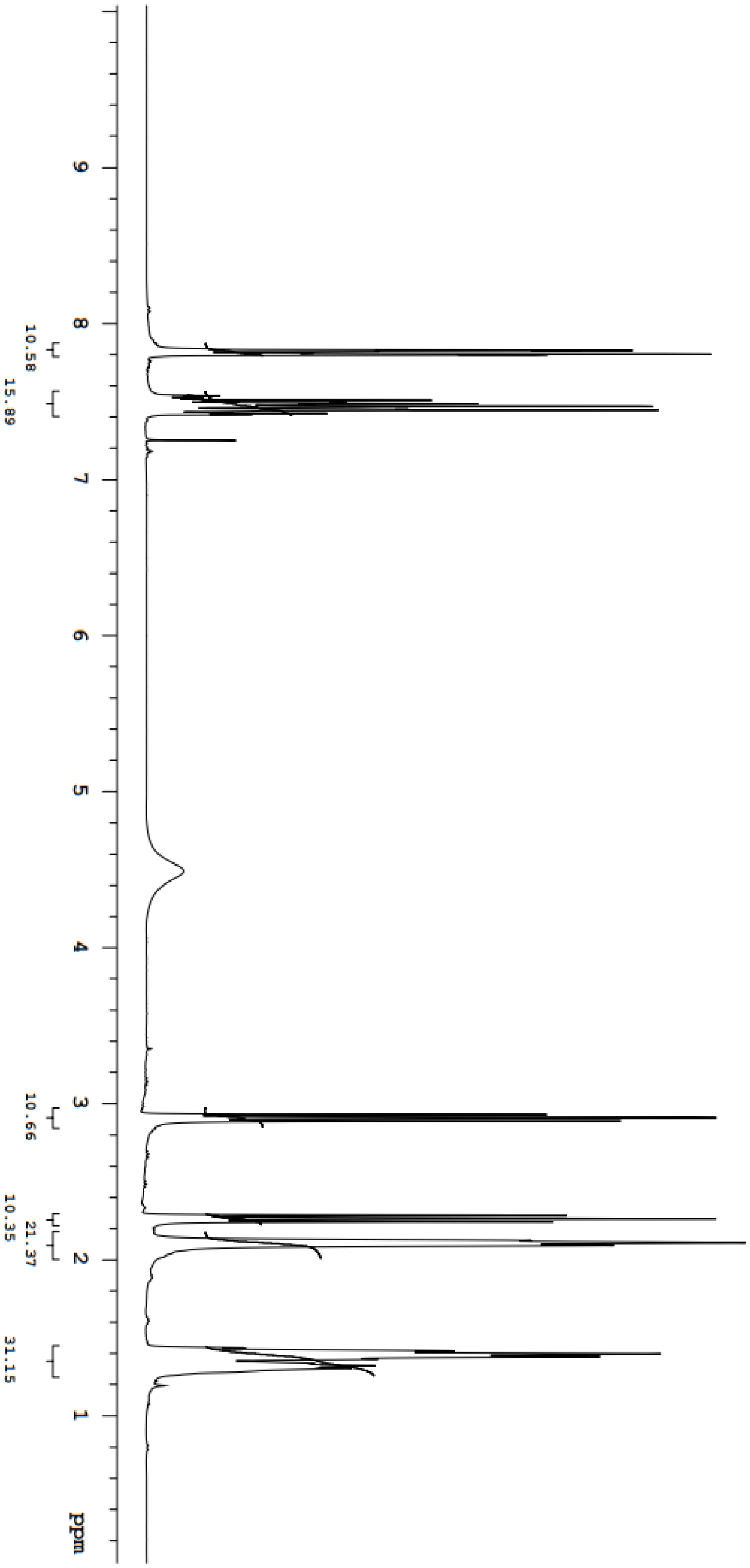

**Figure S19**: $^{13}$C-NMR spectra (C*D*Cl$_3$) for *N-(2-(piperidin-1-yl)ethyl)benzenesulfonamide,* **9**

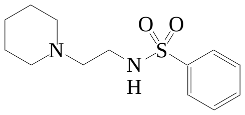


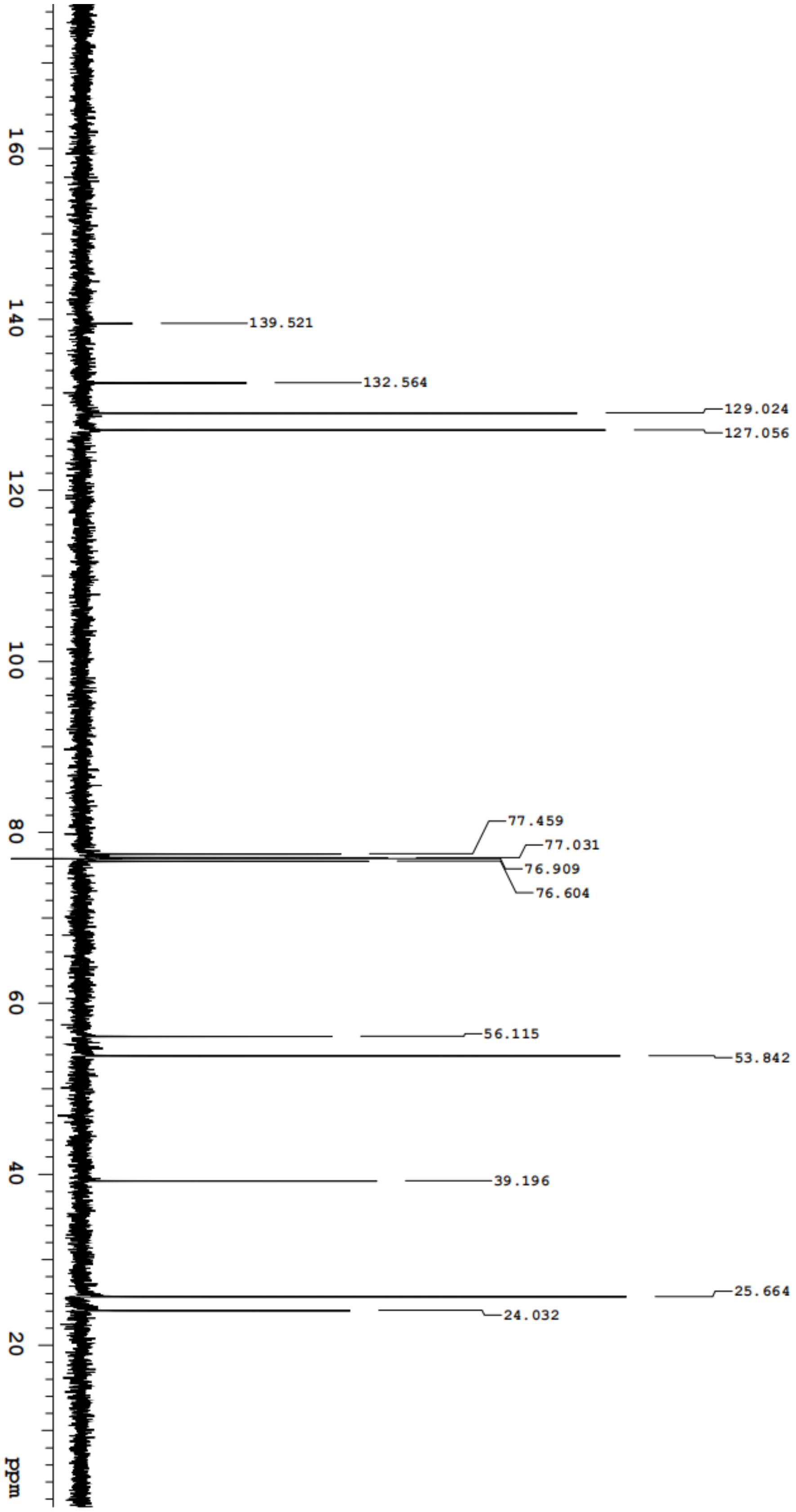

**Figure S20**: $^{1}$H-NMR spectra (C*D*Cl3) for *N-(2-(piperidin-1-yl)ethyl)-1-(p-tolyl)methanesulfonamide*, **10**

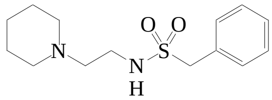


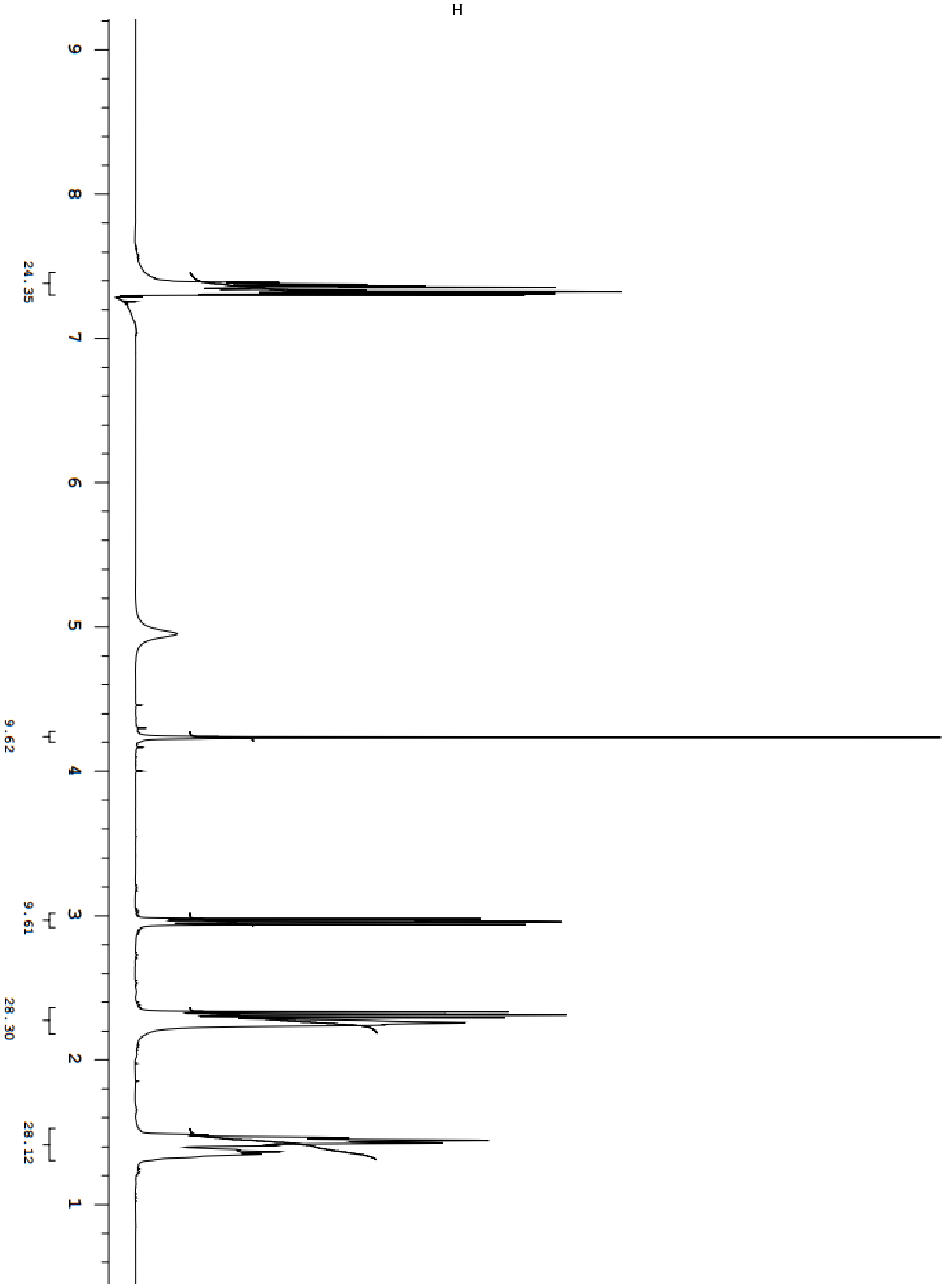

**Figure S21**: $^{13}$C-NMR spectra ($CDCl_3$) for *N-(2-(piperidin-1-yl)ethyl)-1-(p-tolyl)methanesulfonamide*, **10**

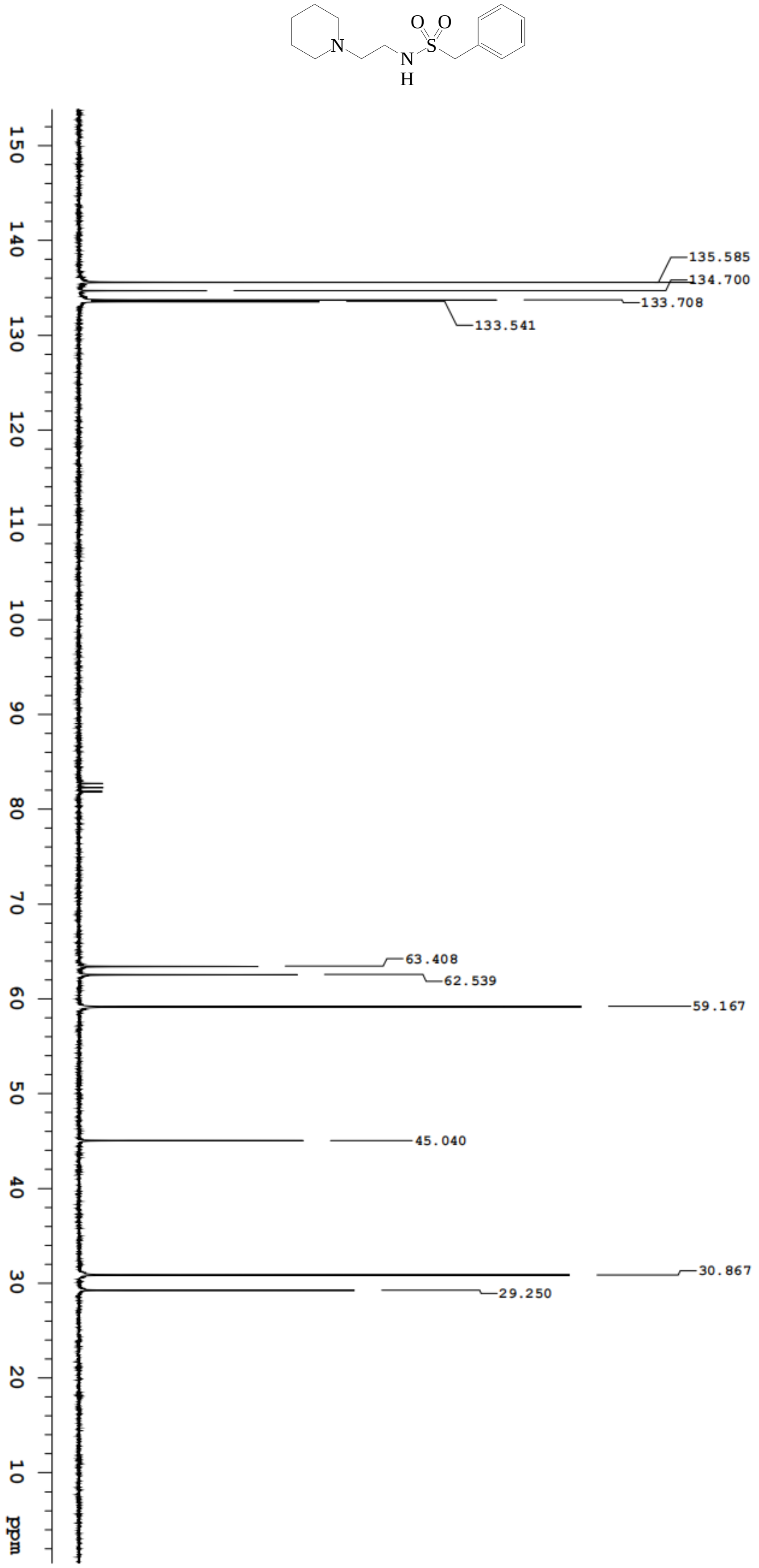

**Figure S22**: $^{1}$H-NMR spectra (C*D*Cl3) for *1-phenyl-N-(2-(piperidin-1-yl)ethyl)methanesulfonamide,* **11**

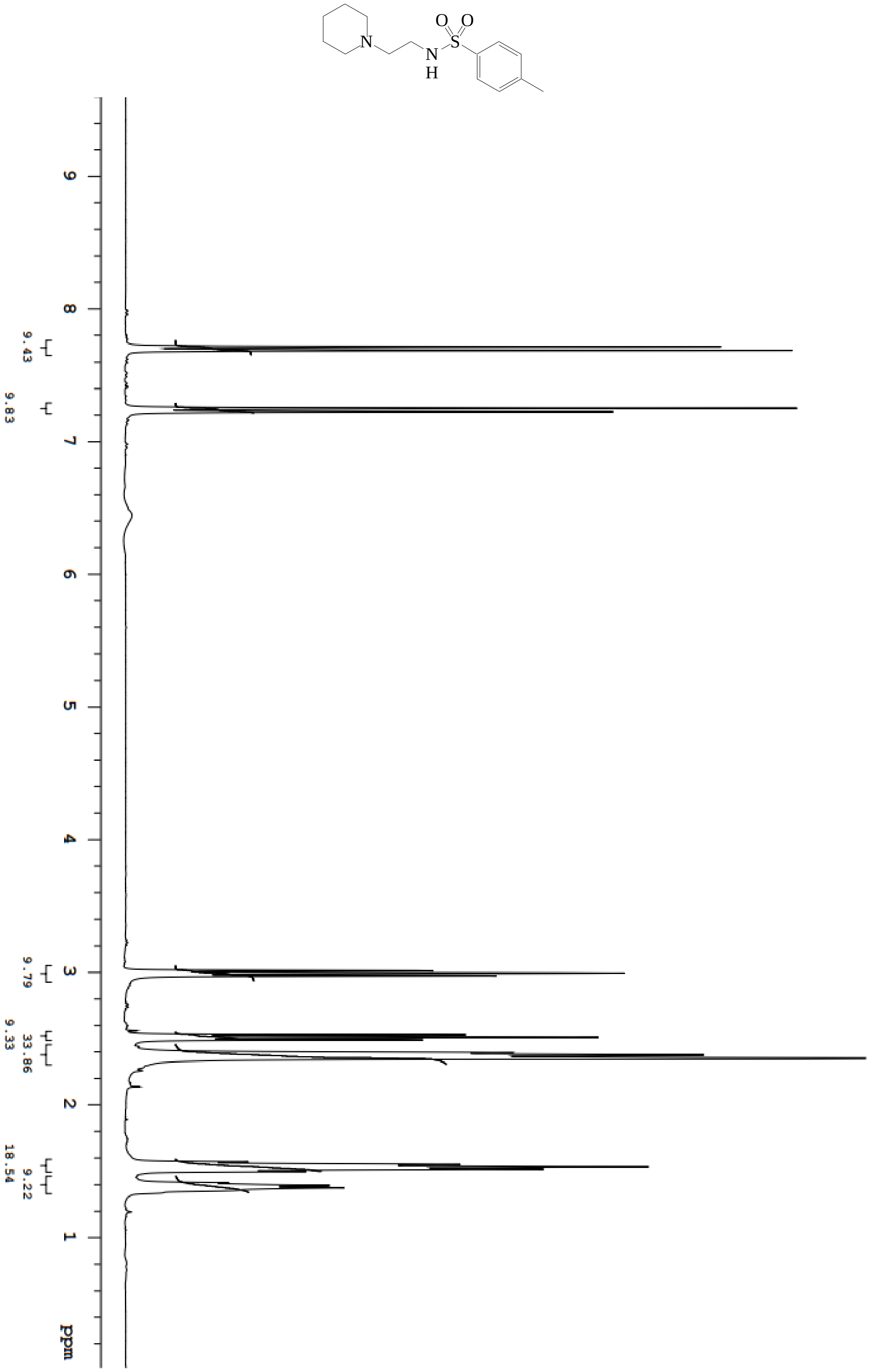

**Figure S23**: $^{13}$C-NMR spectra ($CDCl_3$) for *1-phenyl-N-(2-(piperidin-1-yl)ethyl)methanesulfonamide*, **11**

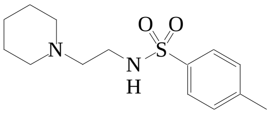


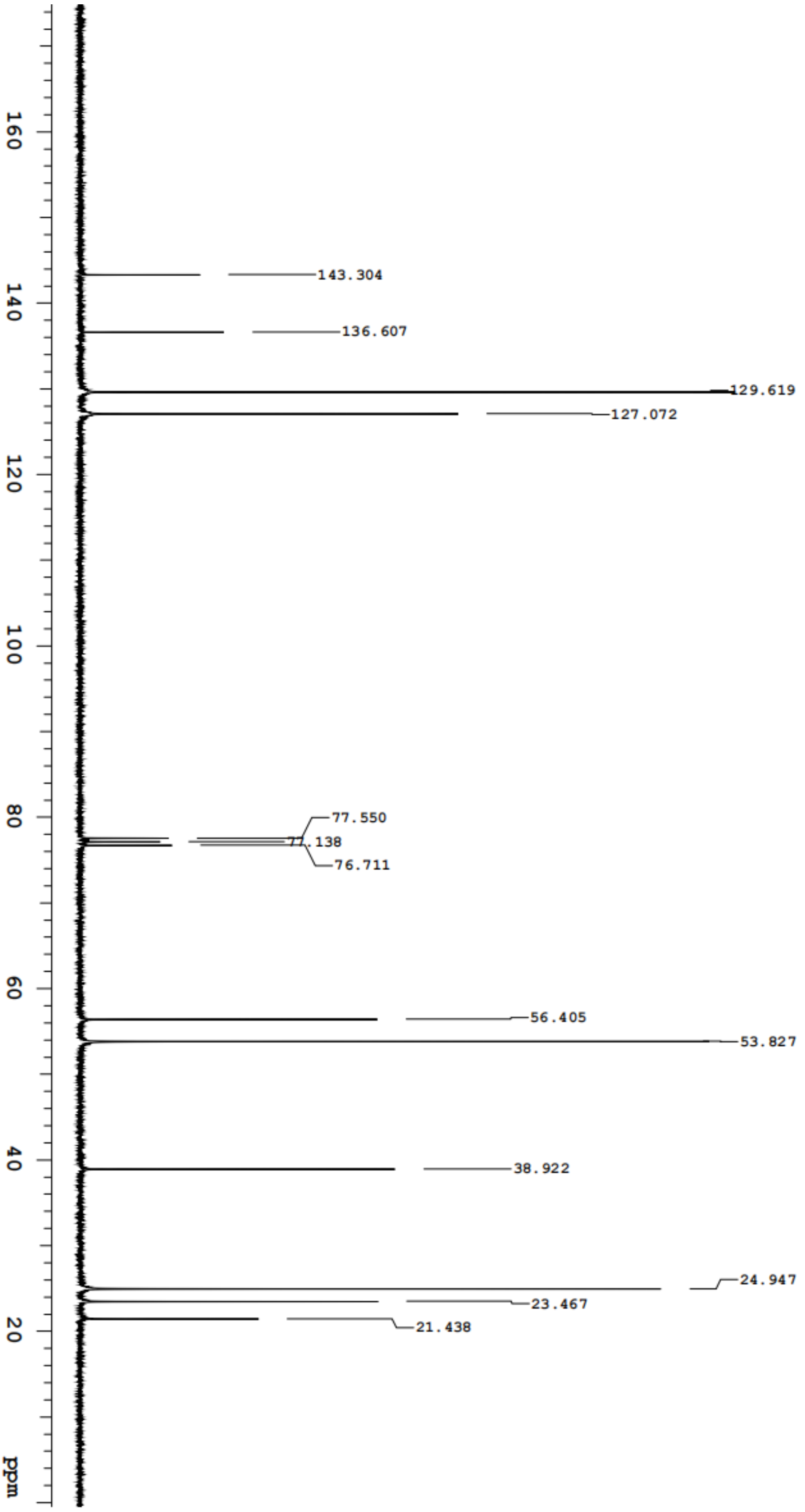

**Figure S24**: $^{1}$H-NMR spectra ($CD_3OD$) for *4-methyl-N-(3-(pyrrolidin-1-yl)propyl)benzenesulfonamide*, **12**

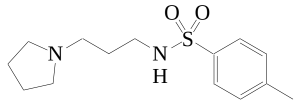


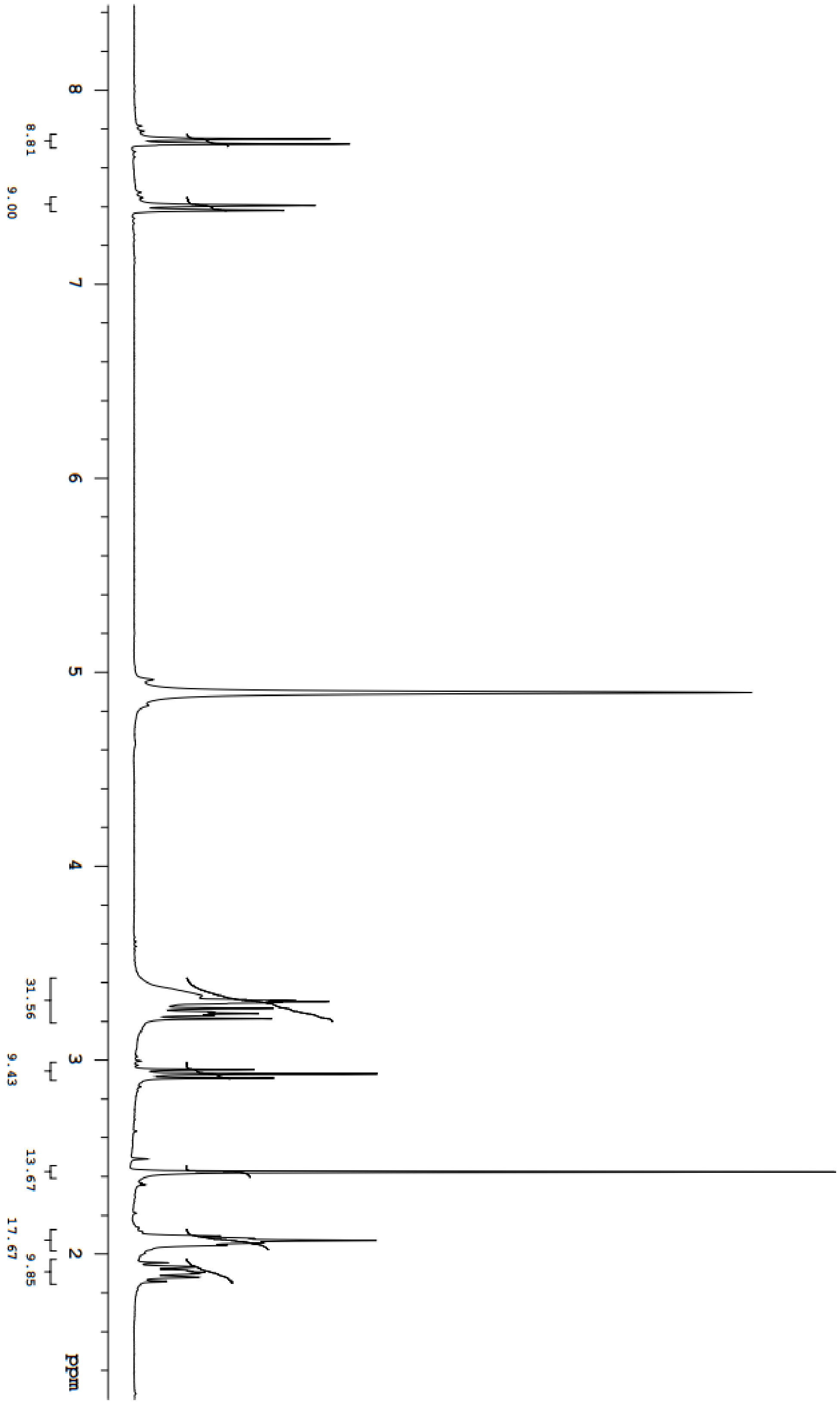

**Figure S25**: $^{13}$C-NMR spectra ($CD_3OD$) for *4-methyl-N-(3-(pyrrolidin-1-yl)propyl)benzenesulfonamide*, **12**

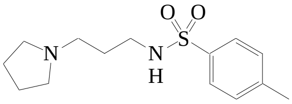


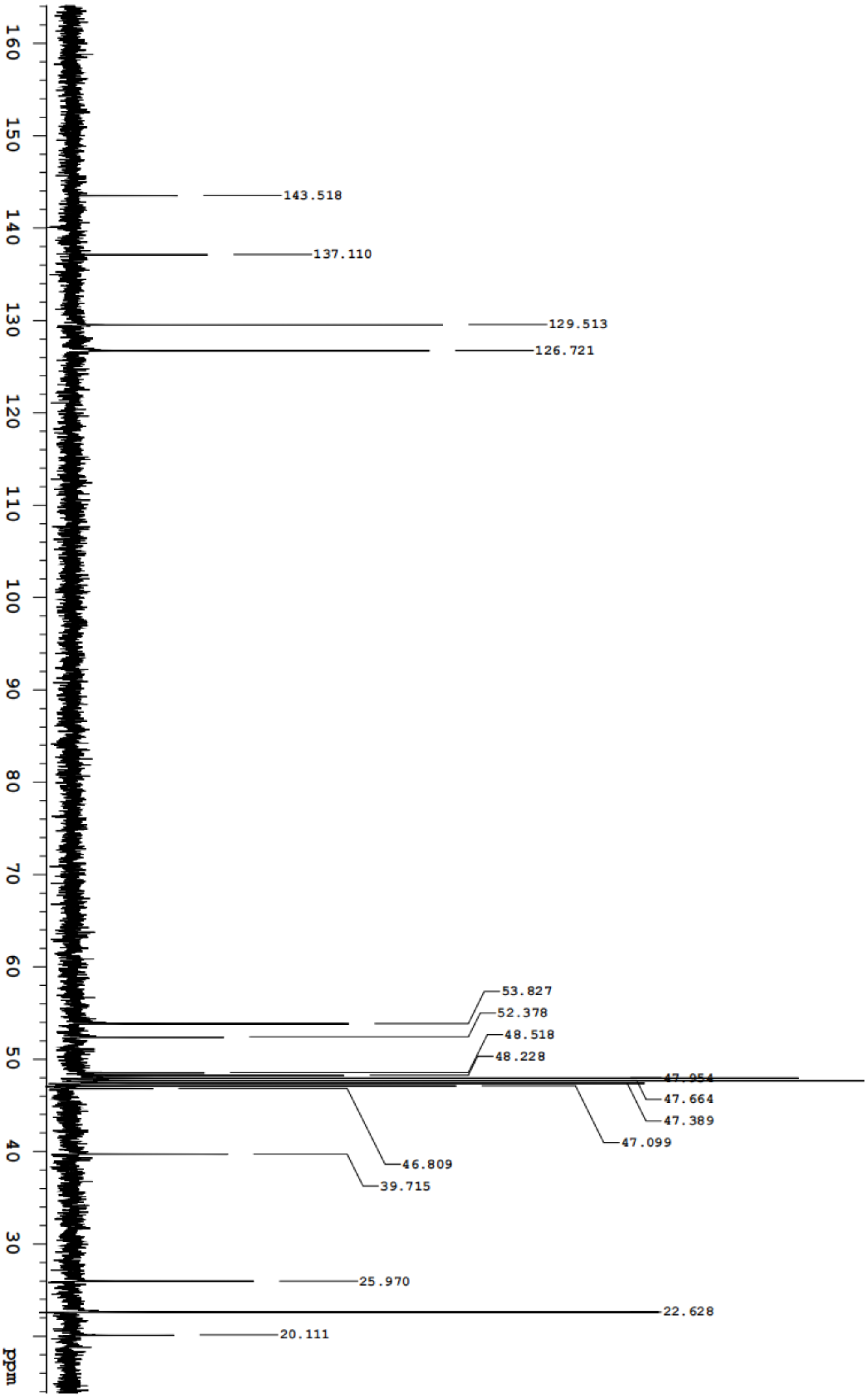

**Figure S26**: $^{1}$H-NMR spectra (C*D*3O*D*) for *N-(2-(1-methylpyrrolidin-2-yl)ethyl)benzenesulfonamide*, **13**

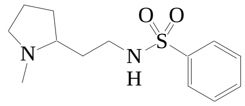


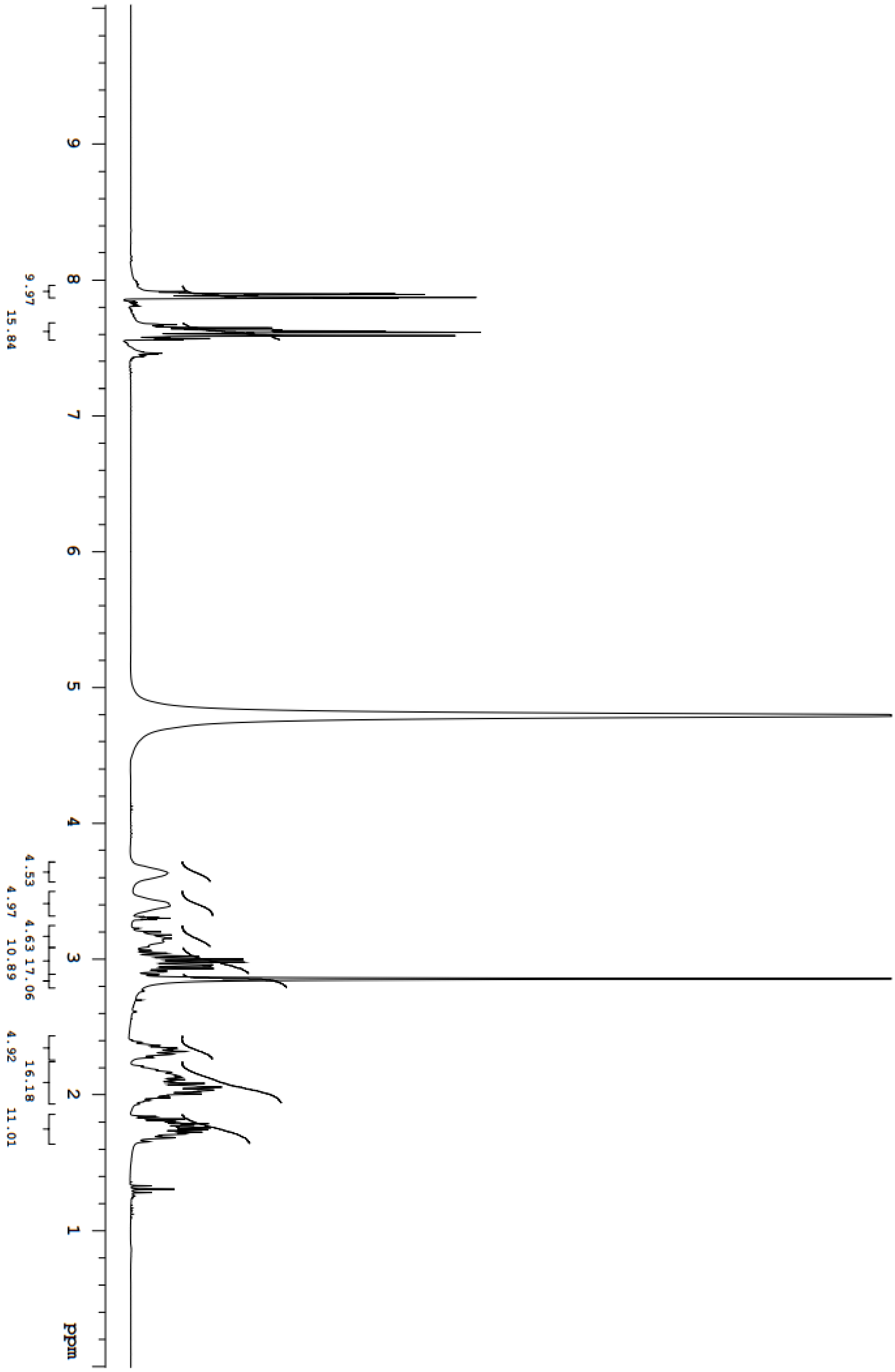

**Figure S27**: $^{13}$C-NMR spectra ($CD_3OD$) for *N-(2-(1-methylpyrrolidin-2-yl)ethyl)benzenesulfonamide*, **13**

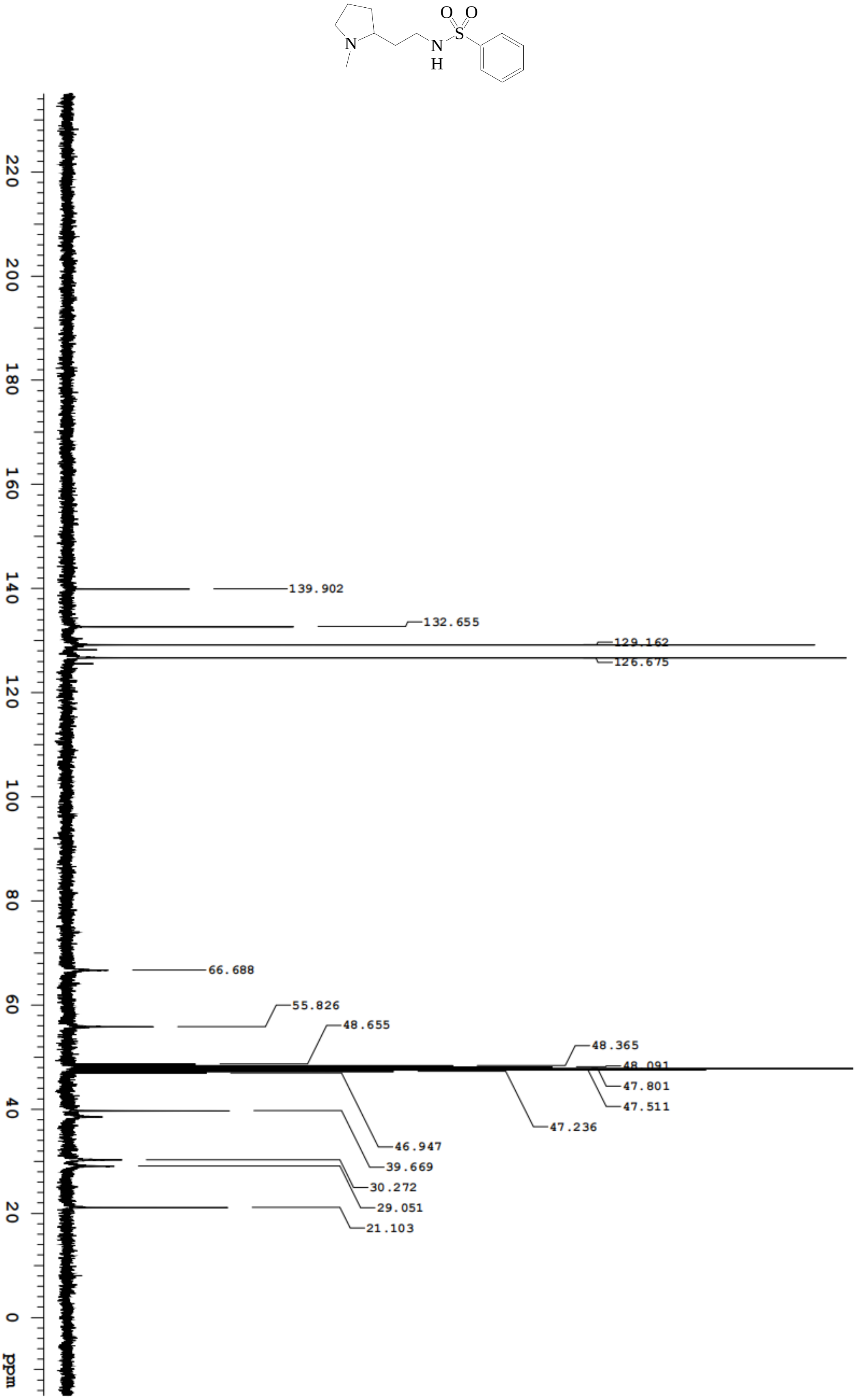

**Figure S28**: $^{1}$H-NMR spectra (C*D*3O*D*) for *4-methyl-N-(2-(1-methylpyrrolidin-2-yl)ethyl)benzenesulfonamide*, **14**

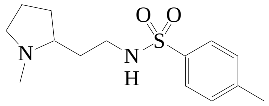

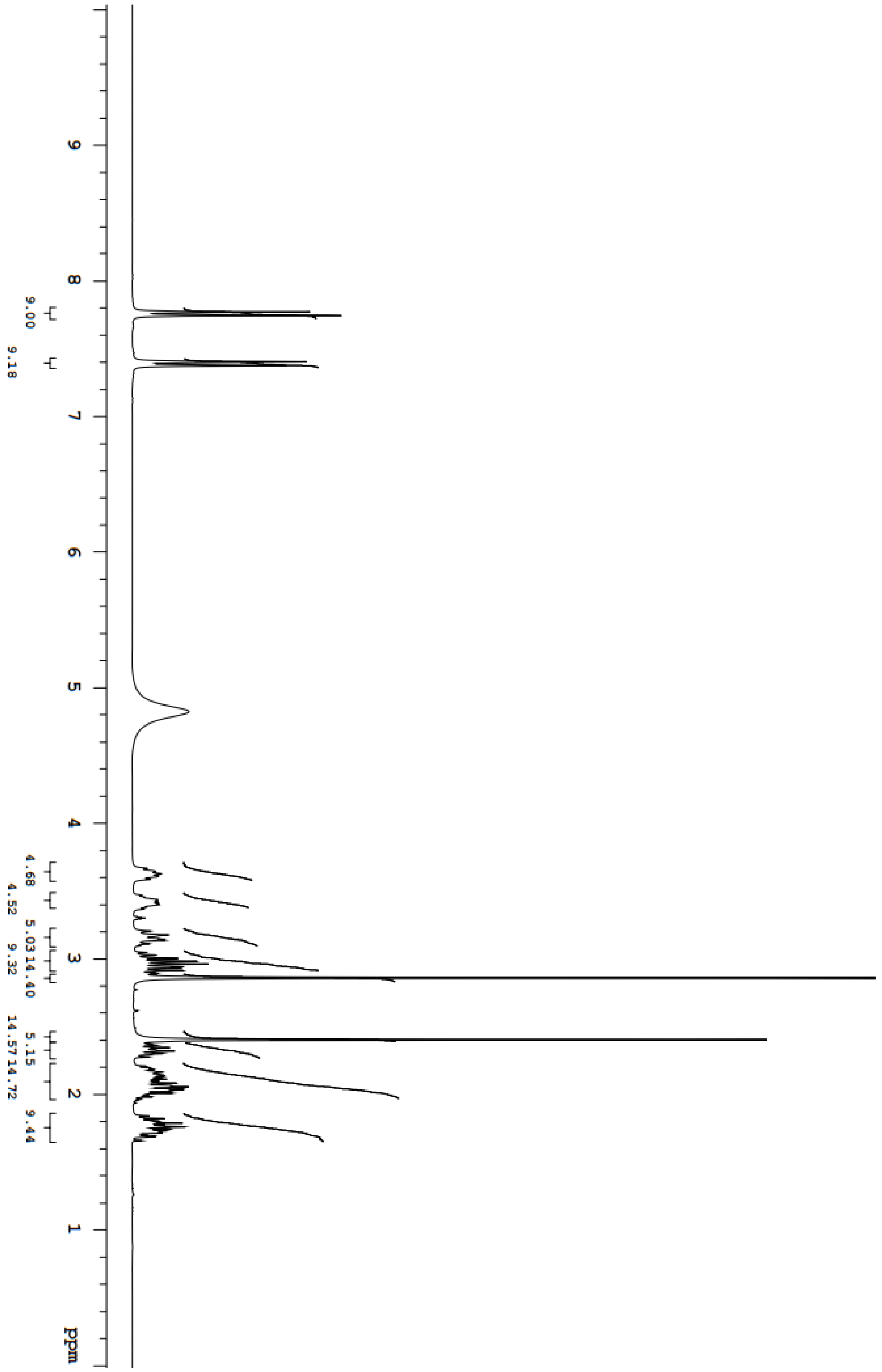

**Figure S29**: $^{13}$C-NMR spectra ($CD_3OD$) for *4-methyl-N-(2-(1-methylpyrrolidin-2-yl)ethyl)benzenesulfonamide*, **14**

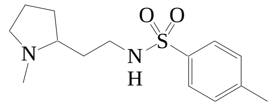


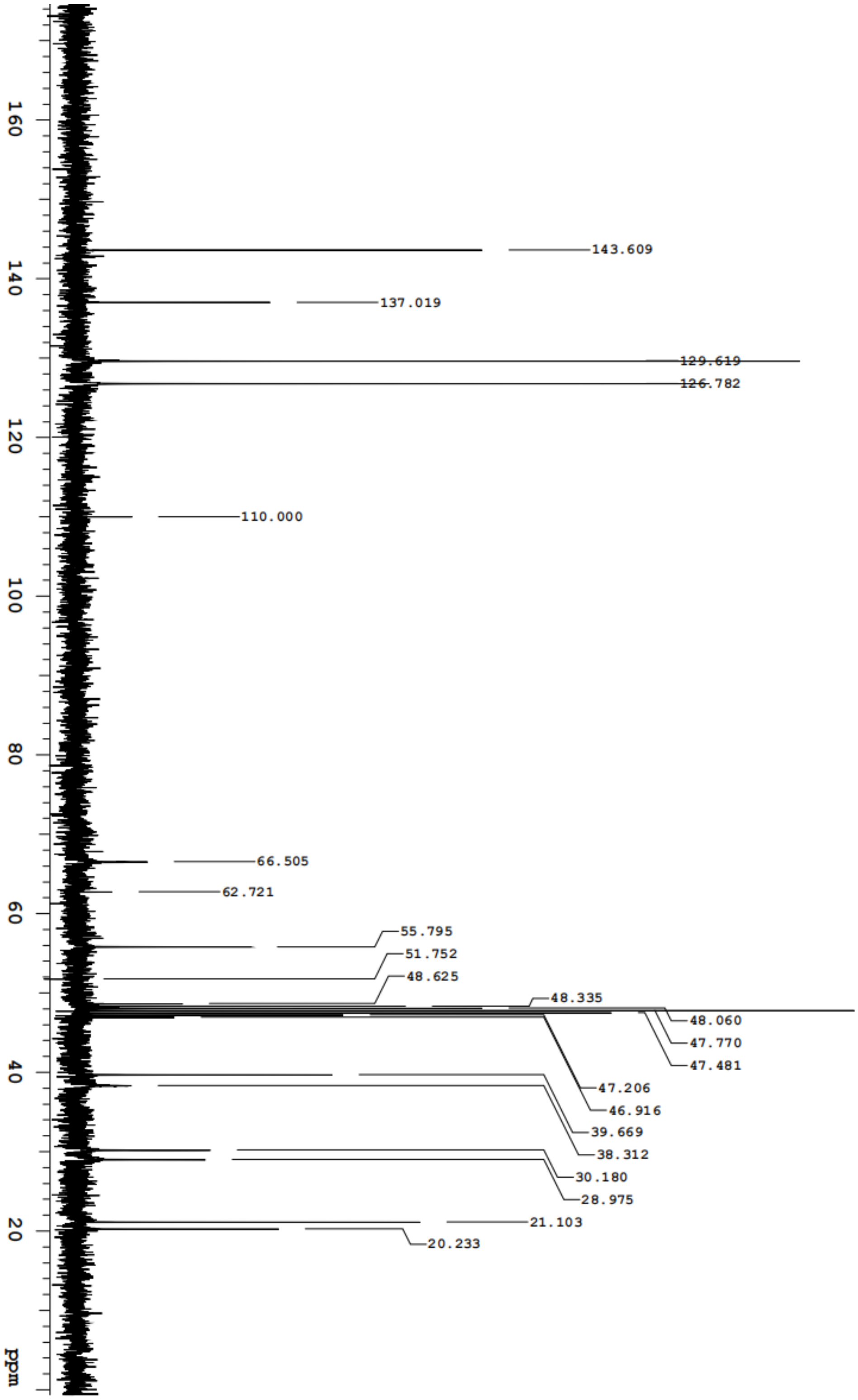

**Figure S30**: $^{1}$H-NMR spectra ($CDCl_3$) for *N-(2-morpholinoethyl)benzenesulfonamide*, **15**

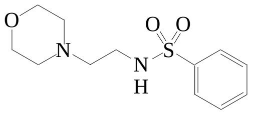


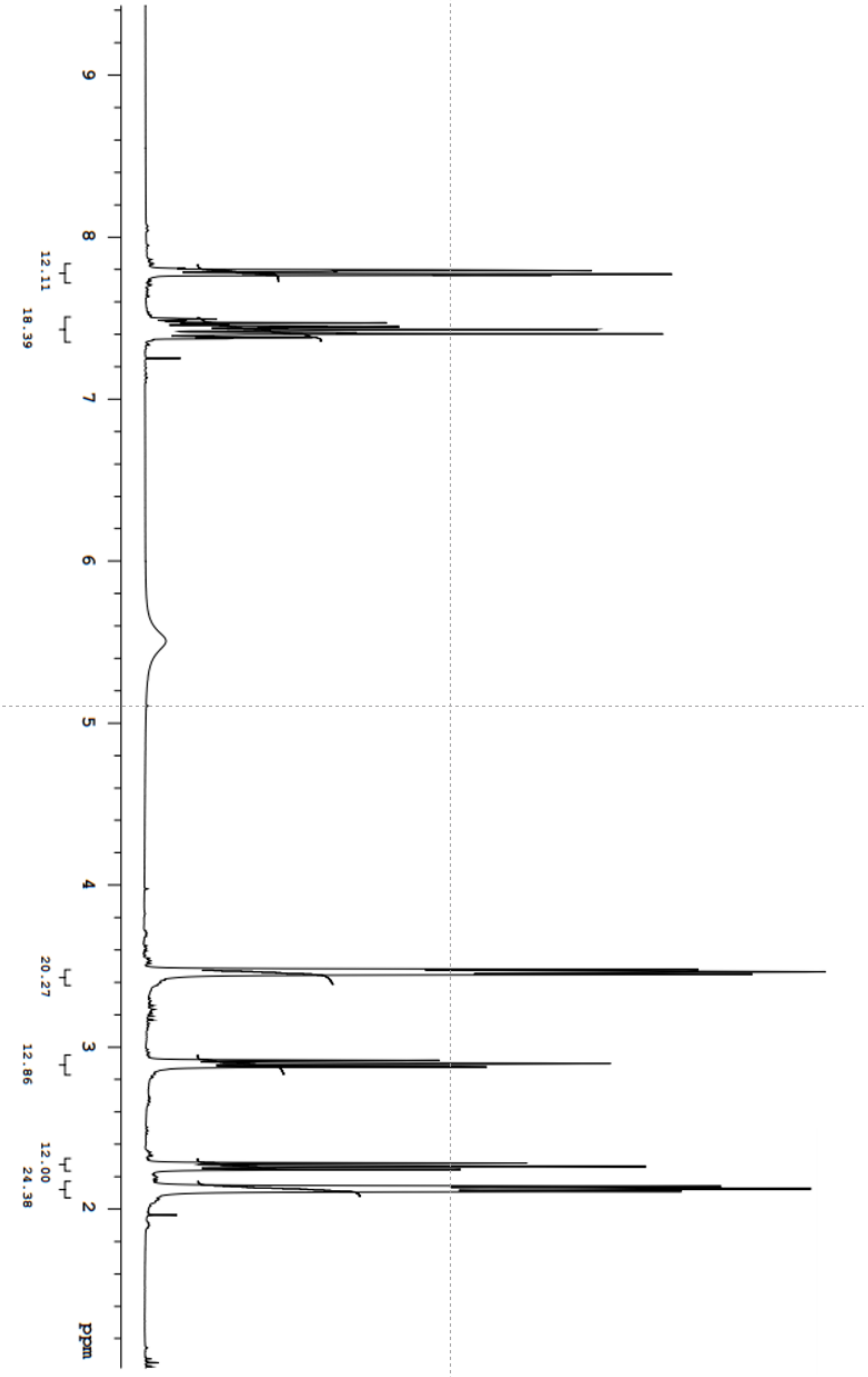

**Figure S31**: $^{13}$C-NMR spectra (C*D*Cl$_3$) for *N-(2-morpholinoethyl)benzenesulfonamide*, **15**

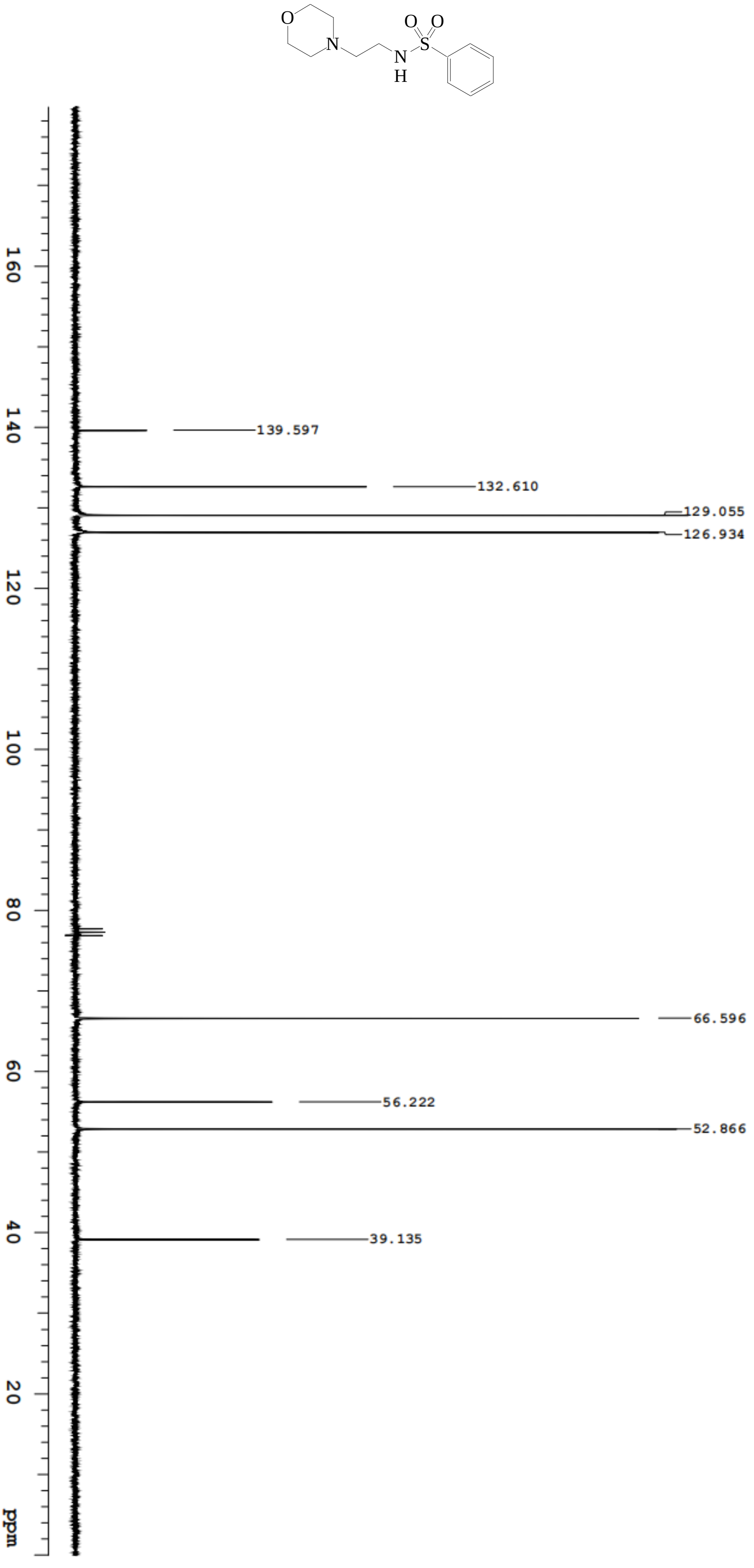

**Figure S32**: $^1$H-NMR spectra ($CD_3OD$) for *4-methyl-N-(2-morpholinoethyl)benzenesulfonamide,* **16**

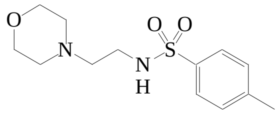


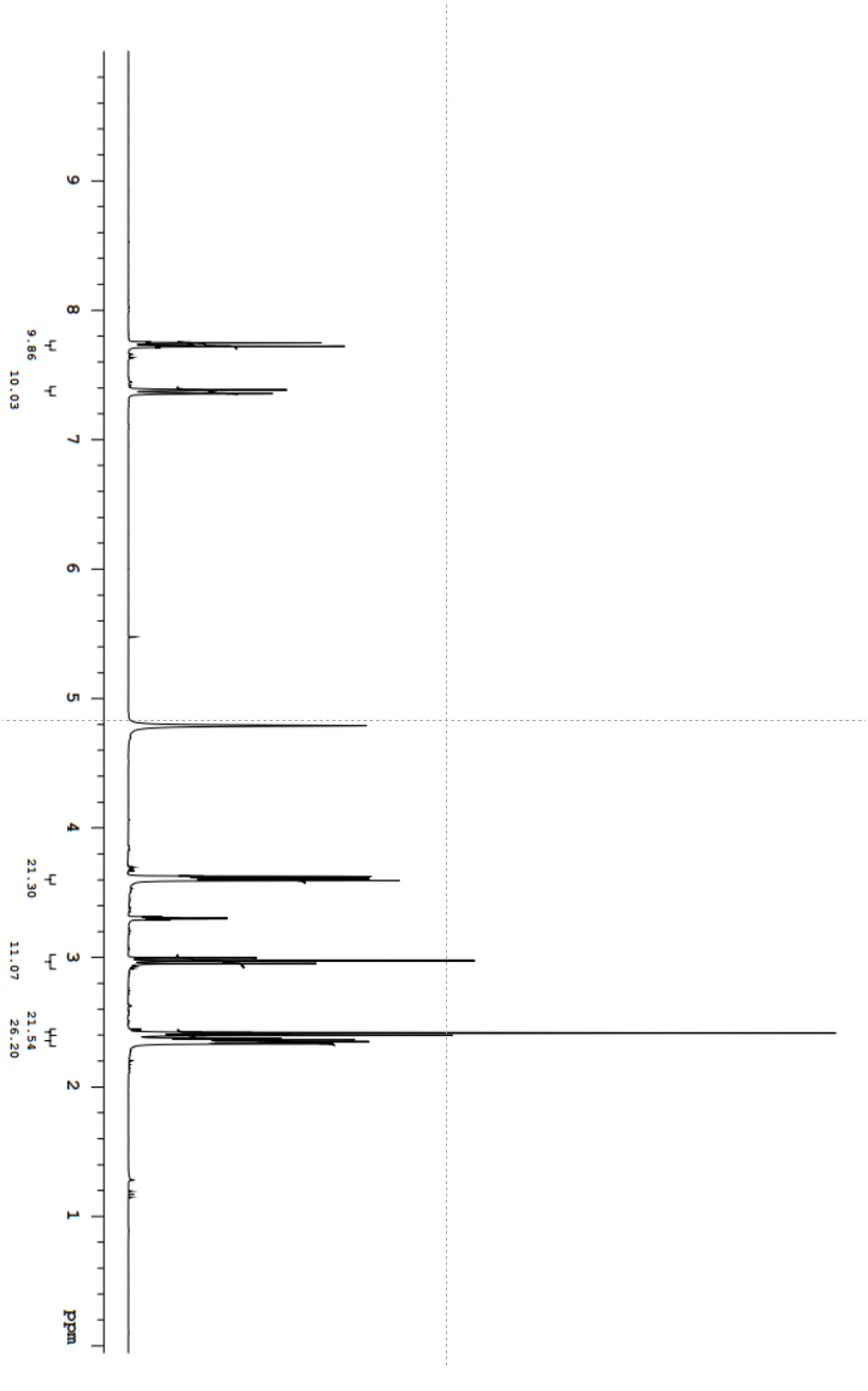

**Figure S33**: $^{13}$C-NMR spectra (C*D*$_3$O*D*) for *4-methyl-N-(2-morpholinoethyl)benzenesulfonamide,*
**16**

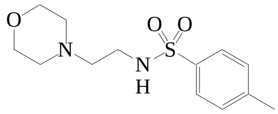


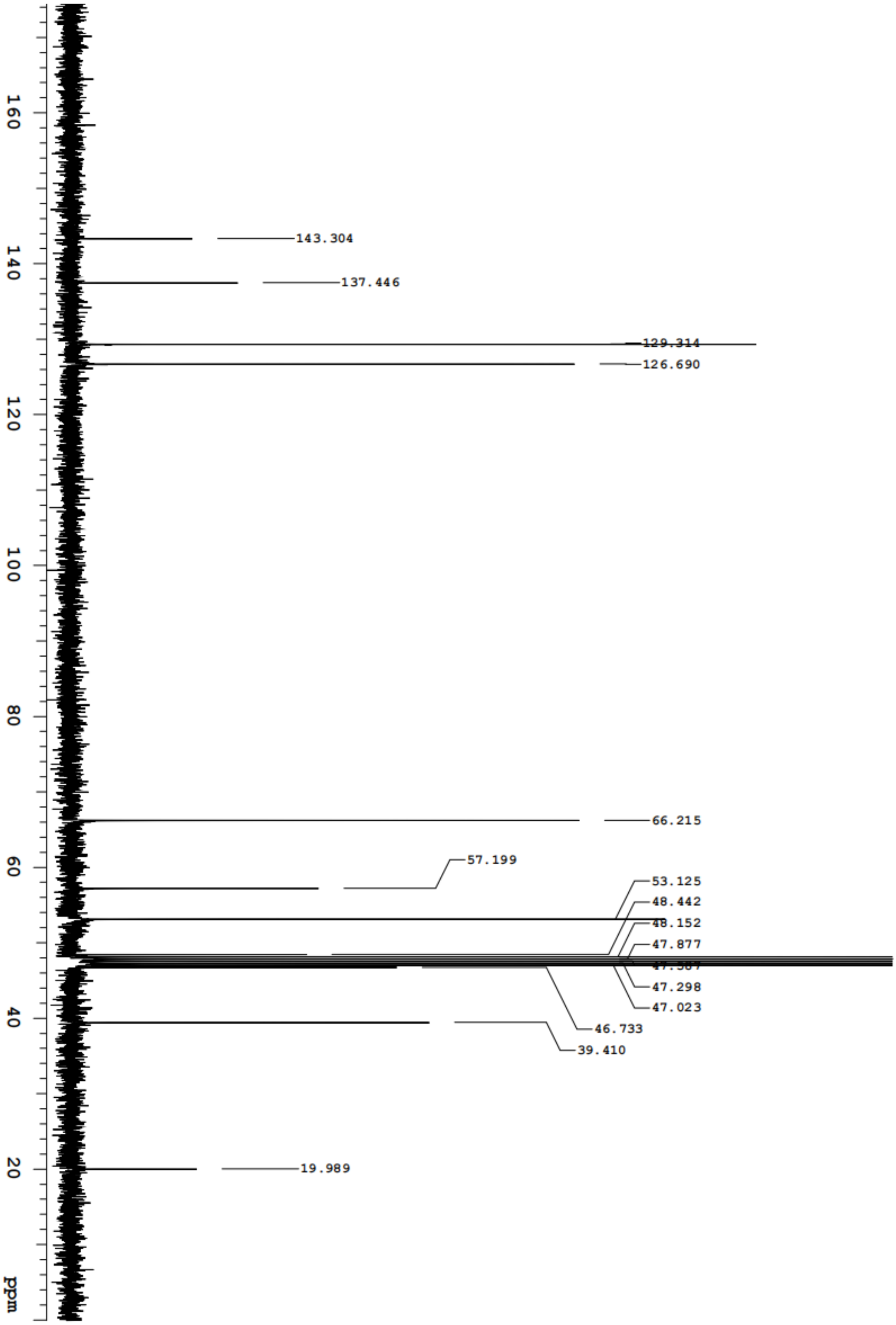

**Figure S34**: $^{1}$H-NMR spectra ($CDCl_3$) for *N-(2-morpholinoethyl)-1-phenylmethanesulfonamide,* **17**

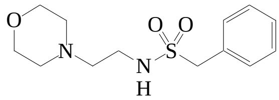

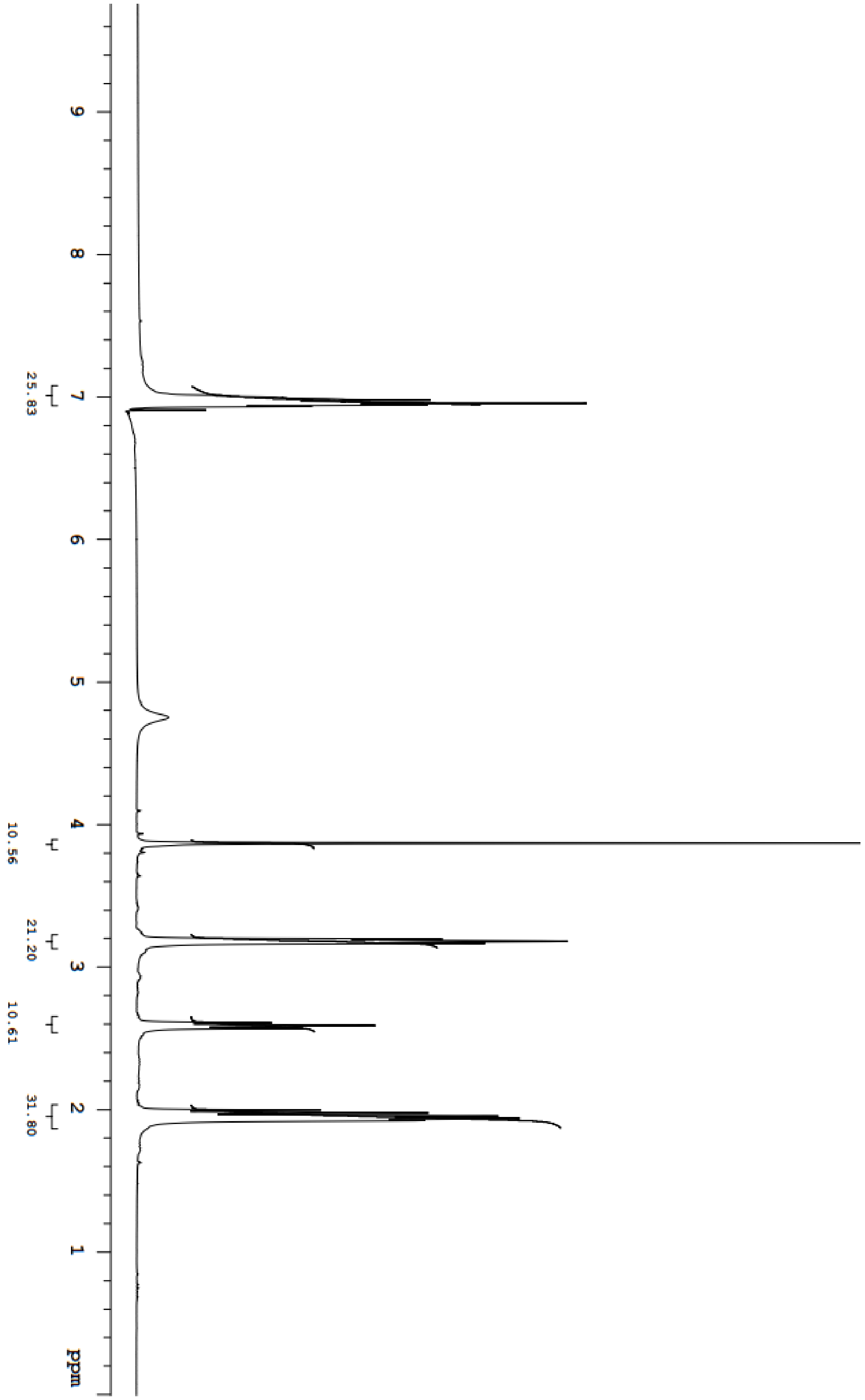

**Figure S35**: $^{13}$C-NMR spectra ($CDCl_3$) for *N-(2-morpholinoethyl)-1-phenylmethanesulfonamide,* **17**

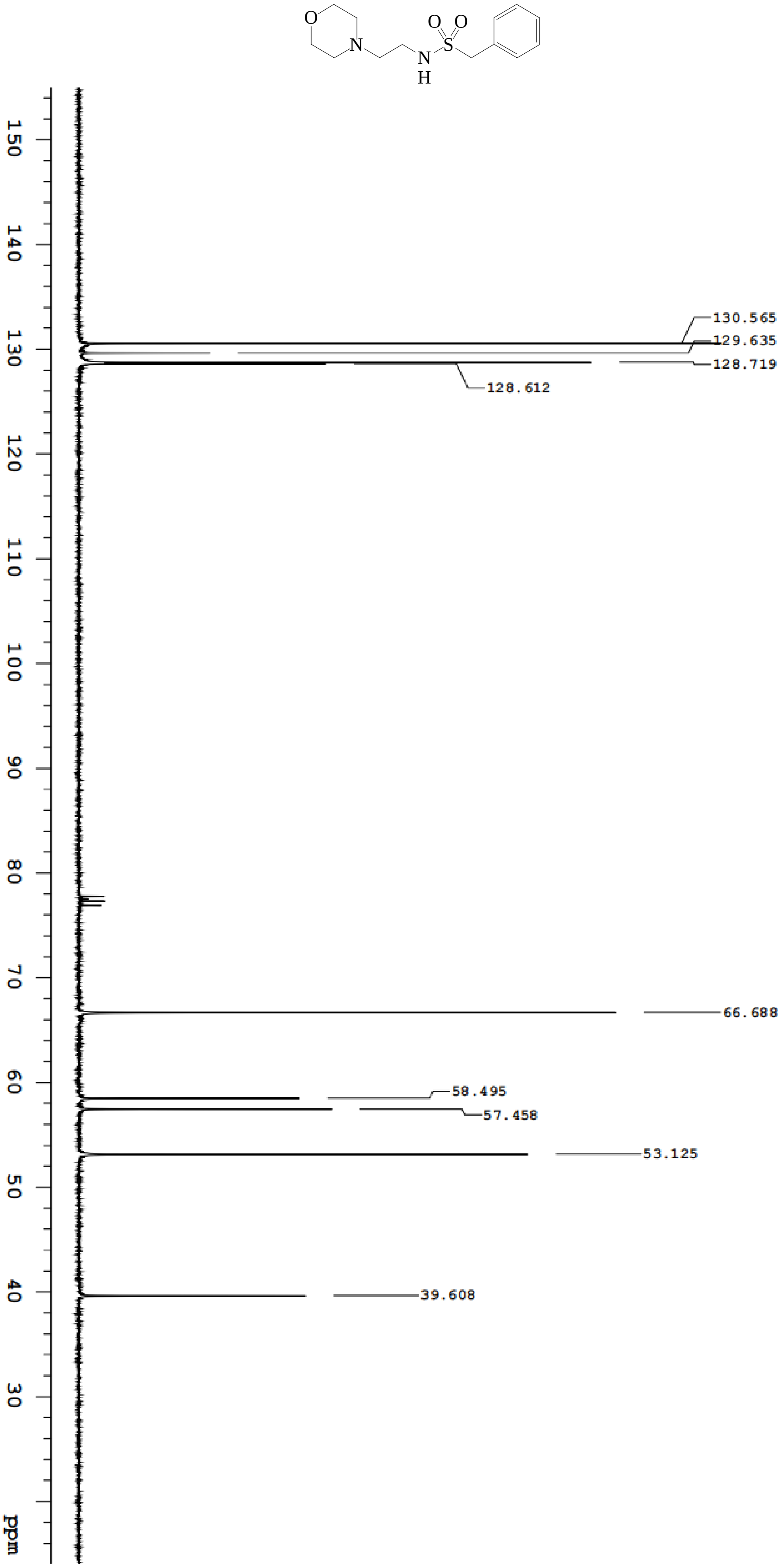

Supplement: Supplementary file 1 [file pharmaceuticals-18-01206-s001.zip › pharmaceuticals-3789404-supplementary.pdf]
